# Family Anesthesia Experience: Improving Social Support of Residents Through Education of Their Family and Friends

**DOI:** 10.15766/mep_2374-8265.11370

**Published:** 2023-12-15

**Authors:** Susan M. Martinelli, Thanh N. Tran, Brooke A. Chidgey, Robert S. Isaak, Emily G. Teeter, Fei Chen

**Affiliations:** 1 Professor, Department of Anesthesiology, Director, Anesthesiology Residency Program, and Co-Director, TEACHER Lab, University of North Carolina at Chapel Hill School of Medicine; 2 Research Assistant, TEACHER Lab, University of North Carolina at Chapel Hill School of Medicine; 3 Associate Professor, Department of Anesthesiology, and Division Chief, Pain Medicine, University of North Carolina at Chapel Hill School of Medicine; 4 Professor, Department of Anesthesiology, Vice Chair, Education, and Division Chief, Liver Transplant and Vascular Anesthesia, University of North Carolina at Chapel Hill School of Medicine; 5 Professor, Department of Anesthesiology, and Associate Director, Anesthesiology Residency Program, University of North Carolina at Chapel Hill School of Medicine; 6 Assistant Professor, Department of Anesthesiology, and Co-Director, TEACHER Lab, University of North Carolina at Chapel Hill School of Medicine

**Keywords:** Social Support, Wellness, Anesthesiology, Simulation, Well-Being/Mental Health

## Abstract

**Introduction:**

The prevalence of burnout among anesthesiology residents is 41%–51%. Burnout is associated with medical errors, physician turnover, and substance use disorder. Social support and wellness may reduce burnout, but a barrier is support persons’ lack of understanding of an anesthesiologist's work demands. We developed the Family Anesthesia Experience (FAX) to help support persons best support their resident.

**Methods:**

FAX consisted of a 4-hour event with hands-on experience, didactics portion, and panel discussion. Participants learned about a typical day in the life of an anesthesiology resident, wellness, burnout, substance use disorder, and available support resources, and had hands-on experience with procedures. The panel discussion offered logistical information about anesthesiology residency and allowed support persons to ask panel members questions. A postevent survey collected feedback on the event.

**Results:**

Fifty-one participants (first-year anesthesiology residents and their support persons) attended the event. Eight of 11 residents (73%) and 32 of 40 support persons (80%) completed the survey. All enjoyed the event, would recommend it to other anesthesiology resident support persons, and felt the event would improve communication and support. Most learned a moderate (35%) to large amount (50%) from the event. Qualitative feedback suggested most support persons found the event helpful in improving their understanding of anesthesiology residents’ work demands.

**Discussion:**

The FAX was well liked by participants. Although we did not assess specific knowledge gained and long-term effects of the 2022 event, evaluations of previous years’ events suggest that the event improved participants’ understanding of anesthesiology residents’ work and stressors.

## Educational Objectives

By the end of this activity, support persons will be able to:

1. Explain the role and responsibilities of an anesthesiology resident.
2. Communicate with their resident about work-related issues.
3. Describe ways to support their resident.
4. Describe how to reach the anesthesiology department with concerns about their resident.

## Introduction

Rates of burnout continue to be high for physicians (44%) and residents (36%-45%).^1–3^ Anesthesiology residents have proven to be no exception, with estimated burnout rates of 41%-51%.^4,5^ Unfortunately, physician burnout has tangible consequences, including financial costs such as physician turnover, decreased productivity, and medical errors.^6^ These costly medical errors are more frequent among residents and attendings at high risk for burnout in various specialties including anesthesiology.^4,7–9^ Another potential corollary of burnout can be substance use disorder, which remains a concern for anesthesiology residents.^10^

Psychological well-being has been linked to social relatedness, competence, and autonomy.^11^ The concept of social relatedness refers to one's ability to communicate with others in meaningful ways while feeling understood and valued by those around them. Social relatedness and social support have been linked to resident wellness and decreased burnout.^11^ We also know that when residents’ support persons (family and close friends) understand what leads to their work-related stress, it improves the residents’ wellness.^12,13^ Nevertheless, it can be quite difficult to communicate professional roles and responsibilities and corresponding work-related stressors to support persons who do not have a medical background.^14^

The Accreditation Council for Graduate Medical Education requires that residency programs educate faculty and residents on burnout, depression, and substance use disorders and provide access to care.^15^ Since many people do not understand the role of an anesthesiologist or, subsequently, an anesthesiology resident, we developed an experiential learning event called the Family Anesthesia Experience (FAX) that leveraged the use of simulation.^16^ Simulation is commonly used for specific task training but has not been broadly used in interventions that aim to improve social support and wellness. Social support has been a targeted intervention in the field of aviation and has led to improved pilot wellness.^17,18^ Previous wellness or burnout interventions among residents have focused on building resilience and recognizing behaviors related to burnout, but none have used simulation to help support persons understand the demands of anesthesia in order to improve communication and support.^19,20^

Our program taught support persons alongside their residents about the expectations and requirements of an anesthesiology resident and the clinical role of an anesthesiologist so that support persons could visualize potential stressors experienced by their resident. We also shared important information regarding burnout, substance use disorder, and resources to ensure that support persons had adequate knowledge and tools to best support their residents. By educating support persons about the stressors involved in their residents’ daily lives and inviting them into the world of residency, we hoped to open lines of communication and understanding. Because support persons may be the first to notice changes or problems with their residents, it is important that they be comfortable reaching out to program leadership with concerns. We ultimately hoped to improve support in residents’ personal lives in order to reduce burnout and increase wellness.

## Methods

This project was determined by the University of North Carolina Institutional Review Board (IRB) to be non-human subjects research and did not require IRB approval (Study #23-1812).

We held the FAX for our first-year anesthesiology (CA-1) residents four times in person (2017–2019 and 2022). Due to the COVID-19 pandemic, we transitioned the event to a virtual platform in 2020 and 2021. Here, we describe our latest in-person event experience in 2022, which was also our first in-person event since the outbreak of COVID-19. A detailed description of the virtual event has been reported separately.^21^ Based on feedback from participants (support persons and CA-1 residents) at past events, we made small changes to the in-person event over time to optimize engagement.^22,23^ For instance, we shifted from an afternoon event with dinner to a midday event with lunch. We removed a didactics session on financial wellness. The hands-on component was shifted to an earlier portion of the event, and the didactics were moved after lunch.

### Date Selection for the Event

We held our event on a Saturday to facilitate the attendance of support persons who may have had a timing conflict during the week and/or lived out of town. We chose a date early in the academic year so the support persons would have access to the provided information toward the beginning of their resident's anesthesiology training. We limited CA-1 residents’ call obligations the night before and on the day of the event. Additionally, we sent a Save-the-Date email to our residents 4 months prior to the event to facilitate attendance from out-of-town support persons.

### Personnel

Four faculty members were needed to run the event (Appendix A). For implementations at other institutions, we highly recommend involving program leadership. Faculty delivered didactics on wellness and burnout, substance use disorder, and available local resources. The same speaker delivered didactics on wellness, burnout, and local resources. One faculty member moderated the senior resident/support person panel. These faculty members also assisted in the simulation stations.

Three upper-level (CA-2 or CA-3) residents and their support persons participated in the panel. It was important to have a diverse group of residents (relationship and parental status, gender, race, etc.) to best represent the program, and we also we aimed for a diverse group of support persons including variety in their role (parent, spouse, etc.). The goal was for all participants to see themselves as a panelist. These residents also helped run the simulation stations.

Additional support was helpful. Three standardized patients were utilized in the high-fidelity scenario (one as the patient, one as a nurse, and one as a surgeon). Other support staff helped with setup, breakdown, and rotation of participants through the simulation stations.

### Setup

Our event was held in a simulation center, although it could be done elsewhere. The main room had enough chairs for all participants and audivisual equipment for the didactic components of the event and the panel presentation. Three additional rooms were used for the simulation stations. Two of these rooms were staged with task trainers. The airway room had mannequin heads on tables with self-inflating resuscitator bags, masks, laryngeal mask airways, endotracheal tubes, oral airways, laryngoscopes, and a video laryngoscope. The procedure room had task trainers for the central line, epidural, and peripheral nerve block placement set up on separate tables. Ultrasounds were available at the central line and peripheral nerve block stations. The third room accommodated a high-fidelity operating room scenario demonstration with chairs for the participants to observe. Detailed description of the setup of the rooms and the simulation stations can be found in Appendix B.

### Folder for Support Persons

We provided folders for each support person attending the event. The folders had a schedule of the day (Appendix C), a photo composite of the CA-1 class, and a sheet that contained the pictures and contact information of all the program directors.

### Event

Our event ran from 10:00 AM to 2:00 PM (refer to Appendix D for the timeline). We asked participants (support persons and CA-1 residents) to arrive 15 minutes early to sign in and apply name tags. We distributed folders to the support persons upon arrival.

We started with a brief introduction and showed our 5-minute Day in the Life video (Appendix E; this video is author owned). The video showed a CA-1 resident setting up the operating room, attending lecture, conducting a preoperative interview, and monitoring the patient during and after surgery. One of the upper-level residents moderated this video. For future implementations at other programs, we encourage each program to create their own video to show the spaces where residents work and learn, but ours can be used if necessary.

Following the video, a faculty member provided a brief description of the simulation stations. The three stations included airway management, procedural task trainers, and a high-fidelity scenario (Appendix B). We split the CA-1 residents and their support persons into three groups ahead of time, keeping CA-1 residents in the same group as their support persons. Participants spent 30 minutes at each station

In the airway session, the CA-1 residents taught their support persons airway management skills and facilitated the support persons’ management of the airway on the mannequin heads. A faculty member supervised and assisted with teaching as needed.

In the procedure room, a faculty member or upper-level resident staffed each of the task trainer stations and guided hands-on experience for the support persons and CA-1 residents.

The third station required a faculty member, an upper-level resident, and a standardized patient. The high-fidelity scenario (Appendix F) started with a preoperative assessment of the patient by the resident. The scene transitioned to the operating room where the induction of anesthesia occurred, followed by an intraoperative code with a positive resolution and phone handoff report to the ICU. We ran the preoperative assessment part of the scenario live, using a standardized patient. The intraoperative part of the scenario was shown as a video (Appendices G and H; these videos are author owned) because of our small simulated operating room space, but ideally, this would be done live. Since we showed the intraoperative portion of the scenario on video, we provided a tour of the simulated operating room before starting the scenario.

Following the high-fidelity scenario, the faculty and senior resident debriefed and answered questions from CA-1 residents and their support persons. The debrief generally focused on the support persons’ questions about what they saw, how they felt, or how they perceived this situation might affect their resident. Talking points corresponding to each session are found in Appendix I. Additional faculty, residents, or other helpers were used to assist with teaching, take photos, keep time, and direct participants to different stations.

We provided a box lunch following the simulation stations. This time allowed for socializing between residents, support persons, faculty, and upper-level residents.

After lunch, the faculty delivered brief didactic presentations. We discussed wellness and burnout, substance use disorder, and the resources available at our institution and within our department (Appendix J). The didactic PowerPoint slides in Appendix J have speaker notes and references to assist future presenters. During this time, we emphasized the accessibility of the program leadership and empowered the support persons to reach out to the program directors if they had any questions or concerns. We pointed out to the support persons that we had included our photos and contact information in their folders for future reference.

The final component of the event was the panel of senior residents and their support persons. The faculty moderator had a list of prepared questions (Appendix K) that had been shared with the panelists ahead of time. We discussed information about scheduling vacations, weekend work requirements, the board certification process, duty hours, and challenges faced by residents and support persons. We also solicited questions from CA-1 residents and their support persons, both written on notecards and asked directly.

When we closed the event, we thanked all participants for joining us and asked them to fill out a written survey for feedback on the event (Appendix L). To create the survey, we shortened a previous assessment of the FAX using questions on attendees’ overall perception of the program, evaluation of the components of the event, and open-ended questions to collect feedback.^23^ The survey typically took less than 2 minutes to complete and enabled us to continue refining and adapting the program in light of evolving contexts.

## Results

The 2022 event had the largest number of participants, with 11 CA-1 residents (out of a possible 16) and 40 support persons in attendance (Table 1). Five CA-1s did not attend (two had call obligations, one was on parental leave, one had a social obligation, and one did not provide a reason). Based on the 2022 postevent survey, eight respondents (20%) were residents, and 32 (80%) were support persons. Among the 32 support persons, eight (25%) were parents, eight (25%) were partners, 12 (38%) were related in other ways, and four (12%) were residents’ friends. About 41% of respondents lived with their resident/support person, 18% lived within a 2-hour drive from their resident/support person, 18% lived within a 2- to 8-hour drive from their resident/support person, 16% lived more than an 8-hour drive away but in the U.S., and 7% lived abroad.

**Table 1.**
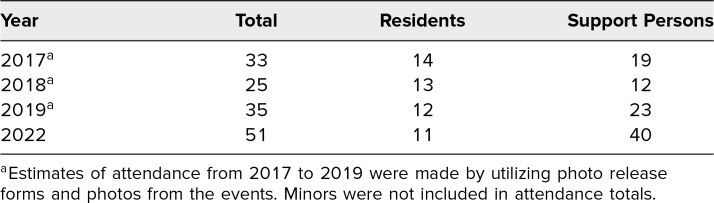
Family Anesthesia Experience In-Person Event Attendance History

In the postevent survey, we asked four questions on a 4-point Likert-type scale to gauge participants’ attitudes toward the event (Table 2). All respondents either strongly agreed (72%) or agreed (28%) that they enjoyed participating in the event. All respondents would recommend this event to other support persons of anesthesiology residents. All respondents either strongly agreed (80%) or agreed (20%) that the event would improve communication and support between the resident and support person. Most respondents reported that they learned either a large amount (50%) or a moderate amount (35%) from this event, while 12% responded that they learned a small amount and one person (2%) did not learn anything. Components of the event that participants highly favored were the anesthesiology procedures and airway management stations, followed by the Day in the Life video, high-fidelity scenario, and senior resident support person panel. In answer to the question “What is the most helpful piece of information gained?”, many respondents referenced the simulation stations that offered physical demonstrations of procedures anesthesiologists frequently perform. Overall, participants found that the event helped them to understand what CA-1s do, how stressful their position is, and to recognize their need for social support.

**Table 2.**
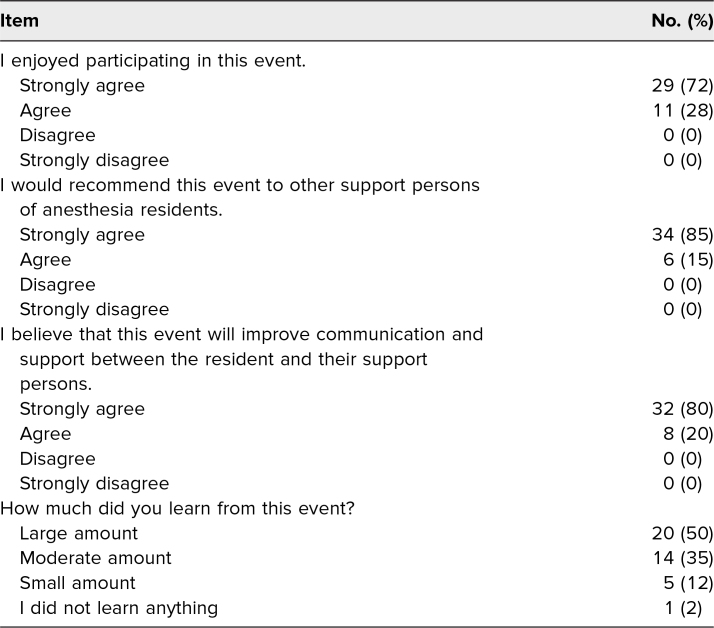
Attitudes Regarding the 2022 Family Anesthesia Experience

## Discussion

To increase new anesthesiology residents’ social support and improve their wellness, we developed the FAX to teach support persons alongside their residents about anesthesiology residency. Assessments of previous FAX events were published in 2020. Similar to our 2022 iteration, those earlier support persons enjoyed the event and would recommend it to others. Additional data were collected and reported for previous versions of the FAX. After attending the event, support persons reported an increase in their understanding of the role of an anesthesiology resident, knew how to support their resident, and knew how to reach out to the anesthesiology department if they had concerns about their resident. The CA-1 attendees believed it would be easier to communicate with their support persons about work-related issues after the event. Furthermore, we found that the event led to improved understanding among support persons regarding anesthesiologists’ work and potential stressors.^22,23^ We also found significant associations between the event and decreased stress (8 months postevent) among residents who had attended the event in comparison to those who had not.^23^ Although these items were not measured in the 2022 FAX, the outcomes should have remained similar as the revisions to the program were primarily related to flow and logistics.

We were excited to return the FAX to an in-person event after 2 years on a virtual platform due to the COVID pandemic. Interestingly, the 2022 event was the highest-attended in-person event we have hosted. The expansion of our residency class from 14 to 16 CA-1 residents did not account for the near doubling in attendance. Encouragement from senior residents and attention from prior publications about this event may have promoted attendance.^22,23^ Additionally, the opportunity for support persons to spend time in person with their residents and their residents’ coworkers may have had more appeal following only virtual events during the pandemic. The large number of attendees and attendees who traveled long distances for the event suggests that there may be a need to teach the public about the role of an anesthesiologist, which is an enigma to most.

After experiencing both in-person and virtual events, we found the in-person version to be more valuable. When virtual, we showed videos of someone performing the procedures and narrated the process. However, feedback from attendees suggested that participants highly value hands-on experiences that can only be offered in person. The in-person format also facilitated networking between faculty, residents, and support persons, which we believe is an invaluable opportunity to foster trust and relationship building within our community. There were some benefits to a virtual platform, including its less resource-intensive nature and the ease of participation for support persons unable to travel or having conflicting responsibilities making it difficult to leave home (e.g., childcare).

We solicited feedback through a postevent survey. The survey results were used to assist in fine-tuning the event to best suit our residents’ and support persons’ needs. Following our in-person events, we handed out paper surveys and collected the completed ones before participants left the venue. Consistent with what has been found in the survey study literature, we observed that paper surveys had higher response rates than electronic surveys.^24^ A downside to paper surveys is that someone has to manually enter the results into an electronic database. Alternatively, an electronic survey could be utilized by providing onsite participants with a QR code to access the survey, but this might result in a lower response rate.^24^

A limitation in our evaluation of the 2022 event was that we did not specifically assess the knowledge gained pertaining to burnout and substance use disorder. We collected only postevent survey data that probed attendees’ attitudes about the event, perceived learning, and predicted change in communication between residents and support persons at the 2022 event. Since we had evaluated the impact of the event on social support and wellness outcomes in previous years, we prioritized decreasing participant survey burden by administering only a short survey to gauge feedback for future improvement. It is unclear as to whether the event led to changes in social support and wellness among residents in this cohort because we did not collect longitudinal data to systematically assess this outcome. Although we have not detected significant associations between our event and long-term improvements in social support and wellness, such relationships may exist.

The generalizability of our specific program is limited to anesthesiology residency programs, but our resources provide a framework that can be adopted by other specialties. It would be easiest to adapt this event to procedurally based specialties, where anesthesiology-specific hands-on activities could be replaced by other field-specific procedures. However, the event could be utilized for nonprocedurally based areas of medicine where the hands-on portion could be replaced with scenarios such as presenting on rounds, breaking bad news, running a cardiac arrest code, and other scenarios relevant to the specialty. The topics of didactics and panel discussions are relevant to medical education in general. The materials we provide in the appendices could be adapted to address the educational needs of other specialties and support persons of trainees at different stages of their career. For example, our team has successfully adapted and delivered this event to third-year medical students to improve support persons’ understanding of clinical rotations.

In conclusion, our FAX was well received by CA-1 residents and their support persons. We believe that this event can be translated to other anesthesiology residency programs and that our framework can be informative for programs in other medical specialties that are seeking to promote wellness through social support. More work is needed to assess the event's long-term impacts on communication, social support, and wellness. More studies involving multisite programs, larger samples of more diverse learners, and learning environments are warranted to accumulate stronger evidence to determine if the event meets the goals of decreasing resident burnout and wellness mediated by improved social support of residents.

## Appendices


Preevent FAX Checklist.docxSimulation Setup Instructions.docxSchedule of the Day.docxFAX Timeline.docxDay in the Life.mp4Family Day Simulation Scenario.docxHigh-Fidelity Scenario.mp4High-Fidelity Scenario Part 2.mp4Talking Points for Simulation.docxDidactics.pptxPanel Questions and Logistics.docxPostevent Survey.docx
*All appendices are peer reviewed as integral parts of the Original Publication.*

## References

[1] Shanafelt TD, West CP, Sinsky C, et al. Changes in burnout and satisfaction with work-life integration in physicians and the general US working population between 2011 and 2017. Mayo Clin Proc. 2019;94(9):1681–1694. 10.1016/j.mayocp.2018.10.023 30803733

[2] Dyrbye LN, Burke SE, Hardeman RR, et al. Association of clinical specialty with symptoms of burnout and career choice regret among US resident physicians. JAMA. 2018;320(11):1114–1130. 10.1001/jama.2018.12615 30422299 PMC6233627

[3] Rodrigues H, Cobucci R, Oliveira A, et al. Burnout syndrome among medical residents: a systematic review and meta-analysis. PLoS One. 2018;13(11):e0206840. 10.1371/journal.pone.0206840 30418984 PMC6231624

[4] de Oliveira GS Jr, Chang R, Fitzgerald PC, et al. The prevalence of burnout and depression and their association with adherence to safety and practice standards: a survey of United States anesthesiology trainees. Anesth Analg. 2013;117(1):182–193. 10.1213/ANE.0b013e3182917da9 23687232

[5] Sun H, Warner DO, Macario A, Zhou Y, Culley DJ, Keegan MT. Repeated cross-sectional surveys of burnout, distress, and depression among anesthesiology residents and first-year graduates. Anesthesiology. 2019;131(3):668–677. 10.1097/ALN.0000000000002777 31166235

[6] Shanafelt T, Goh J, Sinsky C. The business case for investing in physician well-being. JAMA Intern Med. 2017;177(12):1826–1832. 10.1001/jamainternmed.2017.4340 28973070

[7] West CP, Huschka MM, Novotny PJ, et al. Association of perceived medical errors with resident distress and empathy: a prospective longitudinal study. JAMA. 2006;296(9):1071–1078. 10.1001/jama.296.9.1071 16954486

[8] Fahrenkopf AM, Sectish TC, Barger LK, et al. Rates of medication errors among depressed and burnt out residents: prospective cohort study. BMJ. 2008;336(7642):488. 10.1136/bmj.39469.763218.BE 18258931 PMC2258399

[9] Shanafelt TD, Balch CM, Bechamps G, et al. Burnout and medical errors among American surgeons. Ann Surg. 2010;251(6):995–1000. 10.1097/SLA.0b013e3181bfdab3 19934755

[10] Warner DO, Berge K, Sun H, Harman A, Hanson A, Schroeder DR. Substance use disorder among anesthesiology residents, 1975–2009. JAMA. 2013;310(21):2289–2296. 10.1001/jama.2013.281954 24302092 PMC3993973

[11] Raj KS. Well-being in residency: a systematic review. J Grad Med Educ. 2016;8(5):674–684. 10.4300/JGME-D-15-00764.1 28018531 PMC5180521

[12] Rappaport WD, Putnam CW, Witzke D, Amil B. Helping residents’ families cope. Acad Med. 1992;67(11):761. 10.1097/00001888-199211000-00009 1418254

[13] Wainwright E, Looseley A, Mouton R, et al.; SWeAT Study Investigator Group. Stress, burnout, depression and work satisfaction among UK anaesthetic trainees: a qualitative analysis of in-depth participant interviews in the Satisfaction and Wellbeing in Anaesthetic Training study. Anaesthesia. 2019;74(10):1240–1251. 10.1111/anae.14694 31090927

[14] Law M, Lam M, Wu D, Veinot P, Mylopoulos M. Changes in personal relationships during residency and their effects on resident wellness: a qualitative study. Acad Med. 2017;92(11):1601–1606. 10.1097/ACM.0000000000001711 28445221 PMC5662155

[15] ACGME Common Program Requirements (Residency). Accreditation Council for Graduate Medical Education; 2021. Accessed November 2, 2023. https://www.acgme.org/globalassets/pfassets/programrequirements/cprresidency_2022v3.pdf

[16] Gottschalk A, Seelen S, Tivey S, Gottschalk A, Rich G. What do patients know about anesthesiologists? Results of a comparative survey in an U.S., Australian, and German university hospital. J Clin Anesth. 2013;25(2):85–91. 10.1016/j.jclinane.2012.06.017 23333789

[17] Sloan SJ, Cooper CL. Stress coping strategies in commercial airline pilots. J Occup Med. 1986;28(1):49–52. 10.1097/00043764-198601000-00013 3950782

[18] Karlins M, Koh F, McCully L. The spousal factor in pilot stress. Aviat Space Environ Med. 1989;60(11):1112–1115. 2684130

[19] Aggarwal R, Deutsch JK, Medina J, Kothari N. Resident wellness: an intervention to decrease burnout and increase resiliency and happiness. MedEdPORTAL. 2017;13:10651. 10.15766/mep_2374-8265.10651 30800852 PMC6338253

[20] Donaghy R, Tomatsu S, Kerns P, White C, Ratliff J. An educational workshop to improve neurology resident understanding of burnout, substance abuse, and mood disorders. MedEdPORTAL. 2021;17:11164. 10.15766/mep_2374-8265.11164 34277931 PMC8245593

[21] Chen F, Rahim S, Isaak R, et al. Maintaining social support in the era of social distancing: transitioning an in-person family-oriented wellness event to a virtual venue. eLearn Magazine. 2023;2023(2):4. 10.1145/3583062.3539612

[22] Martinelli SM, Chen F, Hobbs G, et al. The use of simulation to improve family understanding and support of anesthesia providers. Cureus. 2018;10(3):e2262. 10.7759/cureus.2262 29725565 PMC5931409

[23] Martinelli SM, Isaak RS, Chidgey BA, et al. Family comes first: a pilot study of the incorporation of social support into resident well-being. J Educ Perioper Med. 2020;22(4):E652. 10.46374/volxxii-issue4-martinelli 33447651 PMC7792563

[24] Blumenberg C, Barros AJD. Response rate differences between web and alternative data collection methods for public health research: a systematic review of the literature. Int J Public Health. 2018;63(6):765–773. 10.1007/s00038-018-1108-4 29691594

