# Supplementary material for: Family Anesthesia Experience: Improving Social Support of Residents Through Education of Their Family and Friends
Source: MedEdPORTAL. 2023 Dec 15;19:11370. doi: 10.15766/mep_2374-8265.11370 (PMC10721742; doi:10.15766/mep_2374-8265.11370)
Supplement: Supplementary file 1 — Preevent FAX Checklist.docxSimulation Setup Instructions.docxSchedule of the Day.docxFAX Timeline.docxDay in the Life.mp4Family Day Simulation Scenario.docxHigh-Fidelity Scenario.mp4High-Fidelity Scenario Part 2.mp4Talking Points for Simulation.docxDidactics.pptxPanel Questions and Logistics.docxPostevent Survey.docx [file mep_2374-8265.11370-s001.zip › I. Talking Points for Simulation.docx]

**Talking points for demonstrations**

This document provides talking points that can be used during the hands-on stations. The talking points are geared primarily for the CA-1’s support persons. However, if the CA-1 residents have questions or comments they should also be addressed.

**Airway management**

1. Difficult airway algorithm, backup plans
2. Faculty always present during airway management
3. Airway exam, predictors of difficult airway
4. How it feels after unanticipated difficult airway

**Procedures/task trainers**

1. Describe training for each procedure
2. When each procedure might be utilized

**OR experience**

1. How many times have you had an emergency like this in the operating room?
2. How soon into your residency did you experience an emergency?
3. How do you know when to call for help?
4. What’s the difference between a resident and an attending?
5. What’s the difference between a resident and a CRNA?
6. When an emergency happens, who is the leader (surgeon, resident, anesthesiology attending) and how does the team work together?
