# Supplementary material for: Family Anesthesia Experience: Improving Social Support of Residents Through Education of Their Family and Friends
Source: MedEdPORTAL. 2023 Dec 15;19:11370. doi: 10.15766/mep_2374-8265.11370 (PMC10721742; doi:10.15766/mep_2374-8265.11370)
Supplement: Supplementary file 1 — Preevent FAX Checklist.docxSimulation Setup Instructions.docxSchedule of the Day.docxFAX Timeline.docxDay in the Life.mp4Family Day Simulation Scenario.docxHigh-Fidelity Scenario.mp4High-Fidelity Scenario Part 2.mp4Talking Points for Simulation.docxDidactics.pptxPanel Questions and Logistics.docxPostevent Survey.docx [file mep_2374-8265.11370-s001.zip › J. Didactics.pptx]

## Slide 1
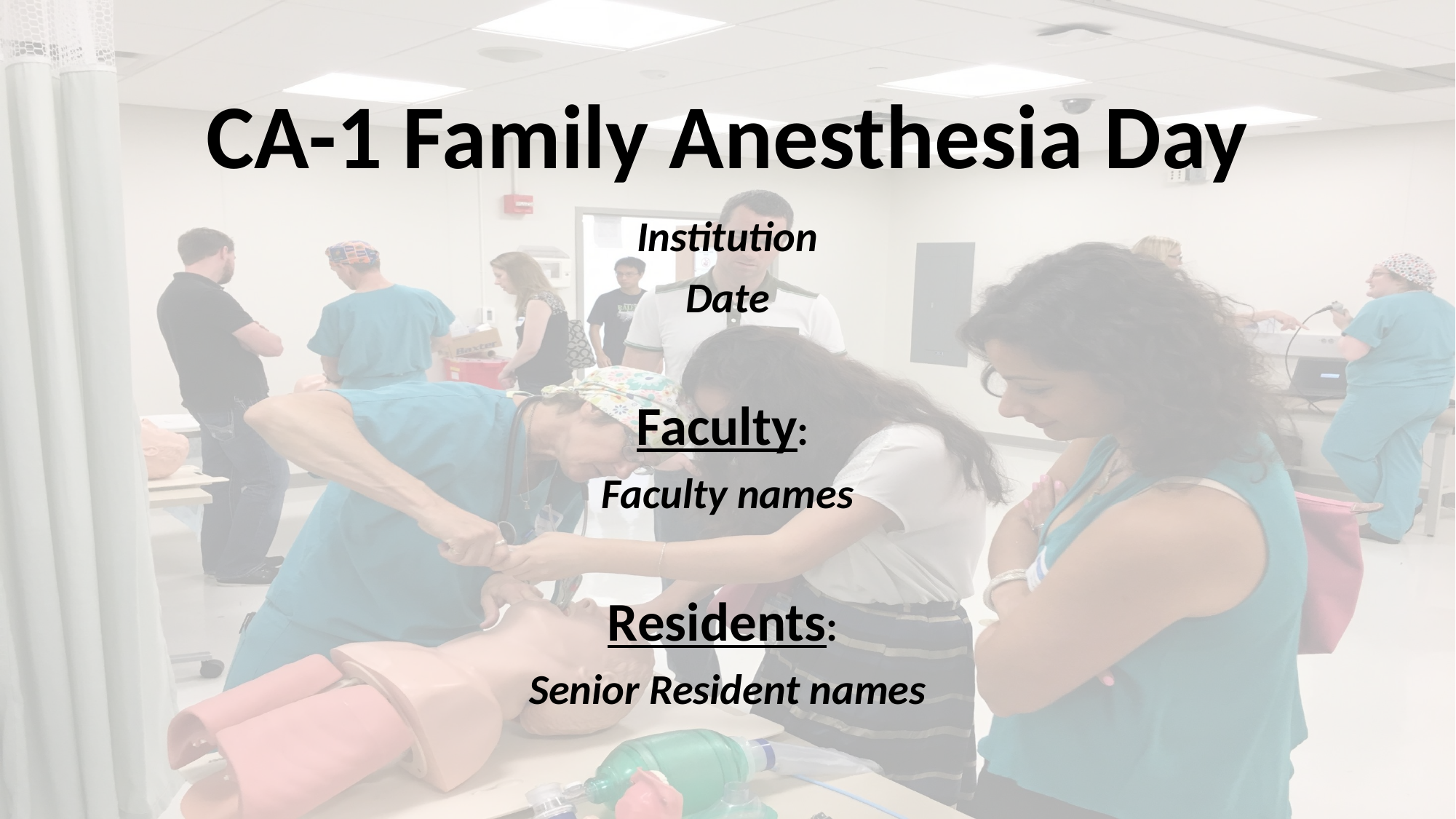

# CA-1 Family Anesthesia Day
Institution
Date
Faculty:
Faculty names
Residents:
Senior Resident names

## Slide 2
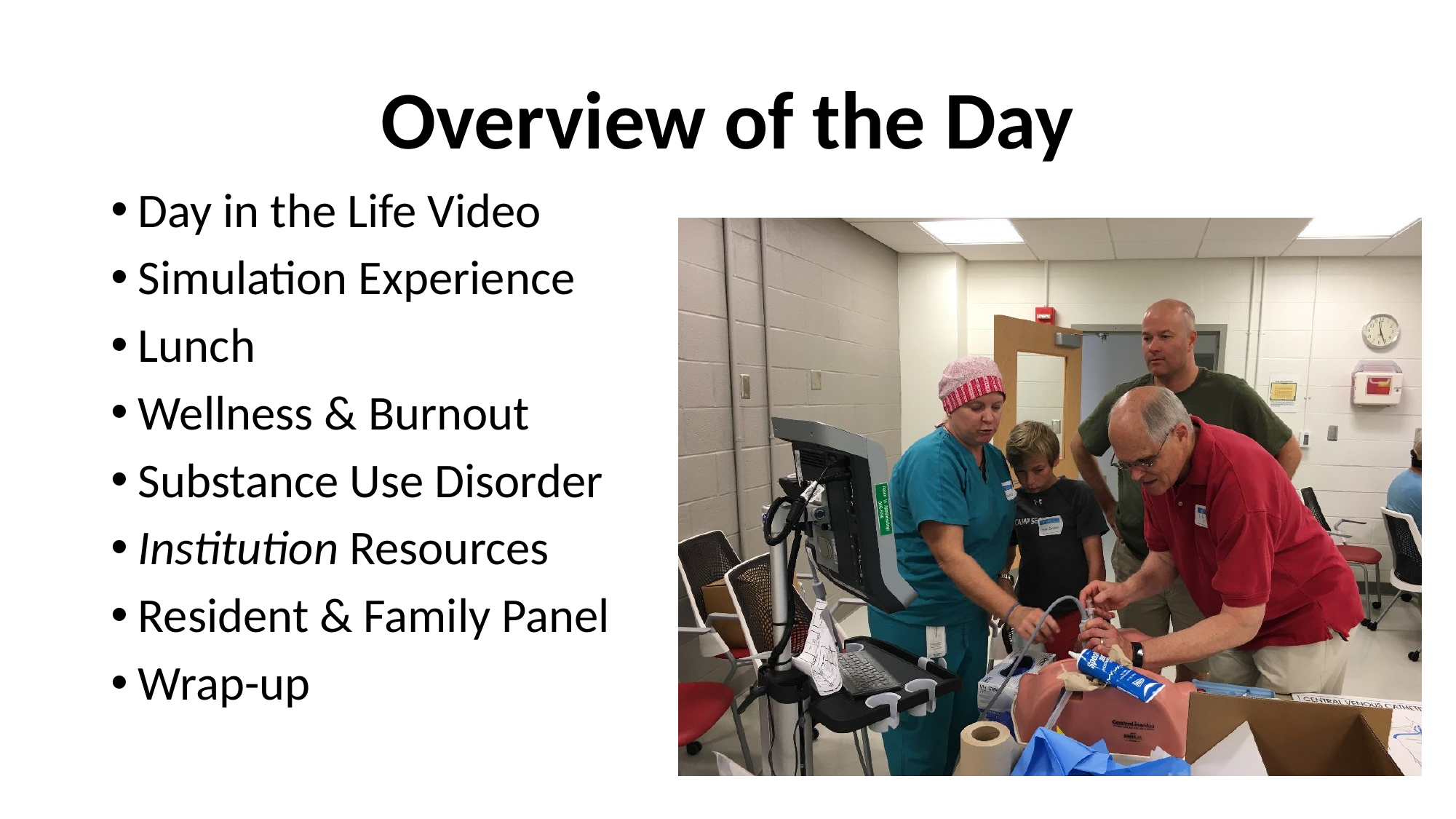

# Overview of the Day
Day in the Life Video
Simulation Experience
Lunch
Wellness & Burnout
Substance Use Disorder
Institution Resources
Resident & Family Panel
Wrap-up

## Slide 3
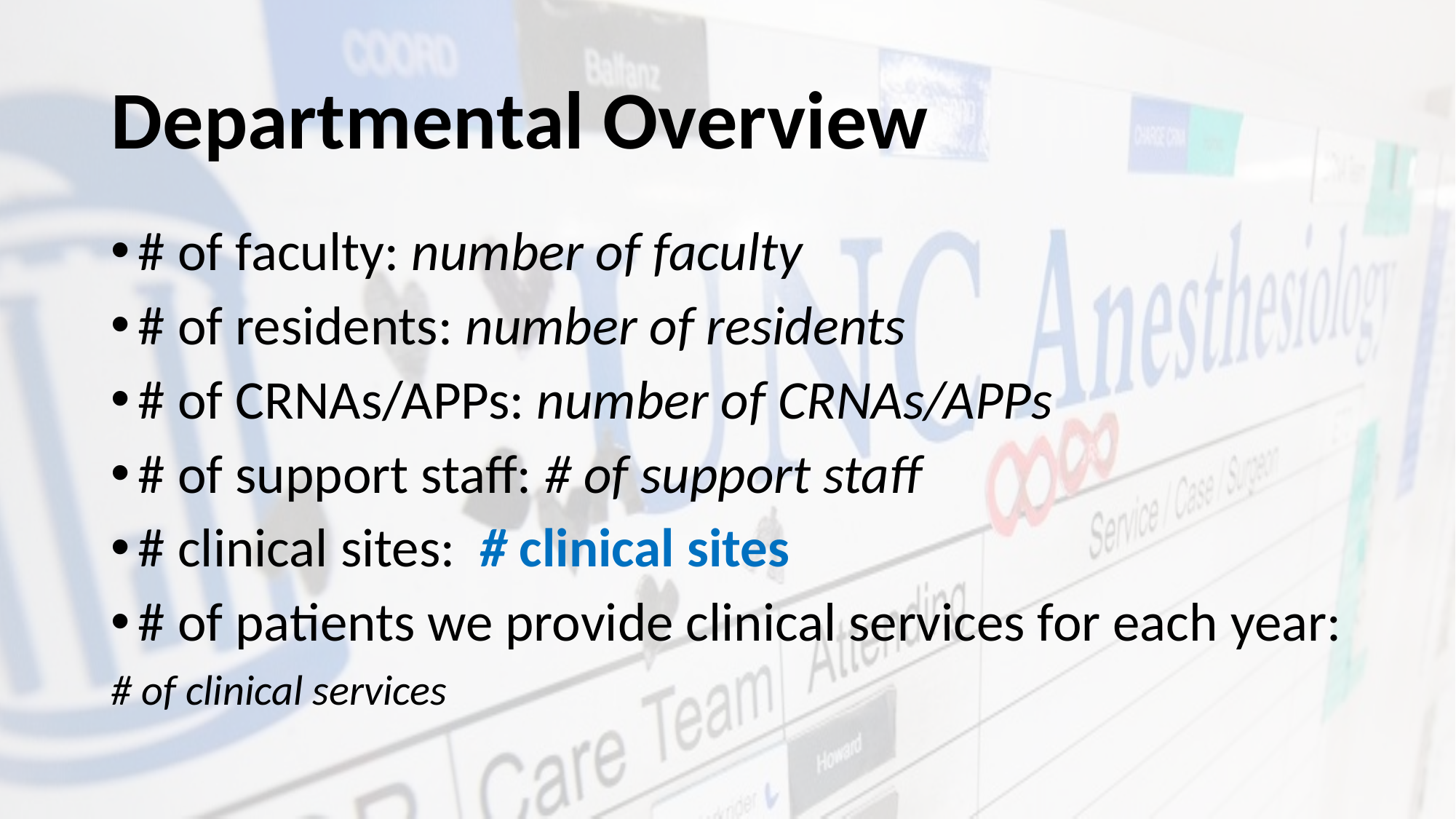

# Departmental Overview
# of faculty: number of faculty
# of residents: number of residents
# of CRNAs/APPs: number of CRNAs/APPs
# of support staff: # of support staff
# clinical sites: # clinical sites
# of patients we provide clinical services for each year:
# of clinical services

## Slide 4
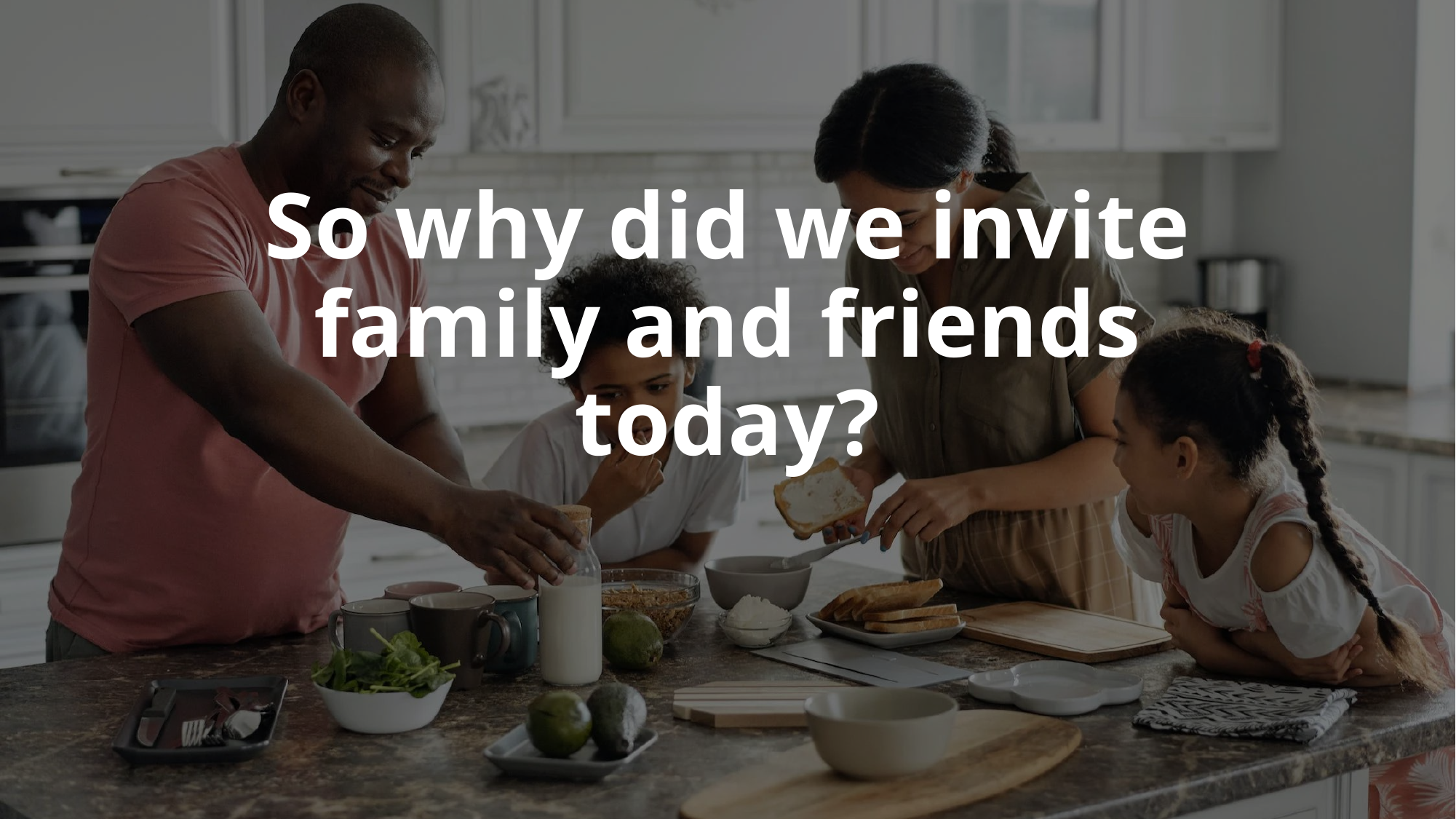

# So why did we invite family and friends today?

## Slide 5
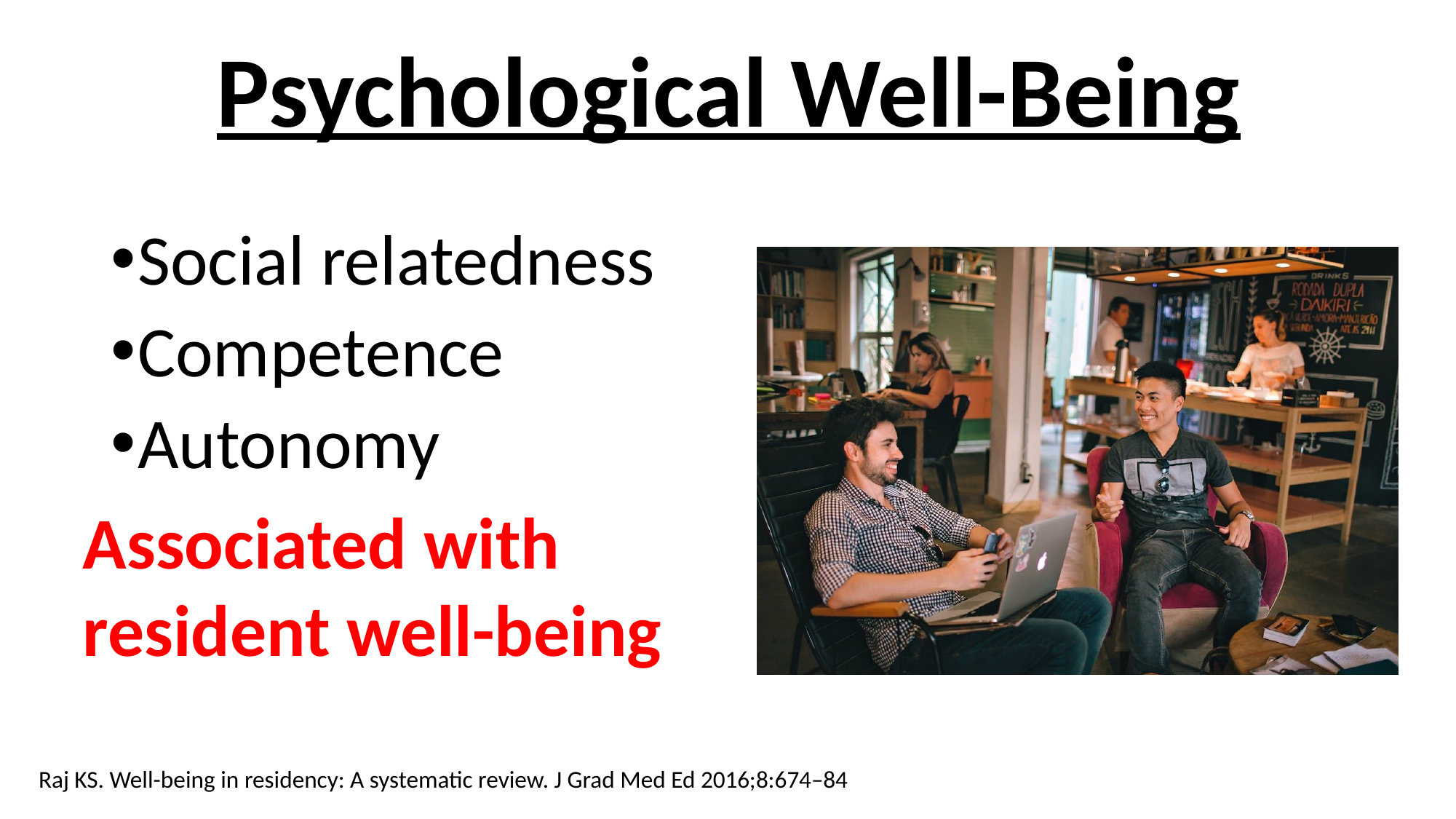

# Psychological Well-Being
Social relatedness
Competence
Autonomy
Associated with resident well-being
Raj KS. Well-being in residency: A systematic review. J Grad Med Ed 2016;8:674–84

## Slide 6
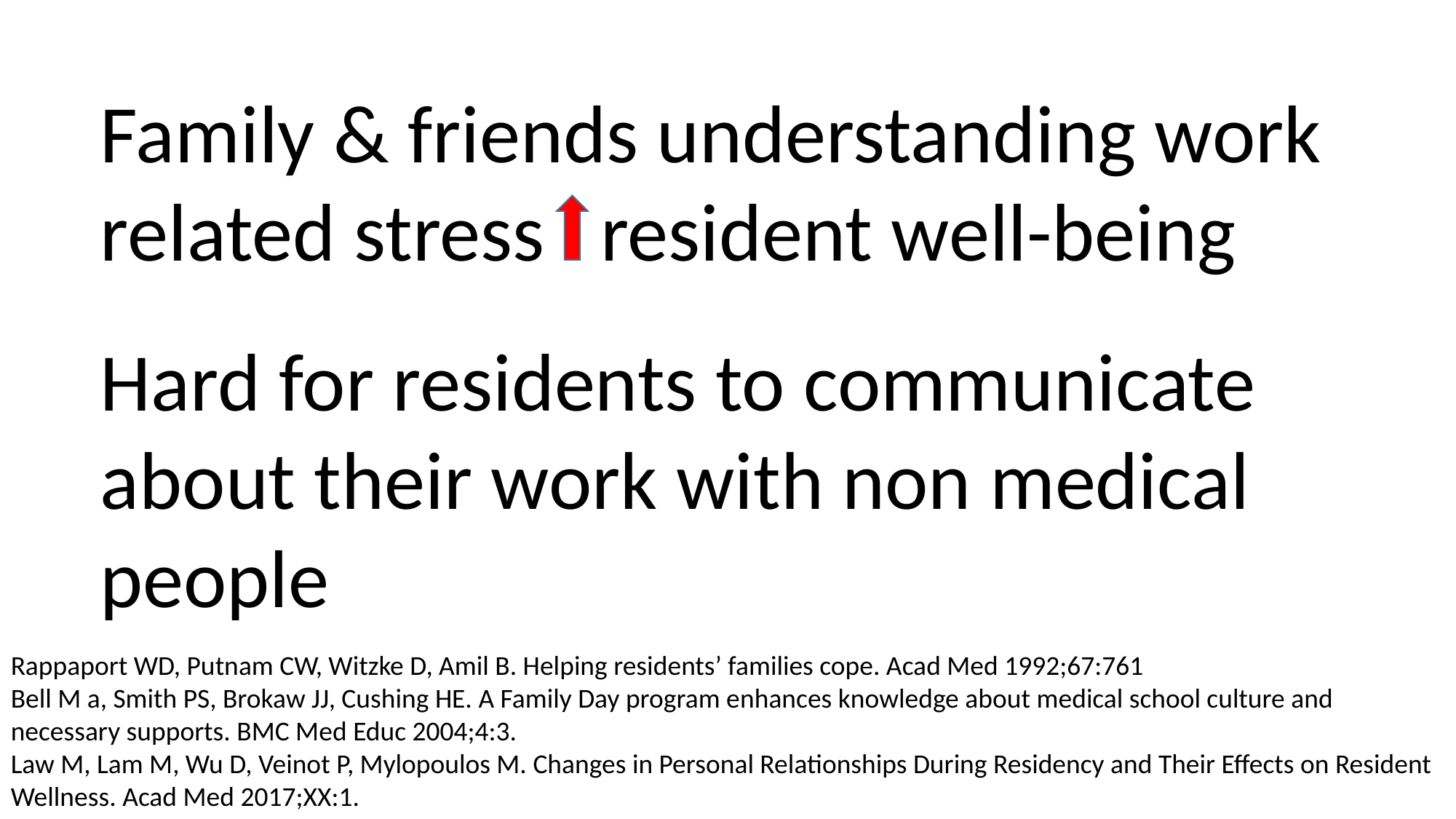

Family & friends understanding work related stress resident well-being
Hard for residents to communicate about their work with non medical people
Rappaport WD, Putnam CW, Witzke D, Amil B. Helping residents’ families cope. Acad Med 1992;67:761
Bell M a, Smith PS, Brokaw JJ, Cushing HE. A Family Day program enhances knowledge about medical school culture and necessary supports. BMC Med Educ 2004;4:3.
Law M, Lam M, Wu D, Veinot P, Mylopoulos M. Changes in Personal Relationships During Residency and Their Effects on Resident Wellness. Acad Med 2017;XX:1.

## Slide 7
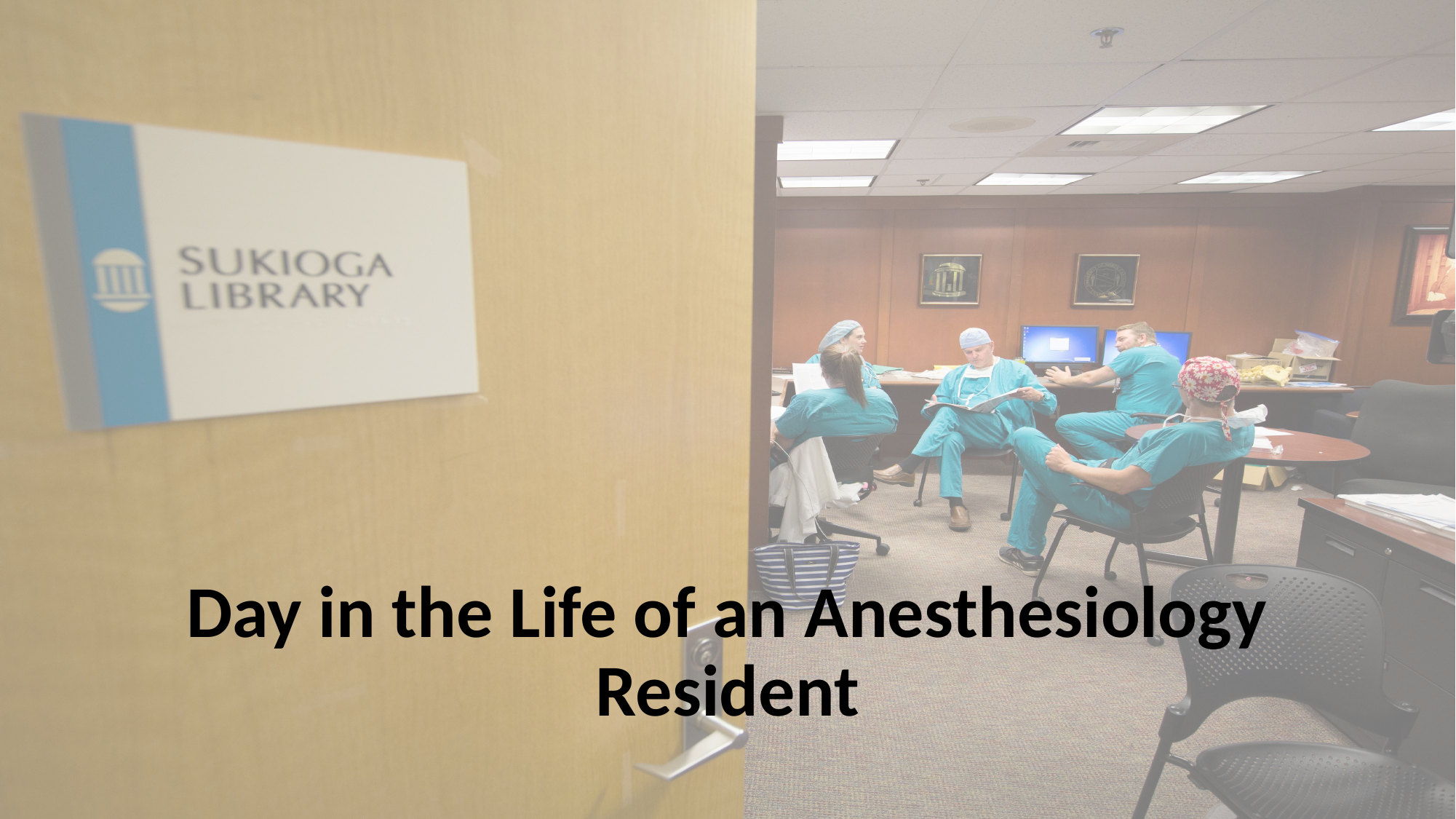

# Day in the Life of an Anesthesiology Resident

## Slide 8
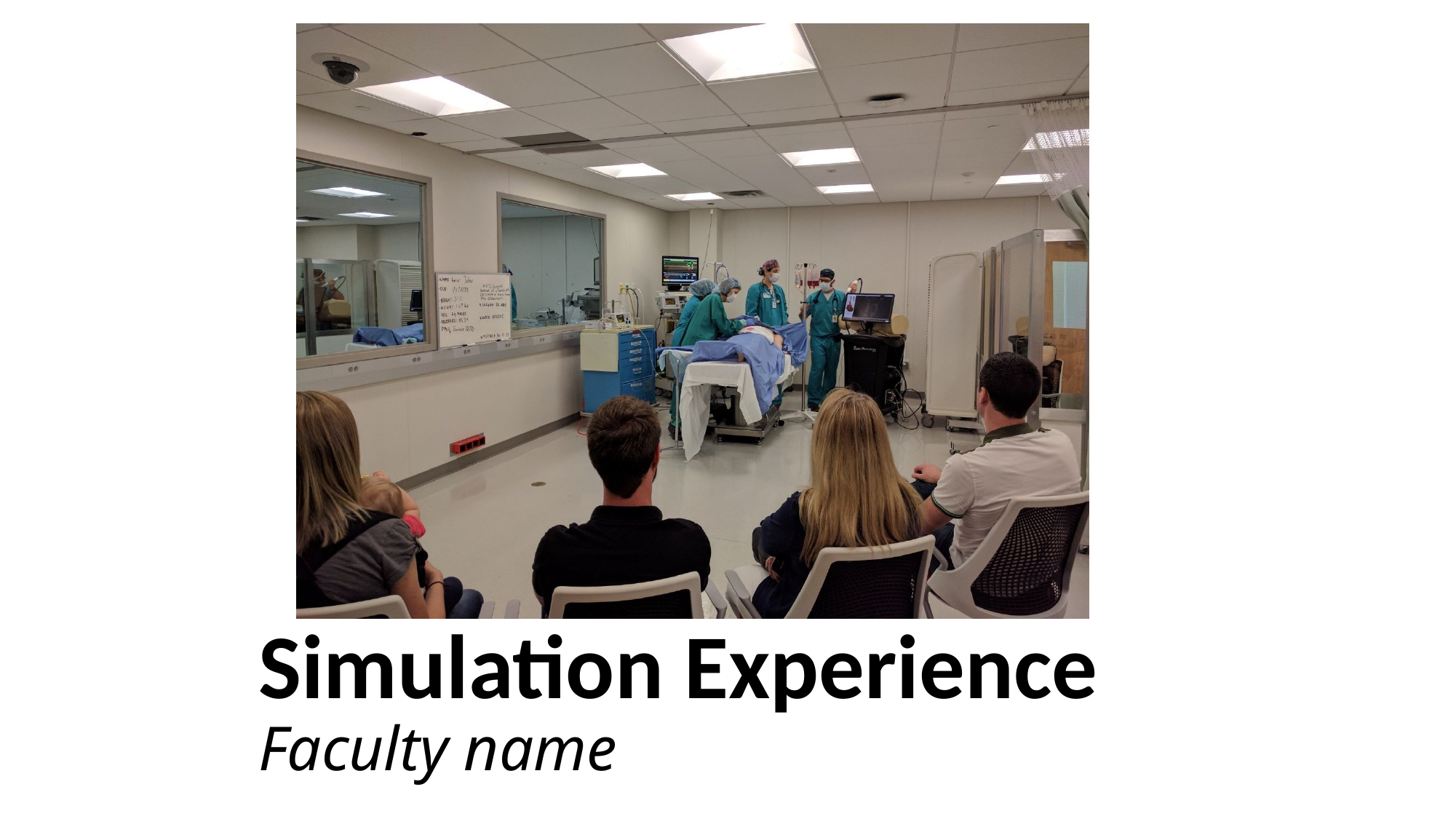

# Simulation Experience Faculty name

## Slide 9
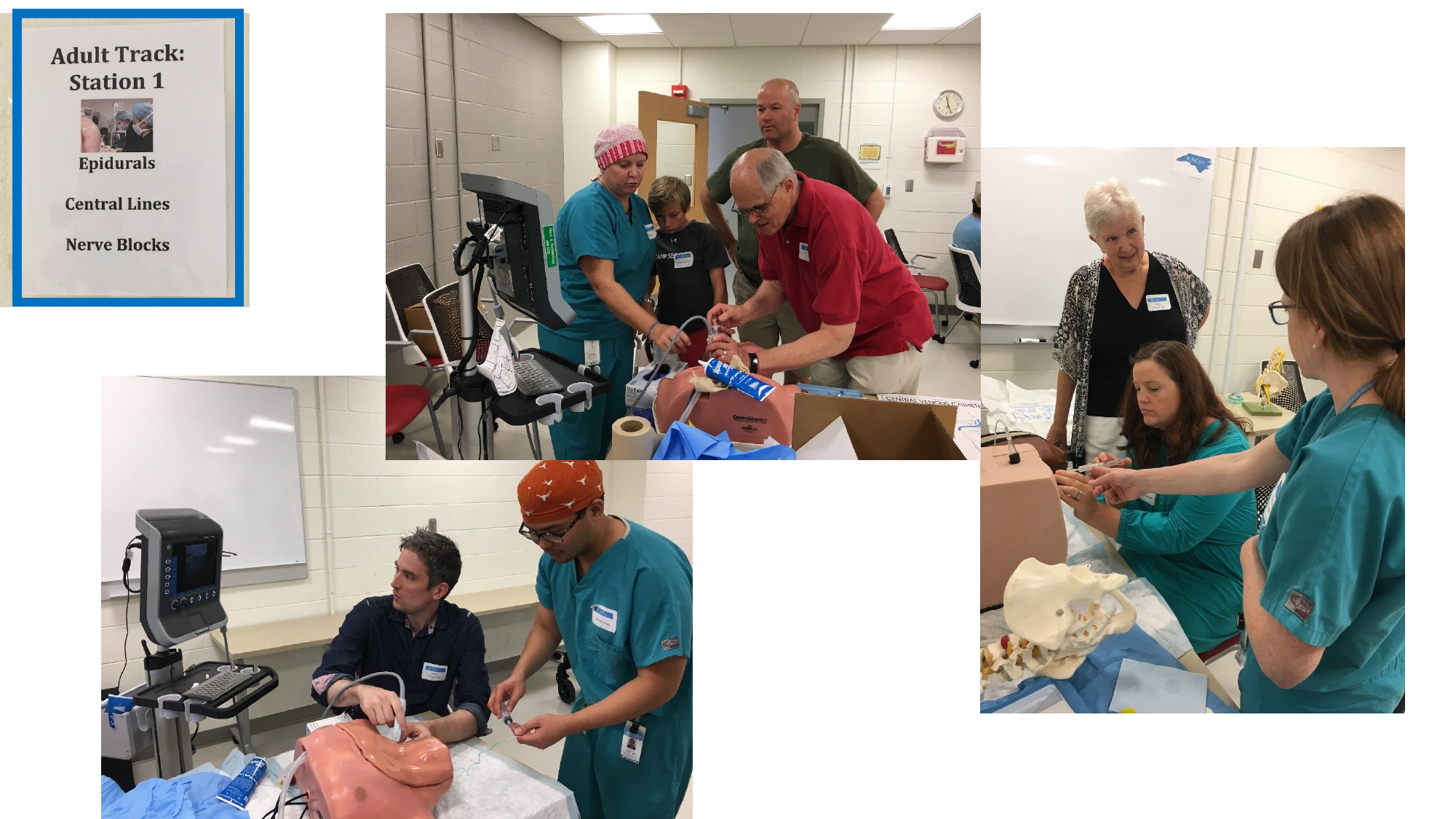

## Slide 10
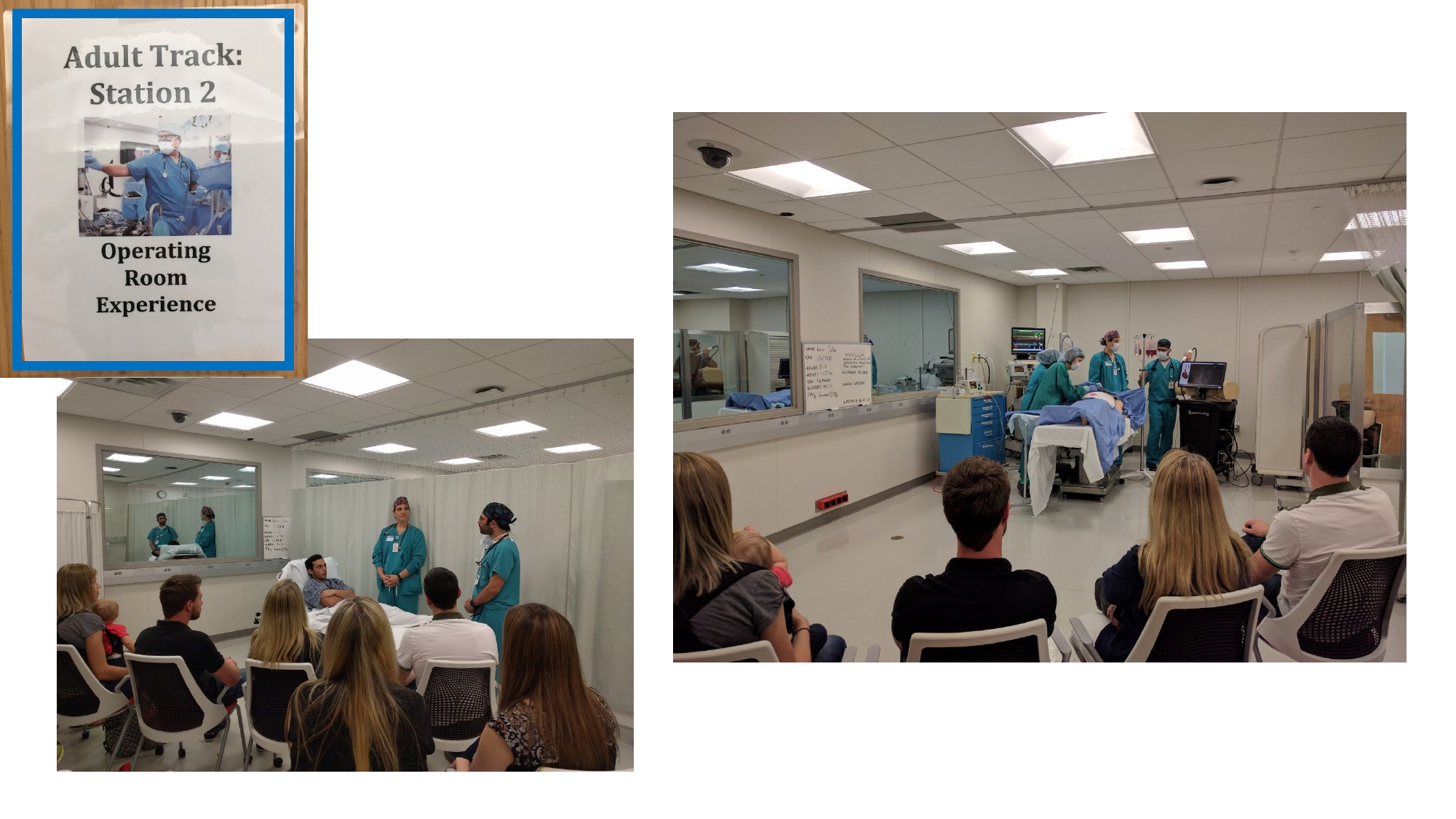

## Slide 11
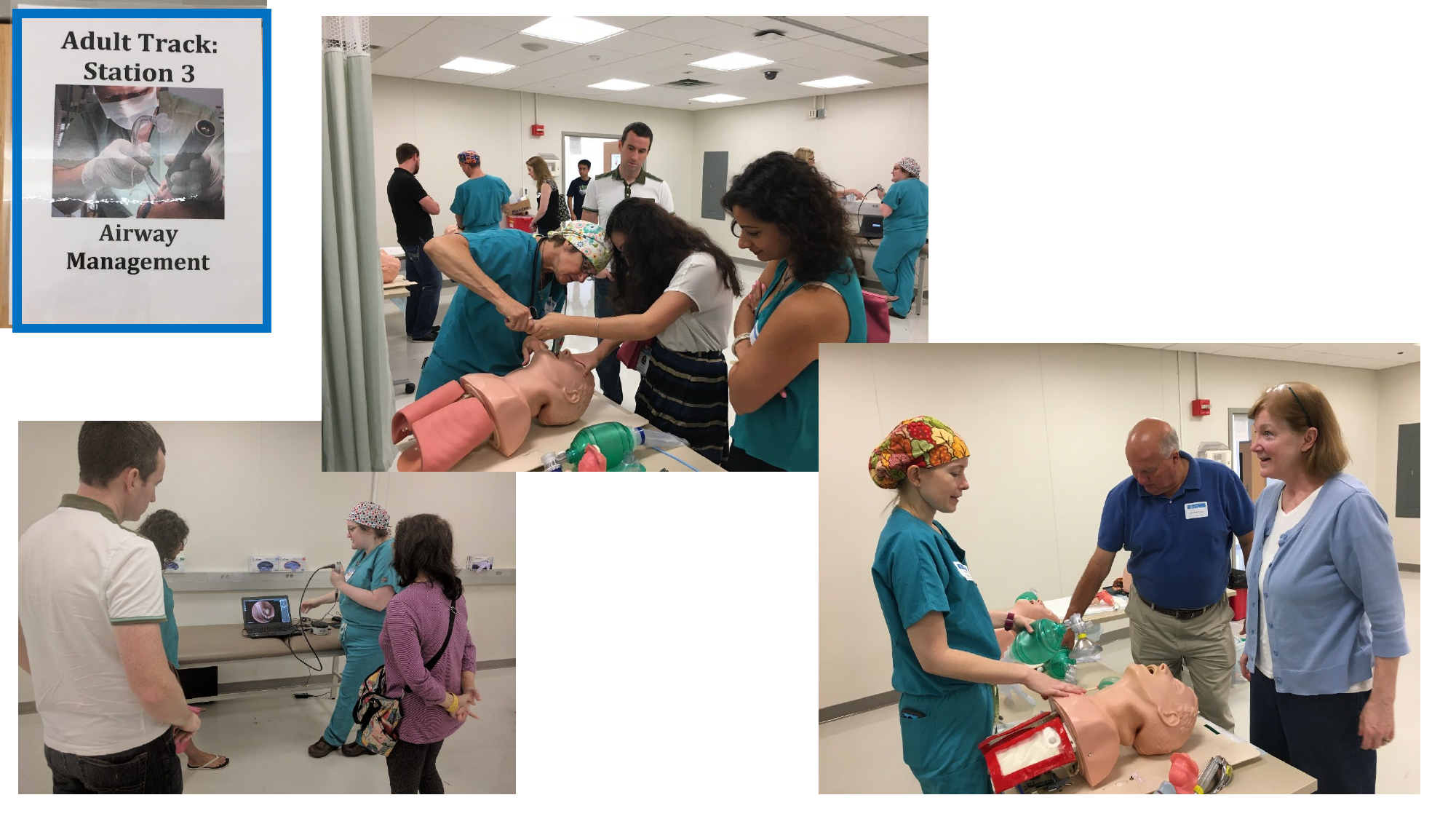

## Slide 12
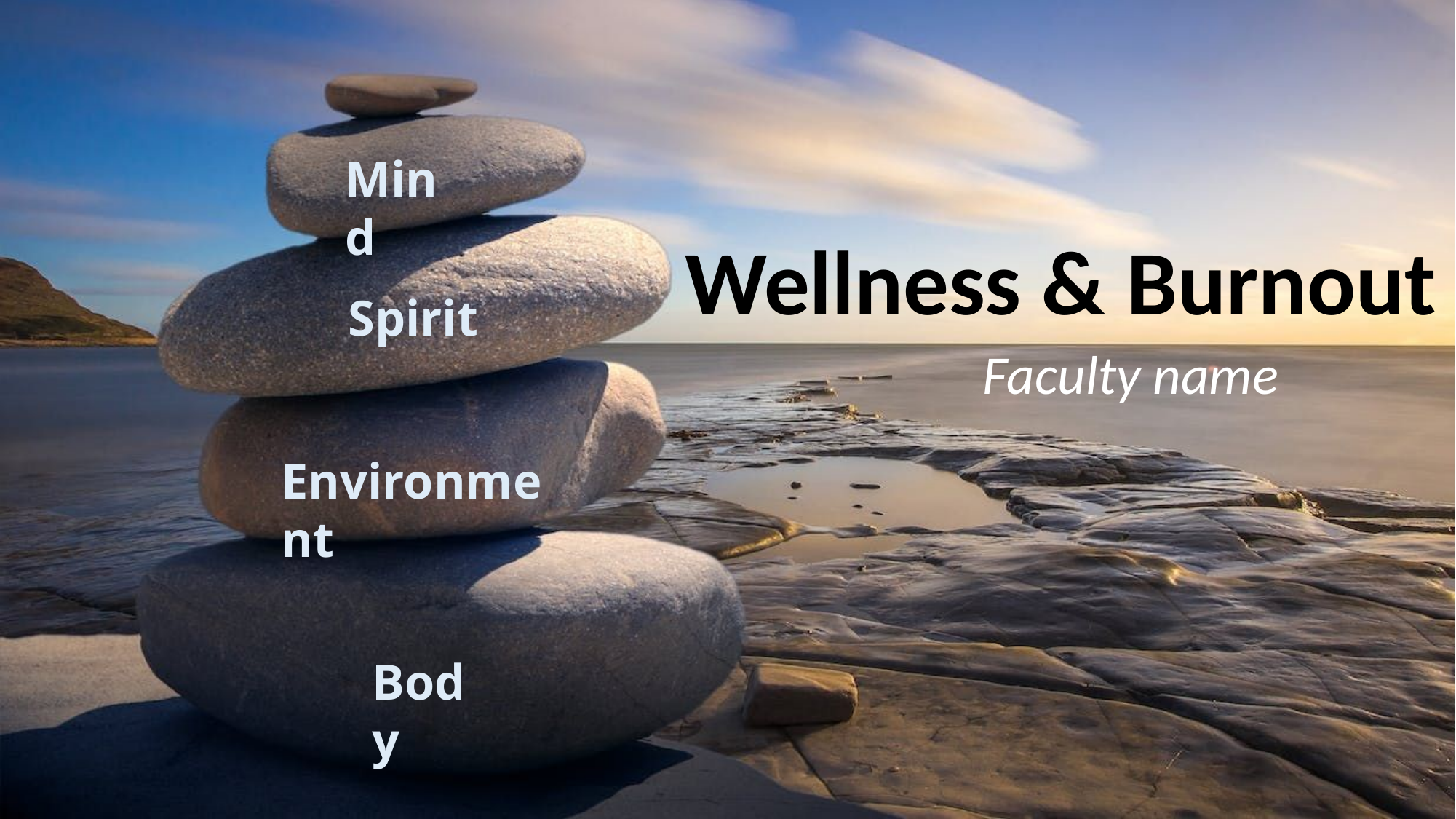

# Wellness & Burnout
Mind
Spirit
Faculty name
Environment
Body

## Slide 13
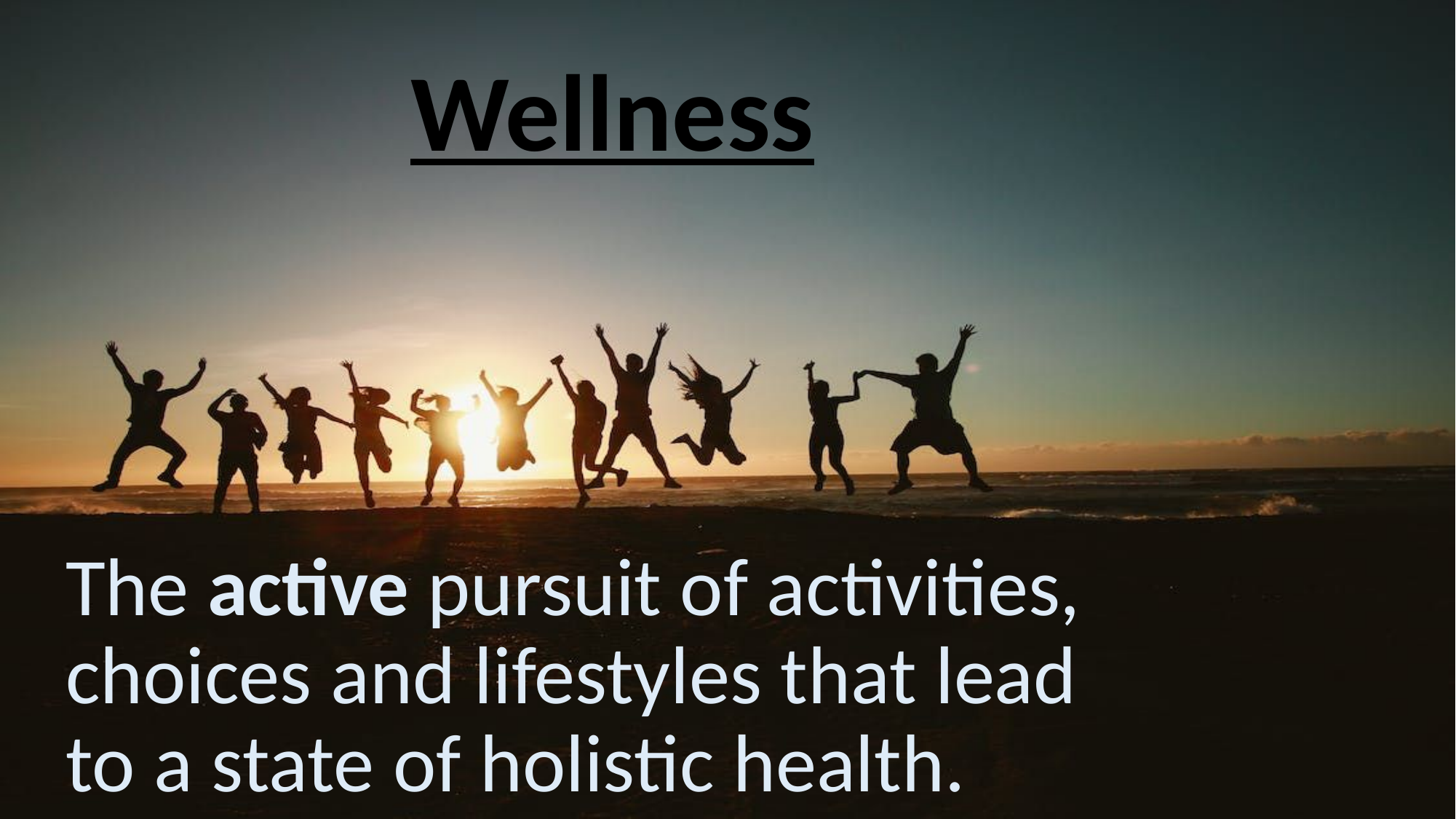

Wellness
The active pursuit of activities, choices and lifestyles that lead to a state of holistic health.

## Slide 14
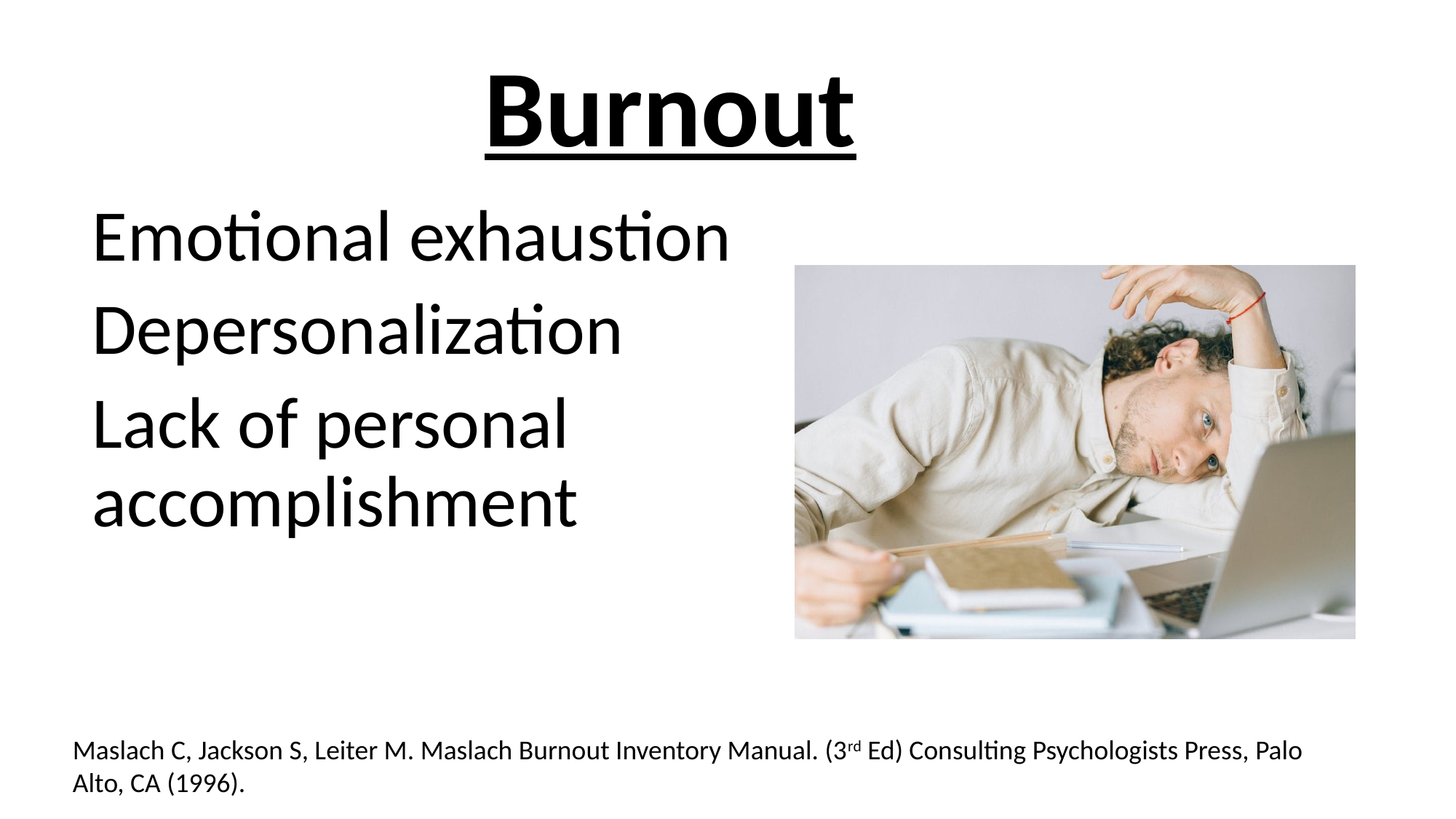

Burnout
Emotional exhaustion
Depersonalization
Lack of personal accomplishment
Maslach C, Jackson S, Leiter M. Maslach Burnout Inventory Manual. (3rd Ed) Consulting Psychologists Press, Palo Alto, CA (1996).

## Slide 15
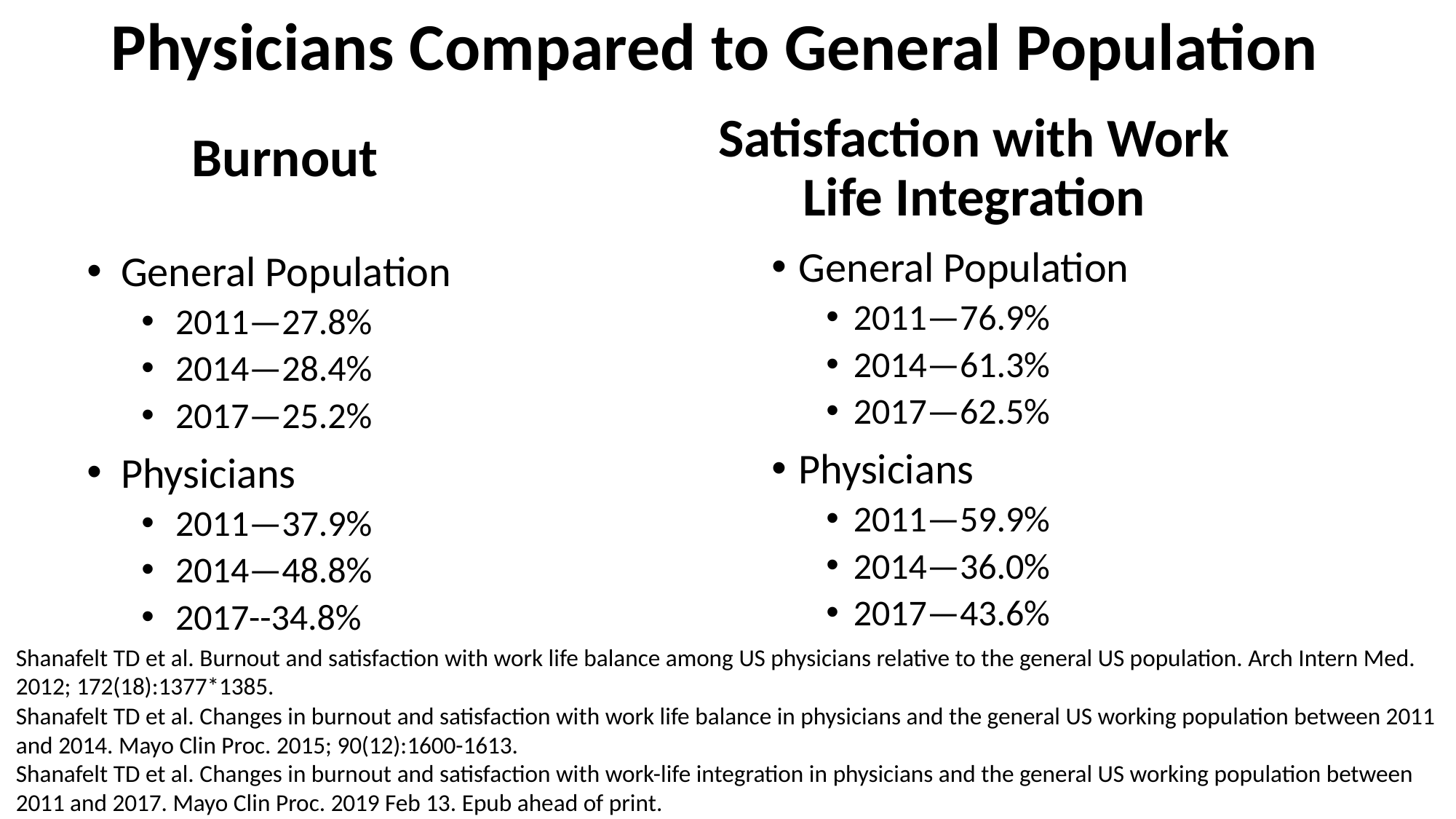

# Physicians Compared to General Population
Burnout
Satisfaction with Work Life Integration
General Population
2011—76.9%
2014—61.3%
2017—62.5%
Physicians
2011—59.9%
2014—36.0%
2017—43.6%
General Population
2011—27.8%
2014—28.4%
2017—25.2%
Physicians
2011—37.9%
2014—48.8%
2017--34.8%
Shanafelt TD et al. Burnout and satisfaction with work life balance among US physicians relative to the general US population. Arch Intern Med. 2012; 172(18):1377*1385.
Shanafelt TD et al. Changes in burnout and satisfaction with work life balance in physicians and the general US working population between 2011 and 2014. Mayo Clin Proc. 2015; 90(12):1600-1613.
Shanafelt TD et al. Changes in burnout and satisfaction with work-life integration in physicians and the general US working population between 2011 and 2017. Mayo Clin Proc. 2019 Feb 13. Epub ahead of print.

## Slide 16
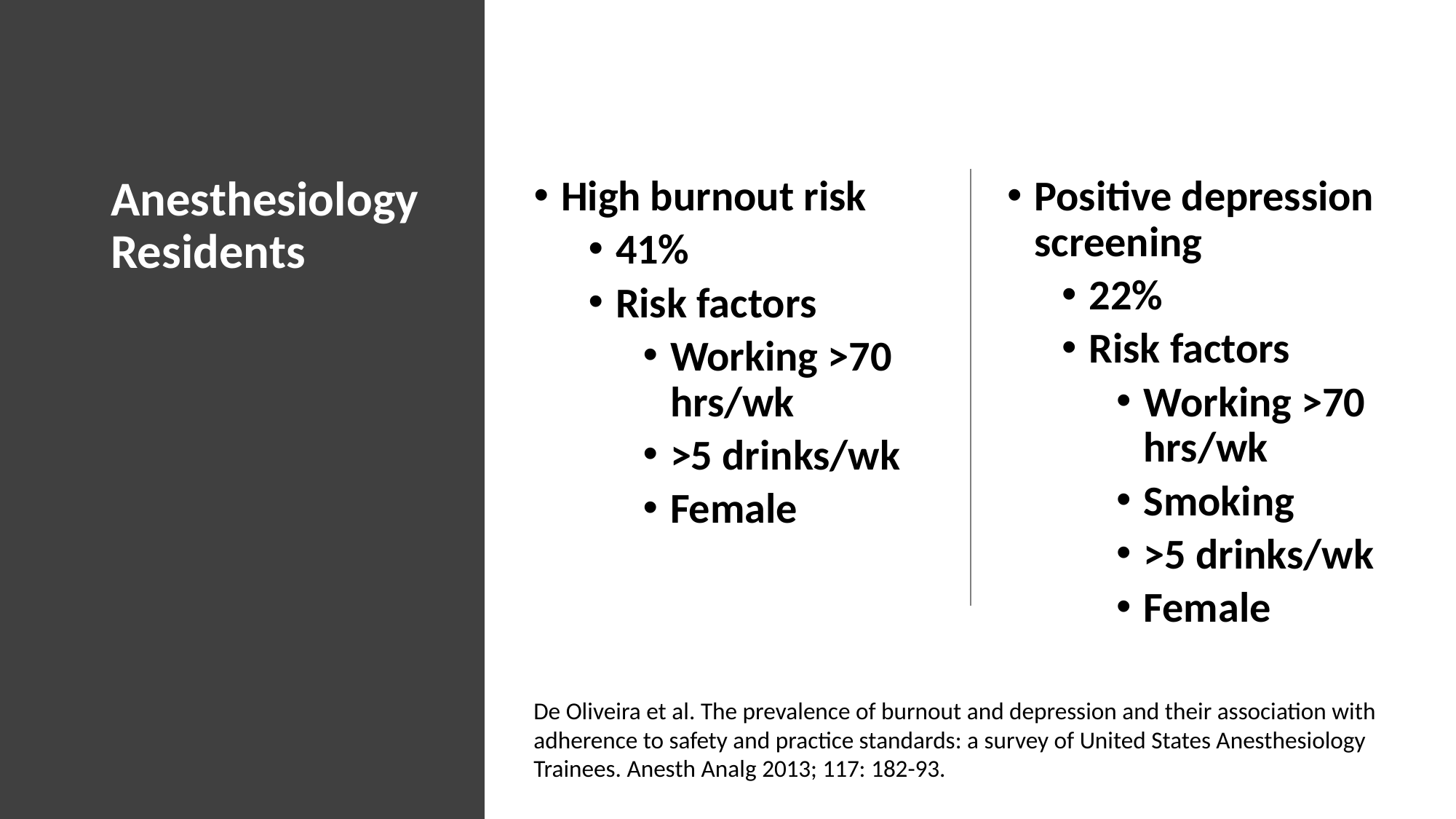

# Anesthesiology Residents
High burnout risk
41%
Risk factors
Working >70 hrs/wk
>5 drinks/wk
Female
Positive depression screening
22%
Risk factors
Working >70 hrs/wk
Smoking
>5 drinks/wk
Female
De Oliveira et al. The prevalence of burnout and depression and their association with adherence to safety and practice standards: a survey of United States Anesthesiology Trainees. Anesth Analg 2013; 117: 182-93.

## Slide 17
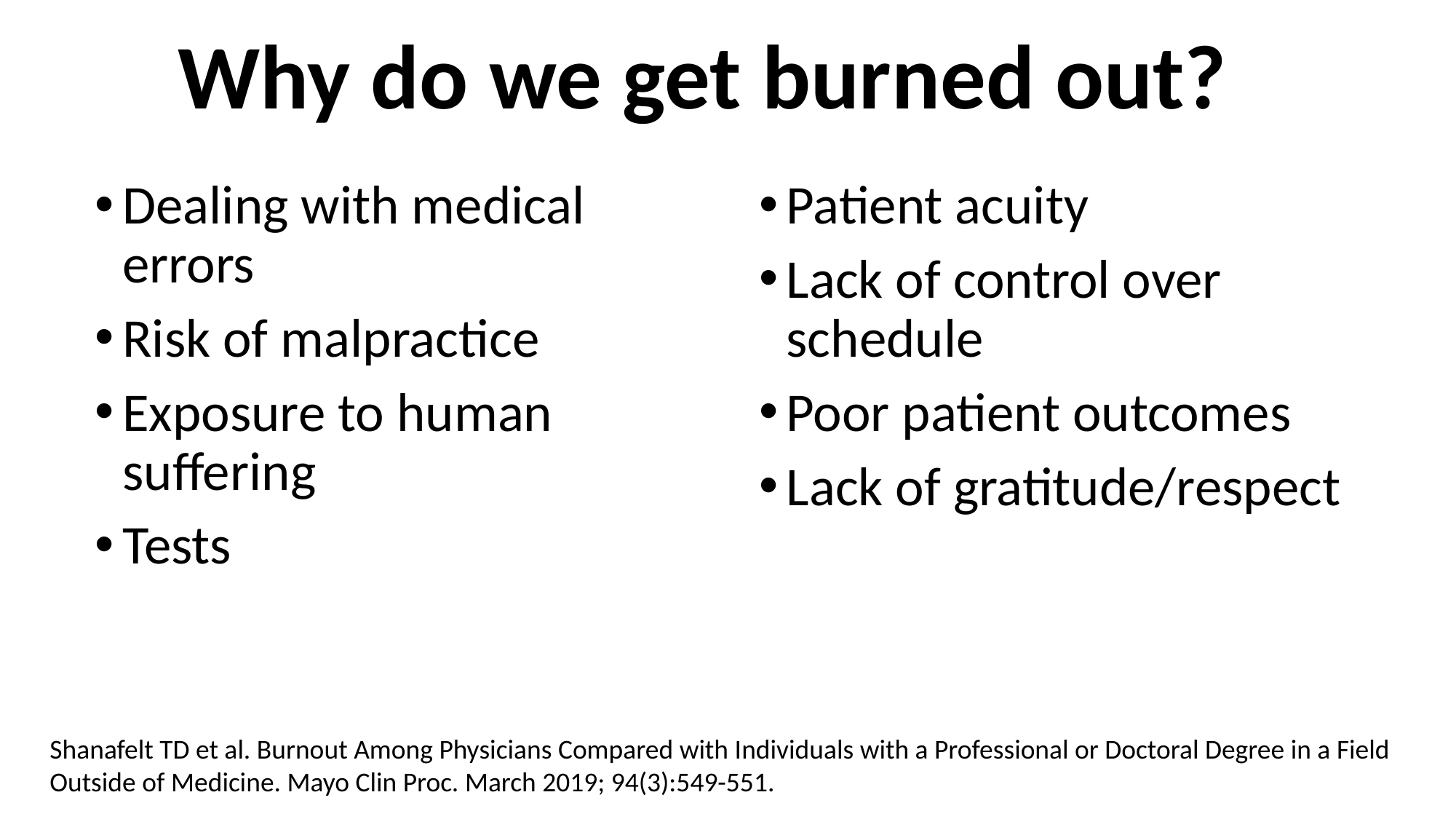

# Why do we get burned out?
Dealing with medical errors
Risk of malpractice
Exposure to human suffering
Tests
Patient acuity
Lack of control over schedule
Poor patient outcomes
Lack of gratitude/respect
Shanafelt TD et al. Burnout Among Physicians Compared with Individuals with a Professional or Doctoral Degree in a Field Outside of Medicine. Mayo Clin Proc. March 2019; 94(3):549-551.

## Slide 18
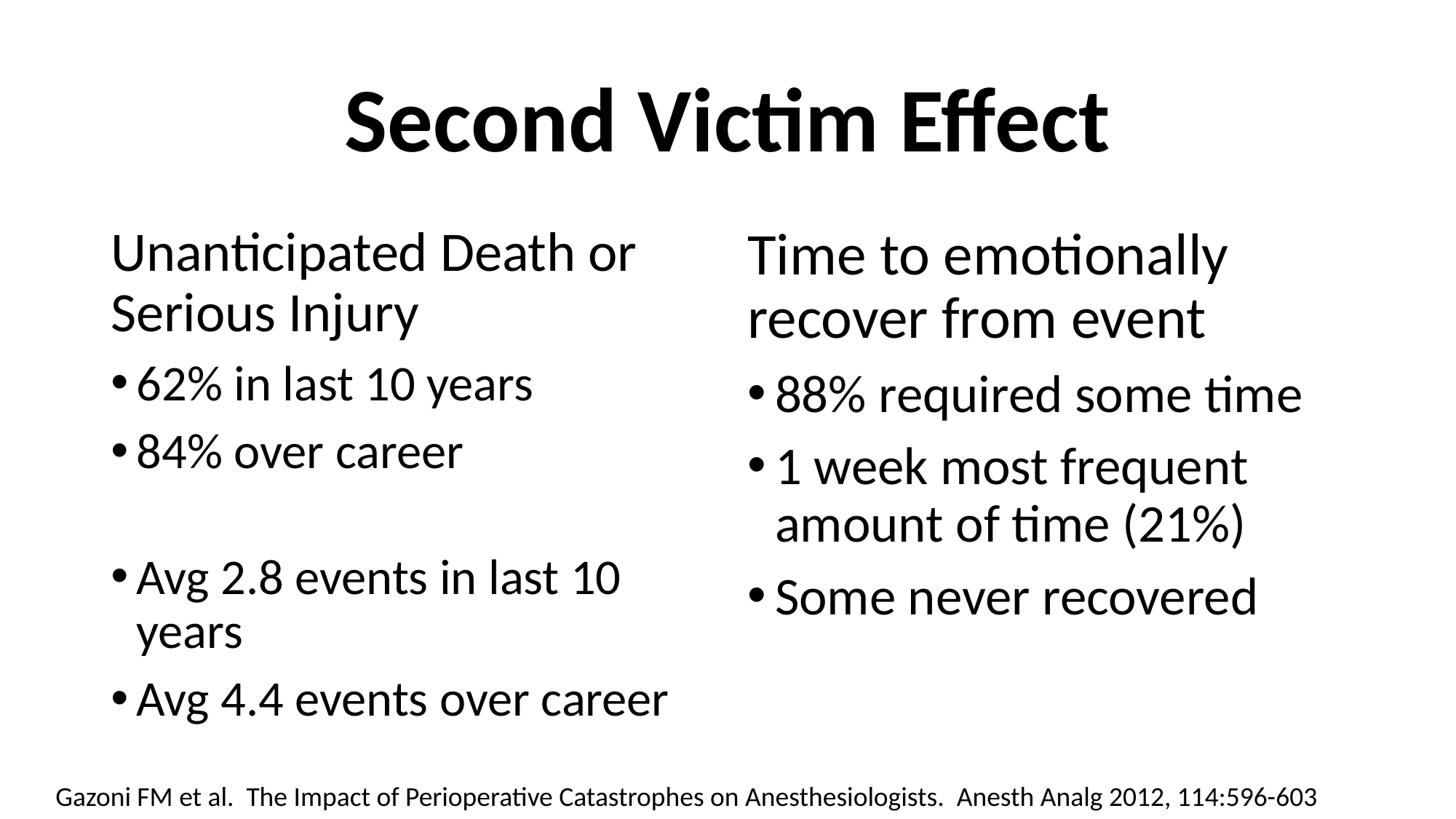

# Second Victim Effect
Unanticipated Death or Serious Injury
62% in last 10 years
84% over career
Avg 2.8 events in last 10 years
Avg 4.4 events over career
Time to emotionally recover from event
88% required some time
1 week most frequent amount of time (21%)
Some never recovered
Gazoni FM et al. The Impact of Perioperative Catastrophes on Anesthesiologists. Anesth Analg 2012, 114:596-603

## Slide 19
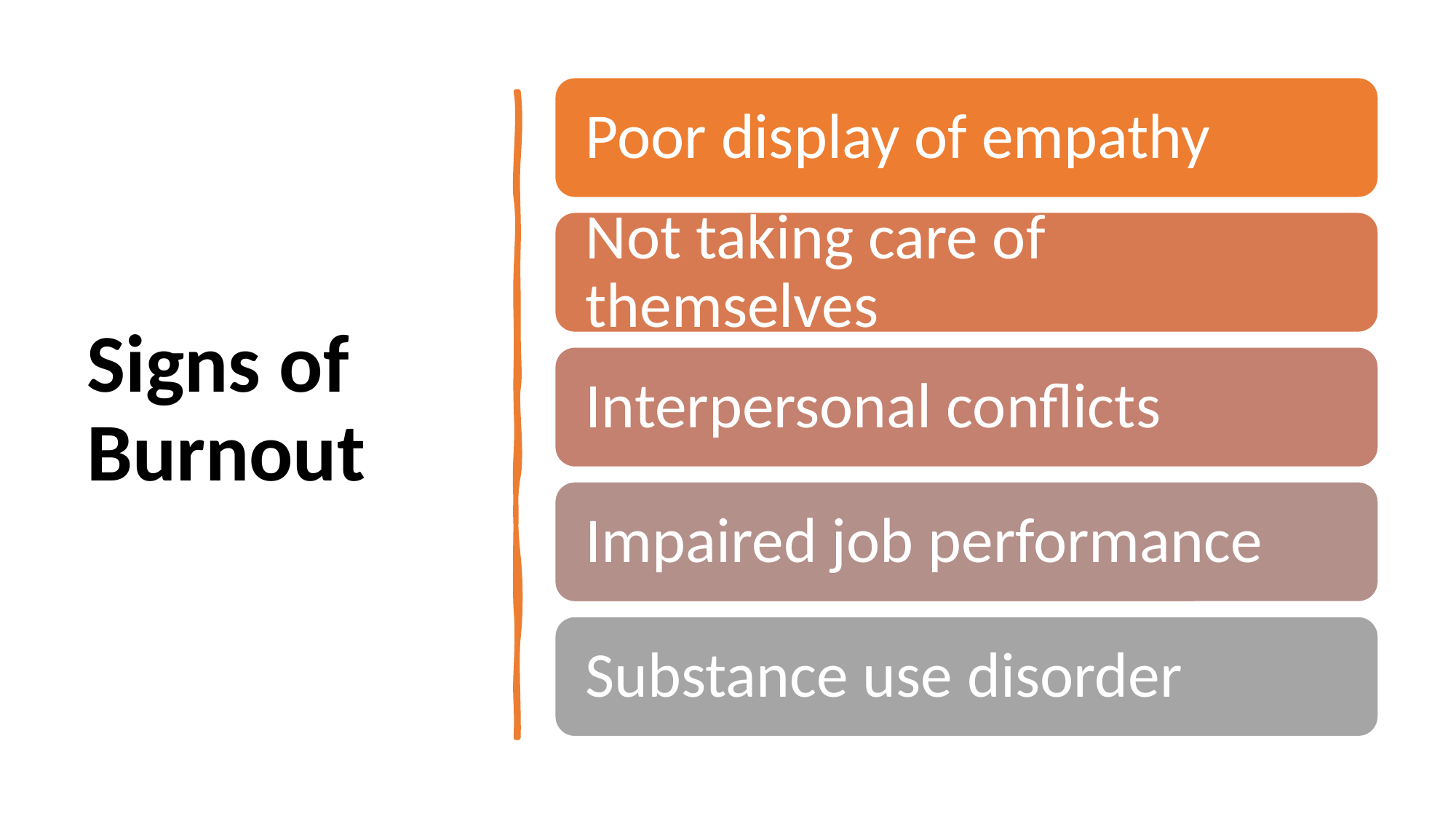

# Signs of Burnout

## Slide 20
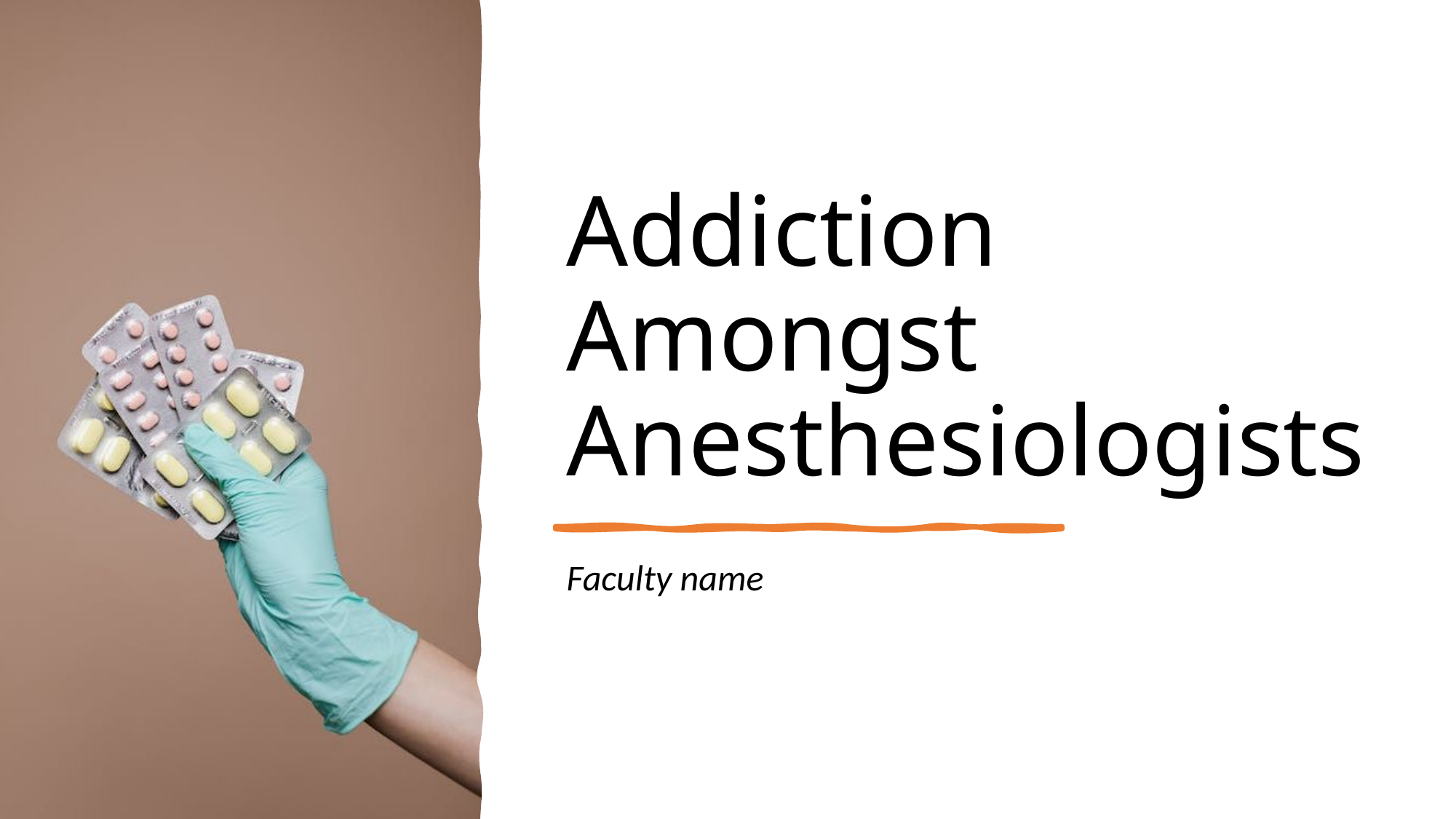

# Addiction Amongst Anesthesiologists
Faculty name

## Slide 21
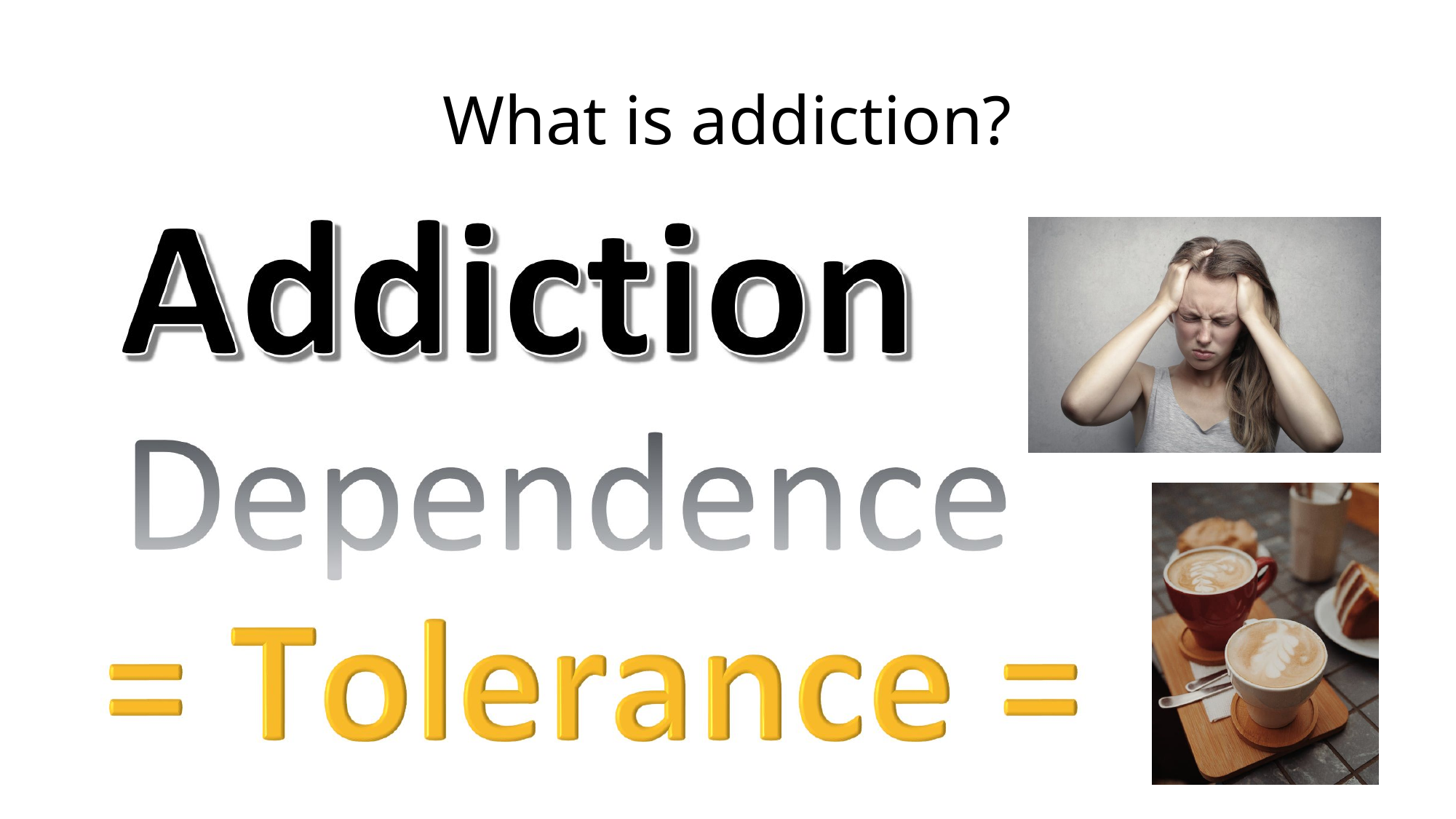

# What is addiction?

## Slide 22
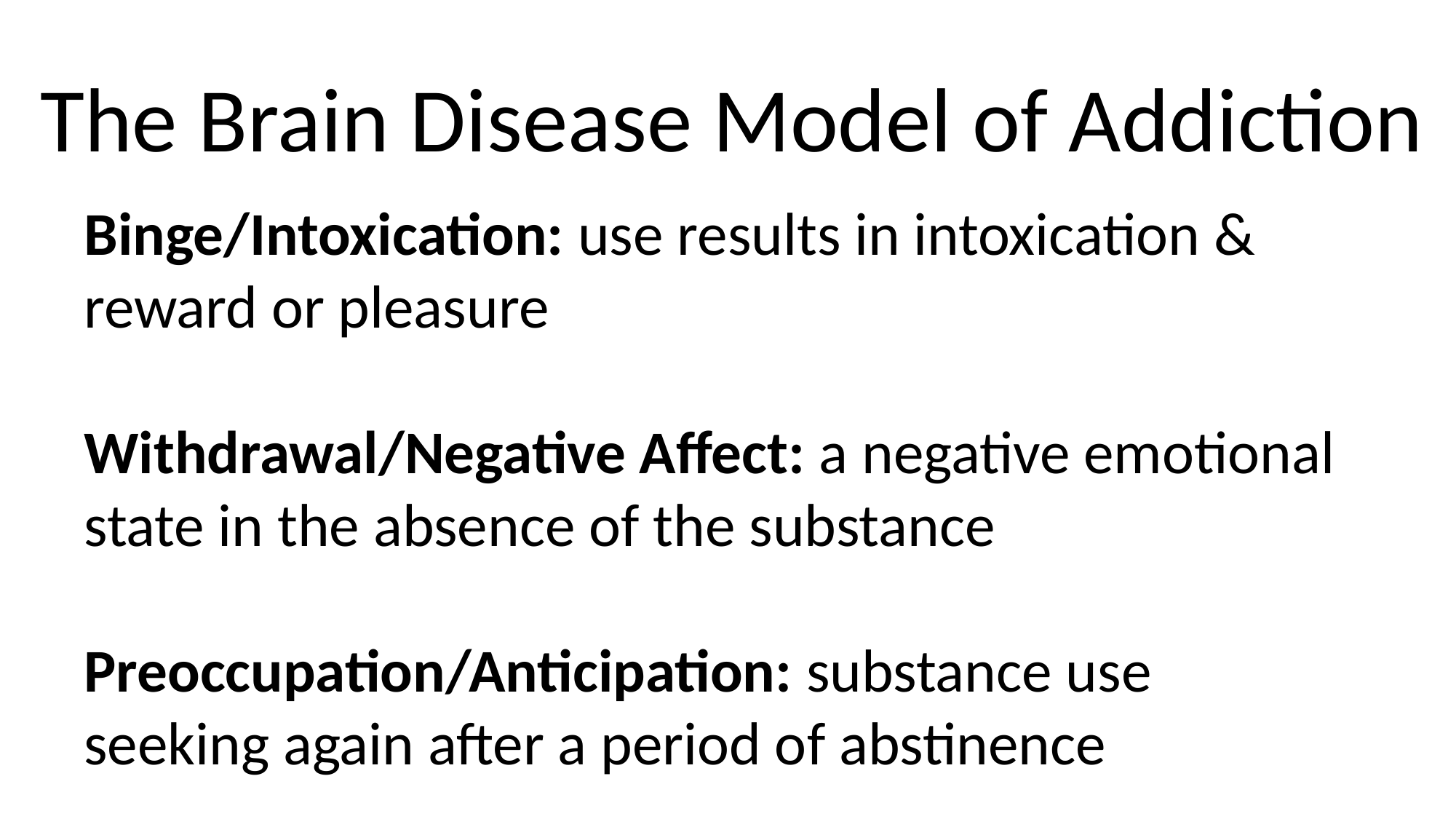

# The Brain Disease Model of Addiction
Binge/Intoxication: use results in intoxication & reward or pleasure
Withdrawal/Negative Affect: a negative emotional state in the absence of the substance
Preoccupation/Anticipation: substance use seeking again after a period of abstinence

## Slide 23
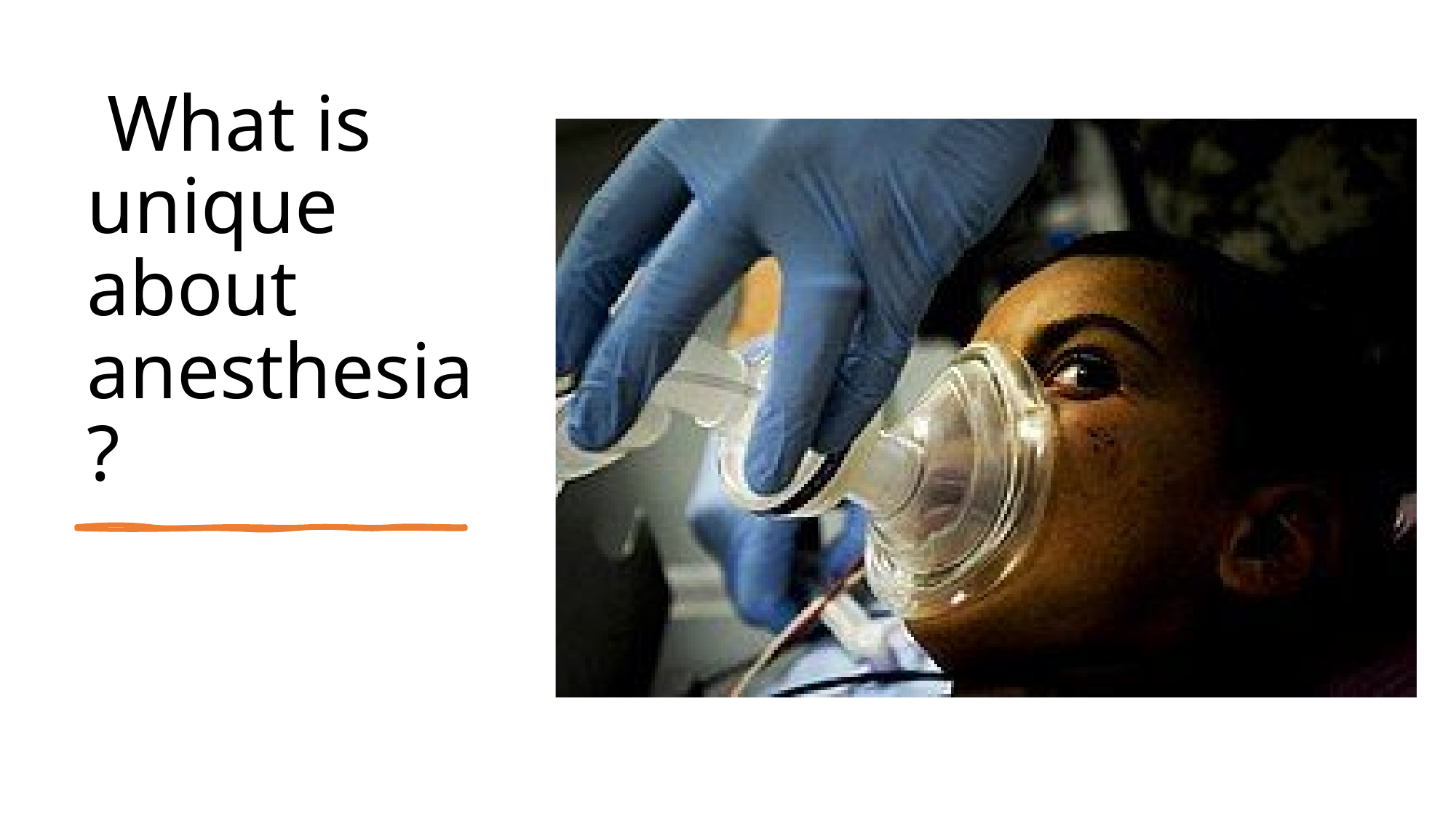

# What is unique about anesthesia?

## Slide 24
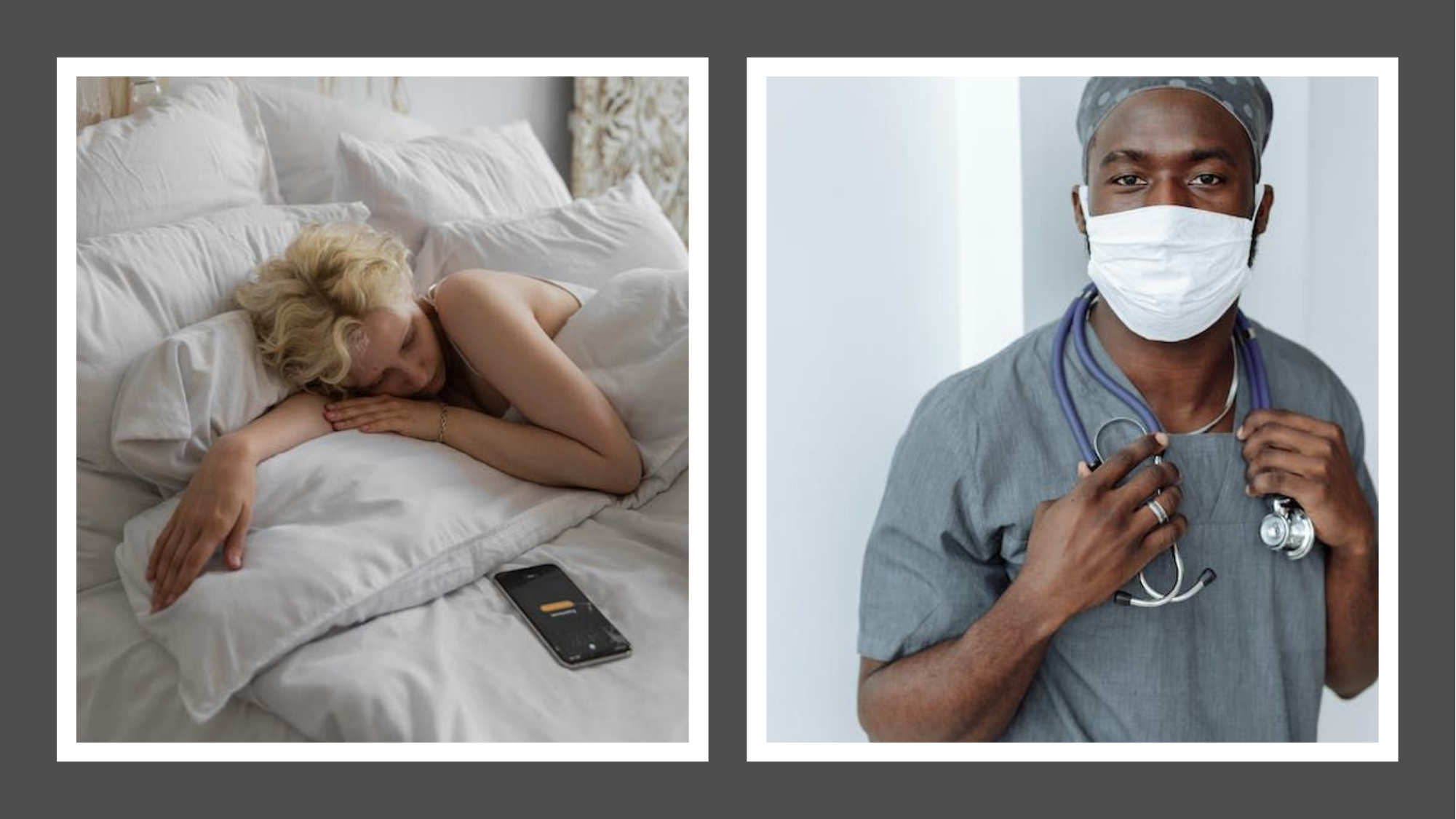

## Slide 25
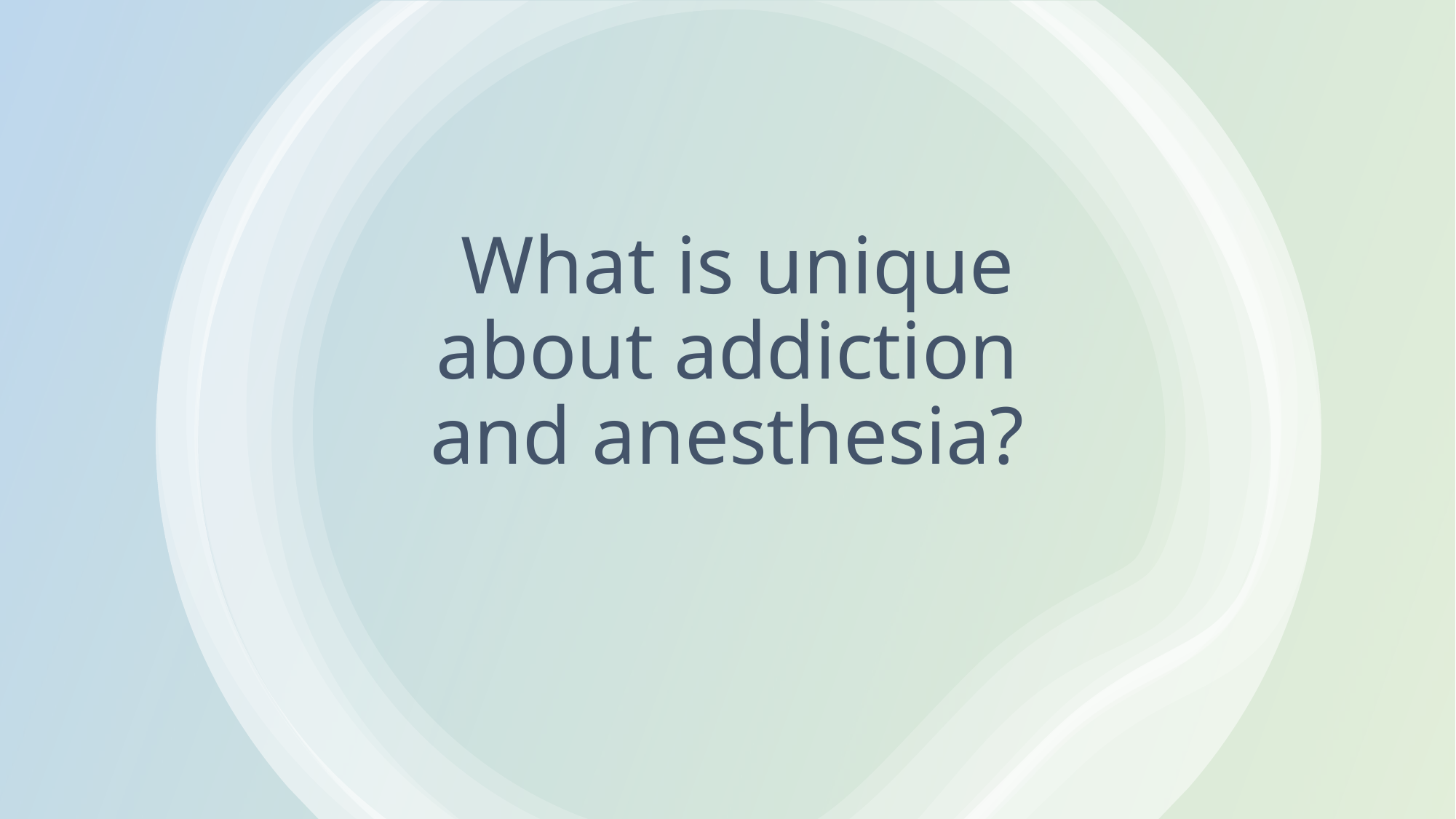

# What is unique about addiction and anesthesia?

## Slide 26
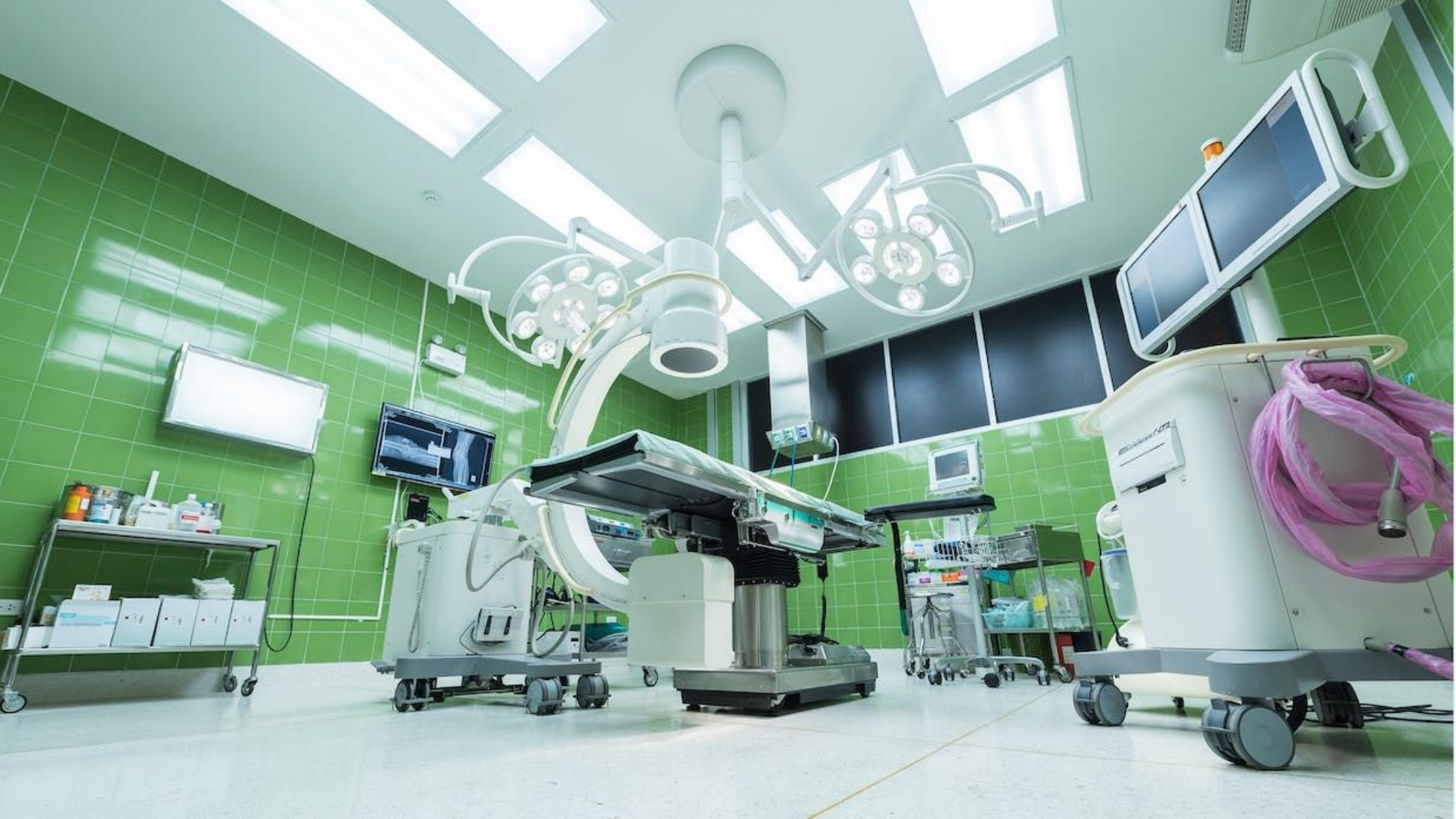

## Slide 27
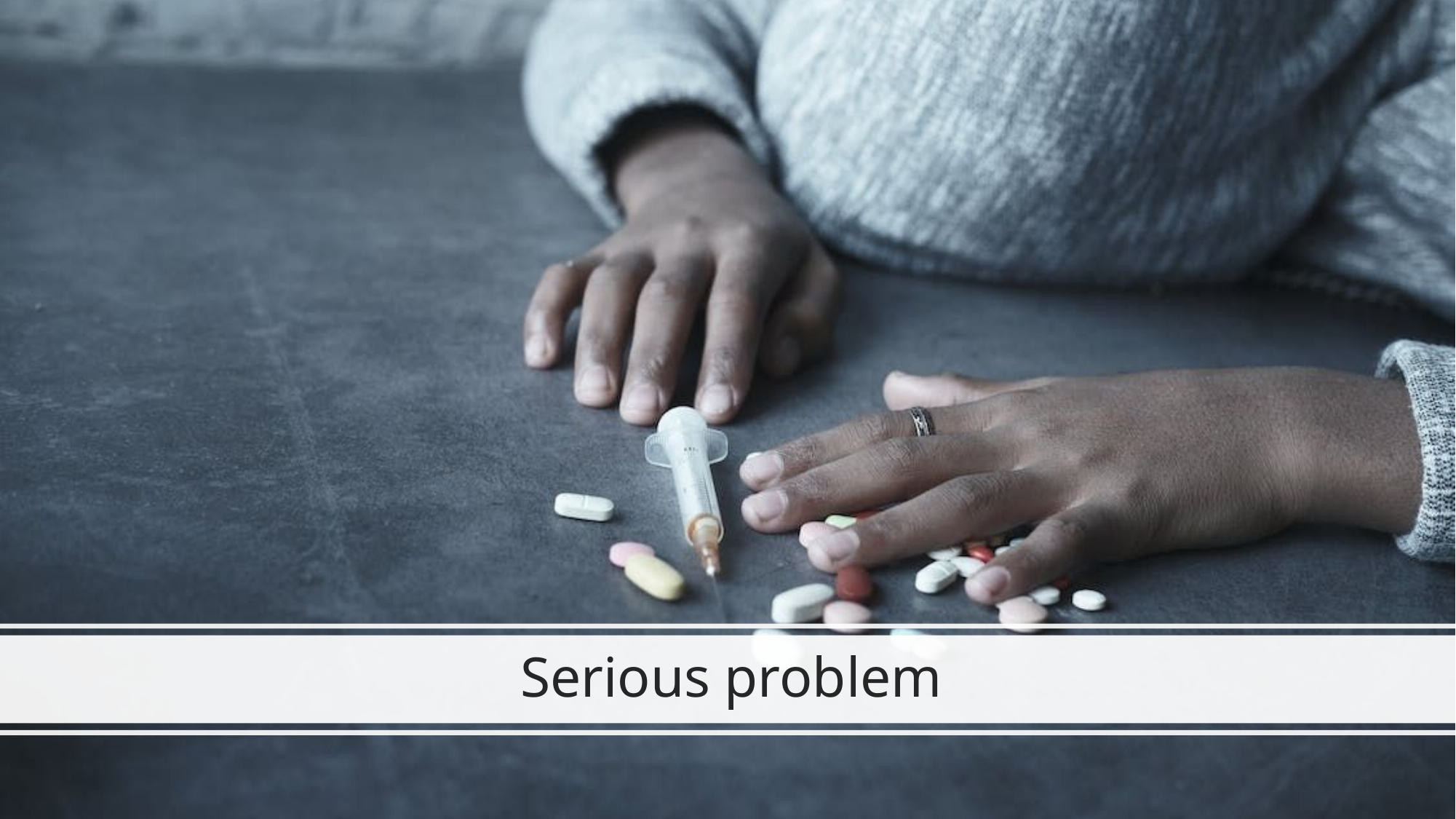

# Serious problem

## Slide 28
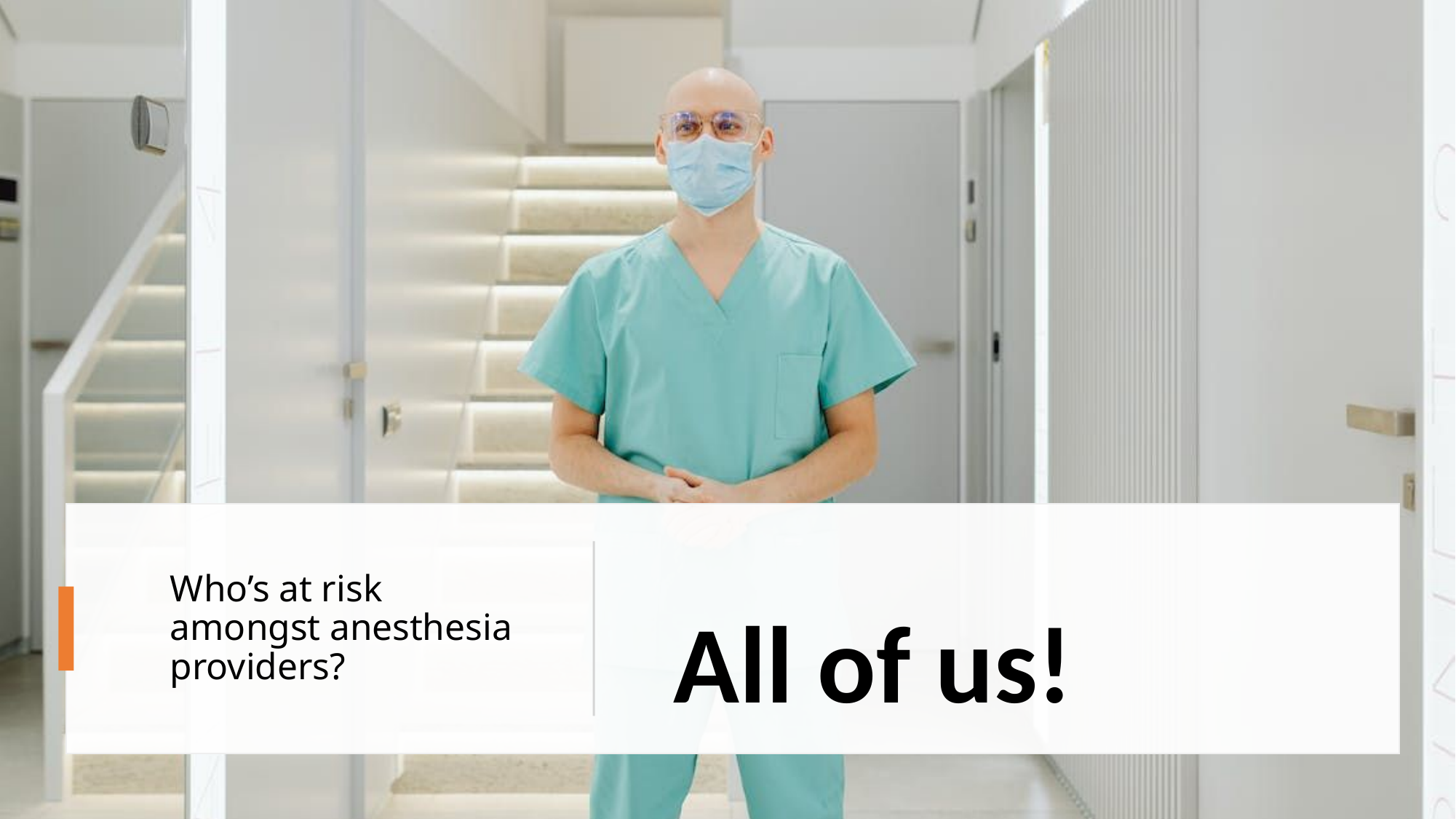

# Who’s at risk amongst anesthesia providers?
All of us!

## Slide 29
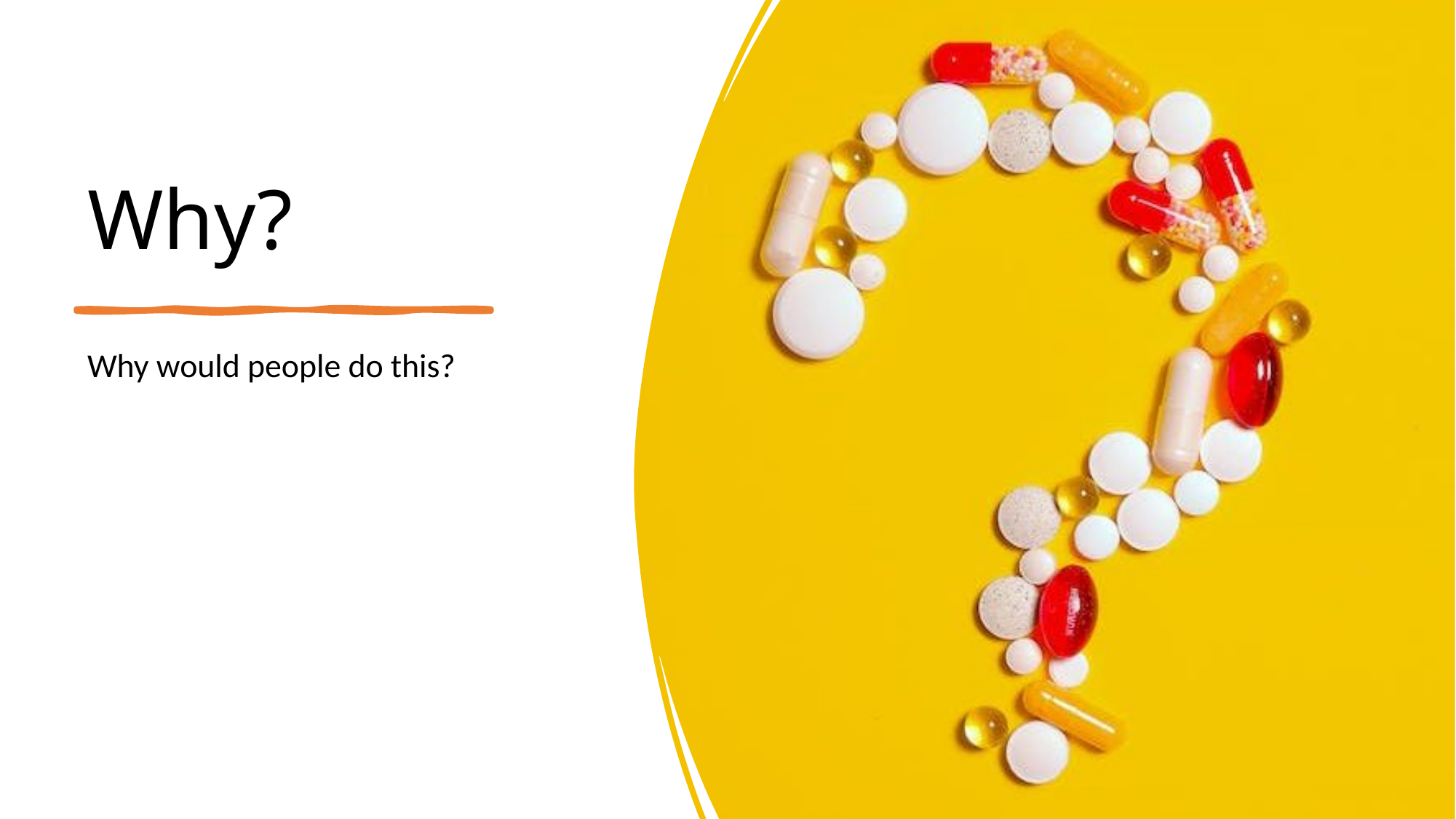

# Why?
Why would people do this?

## Slide 30
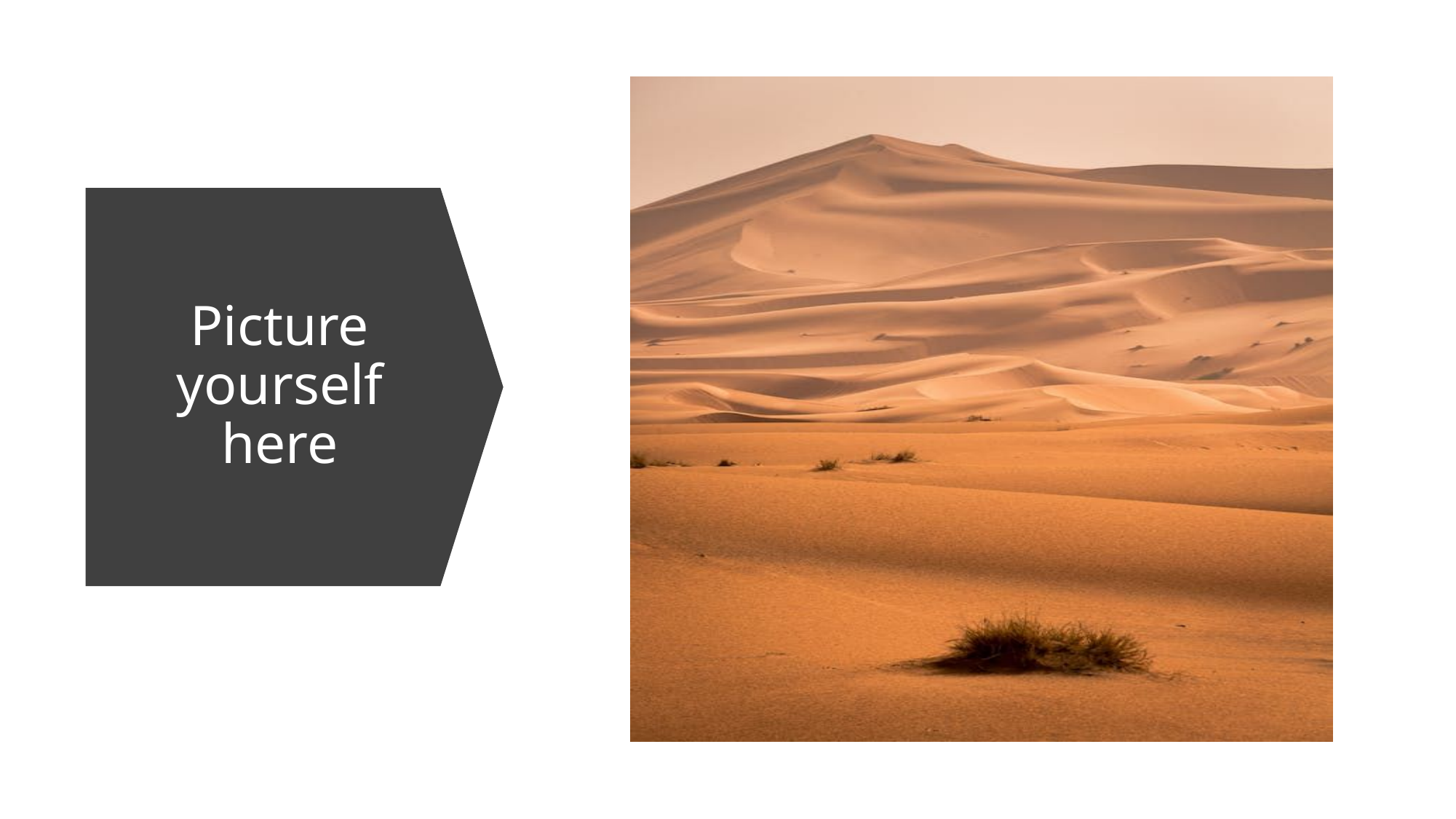

# Picture yourself here

## Slide 31
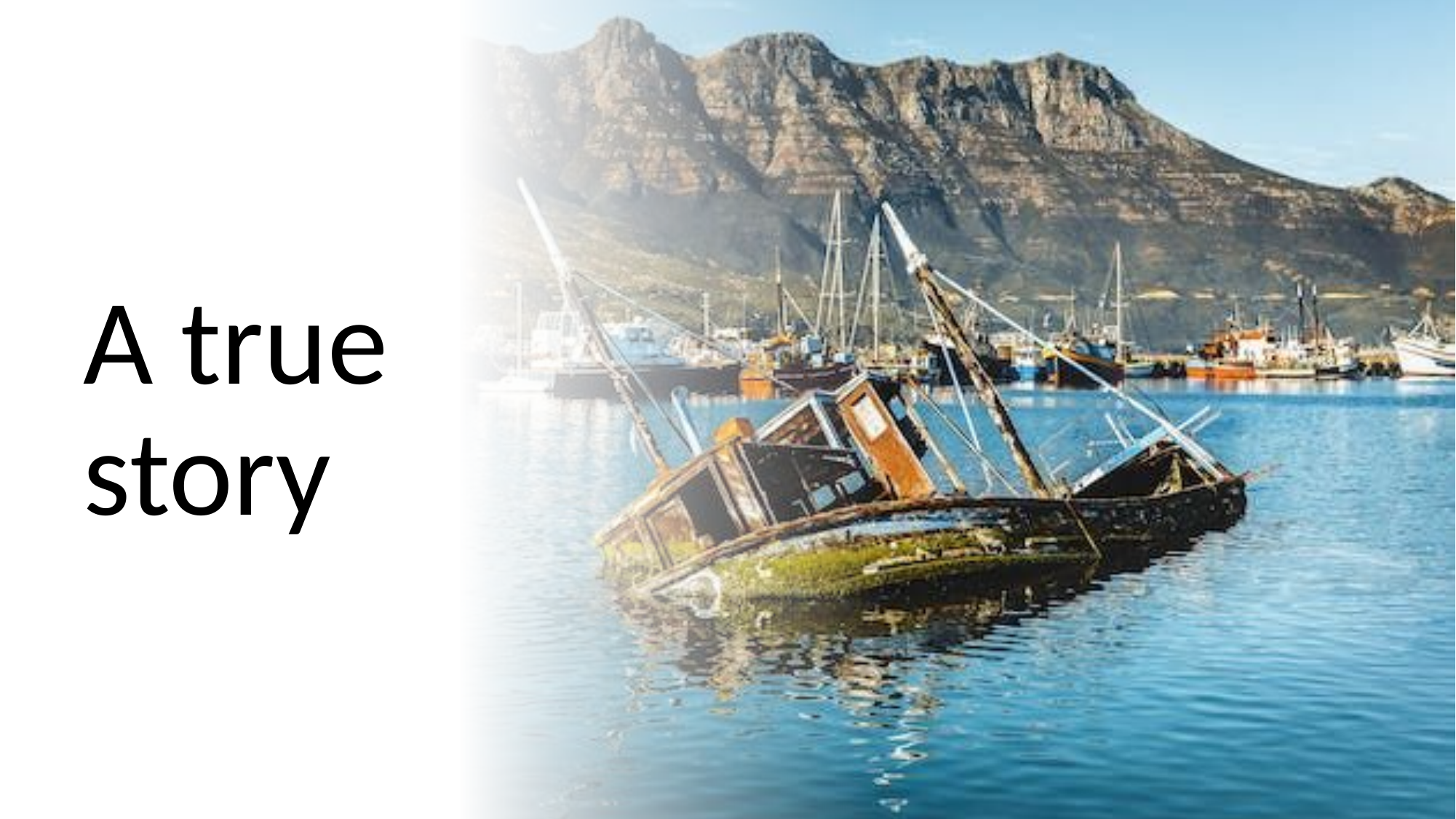

A true story

## Slide 32
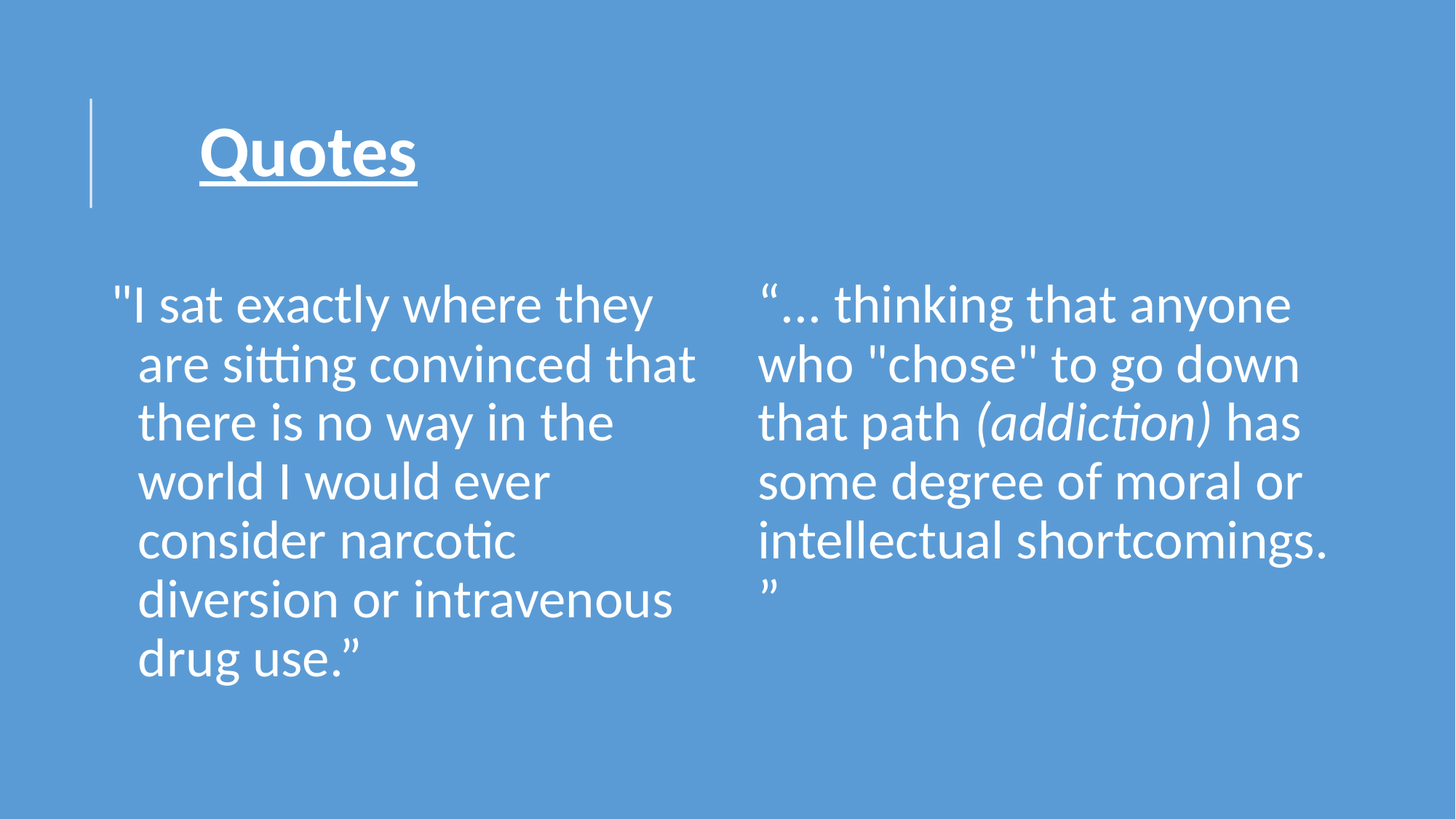

Quotes
"I sat exactly where they are sitting convinced that there is no way in the world I would ever consider narcotic diversion or intravenous drug use.”
“... thinking that anyone who "chose" to go down that path (addiction) has some degree of moral or intellectual shortcomings.”

## Slide 33
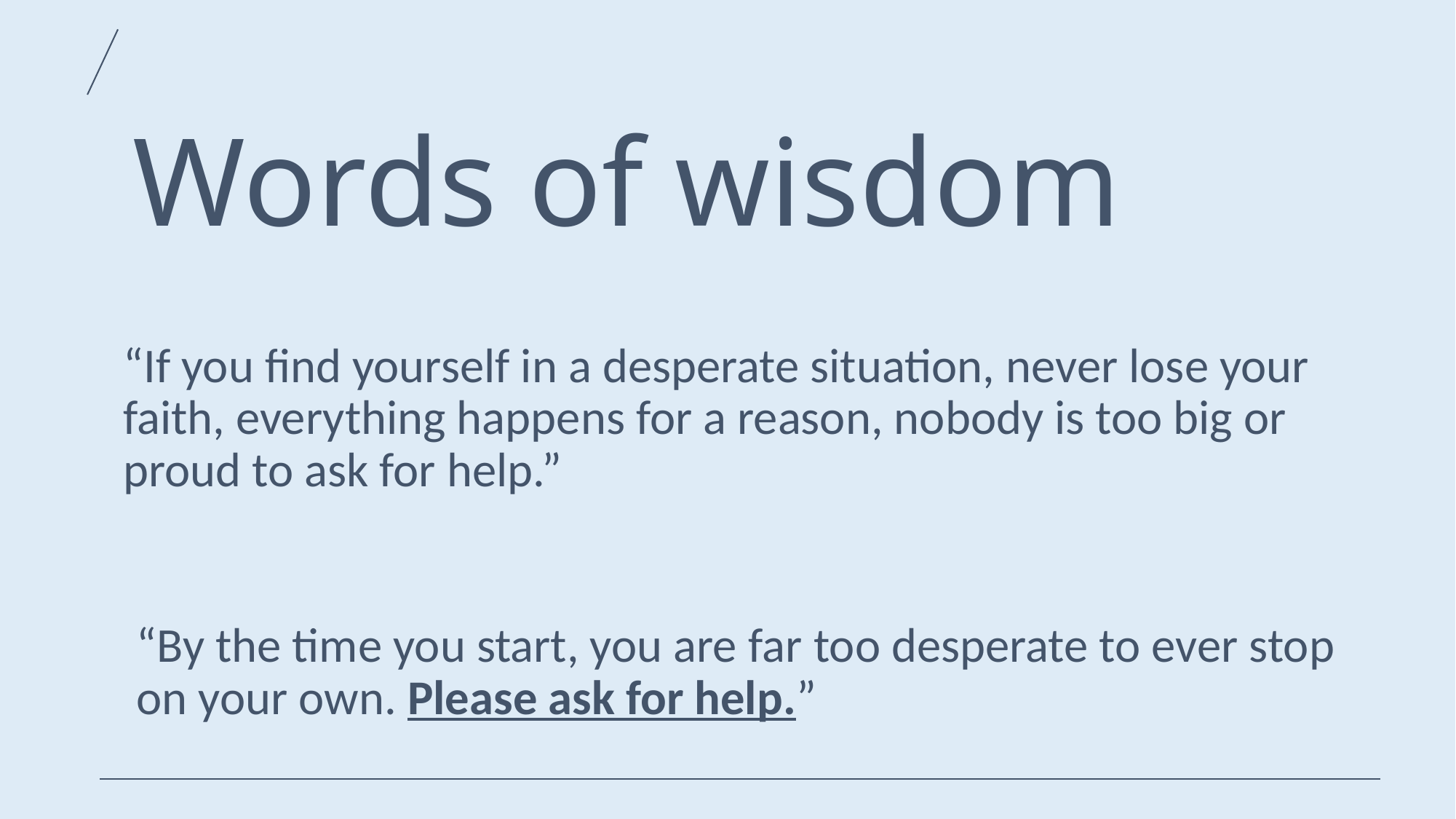

# Words of wisdom
“If you find yourself in a desperate situation, never lose your faith, everything happens for a reason, nobody is too big or proud to ask for help.”
“By the time you start, you are far too desperate to ever stop on your own. Please ask for help.”

## Slide 34
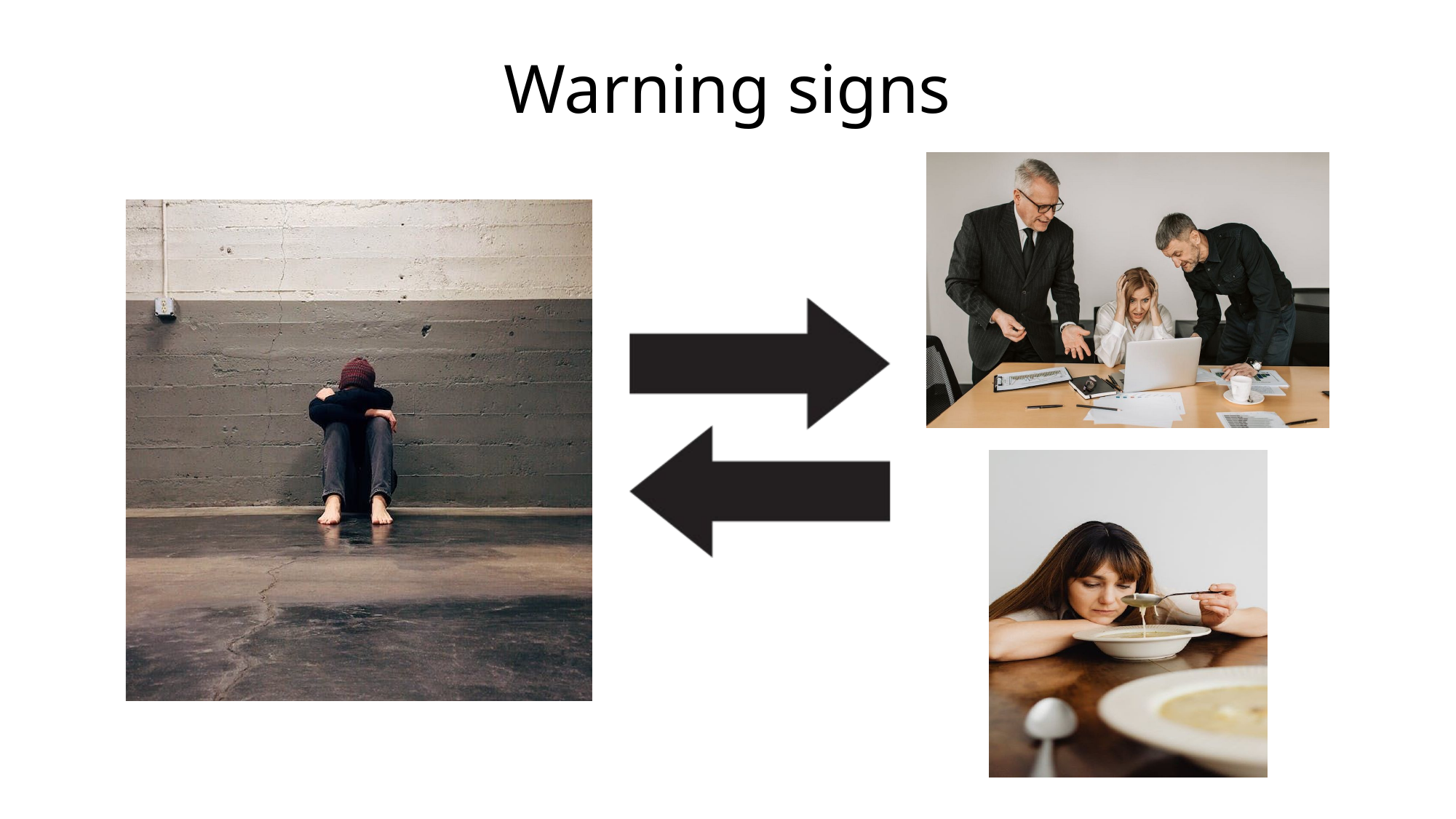

# Warning signs

## Slide 35
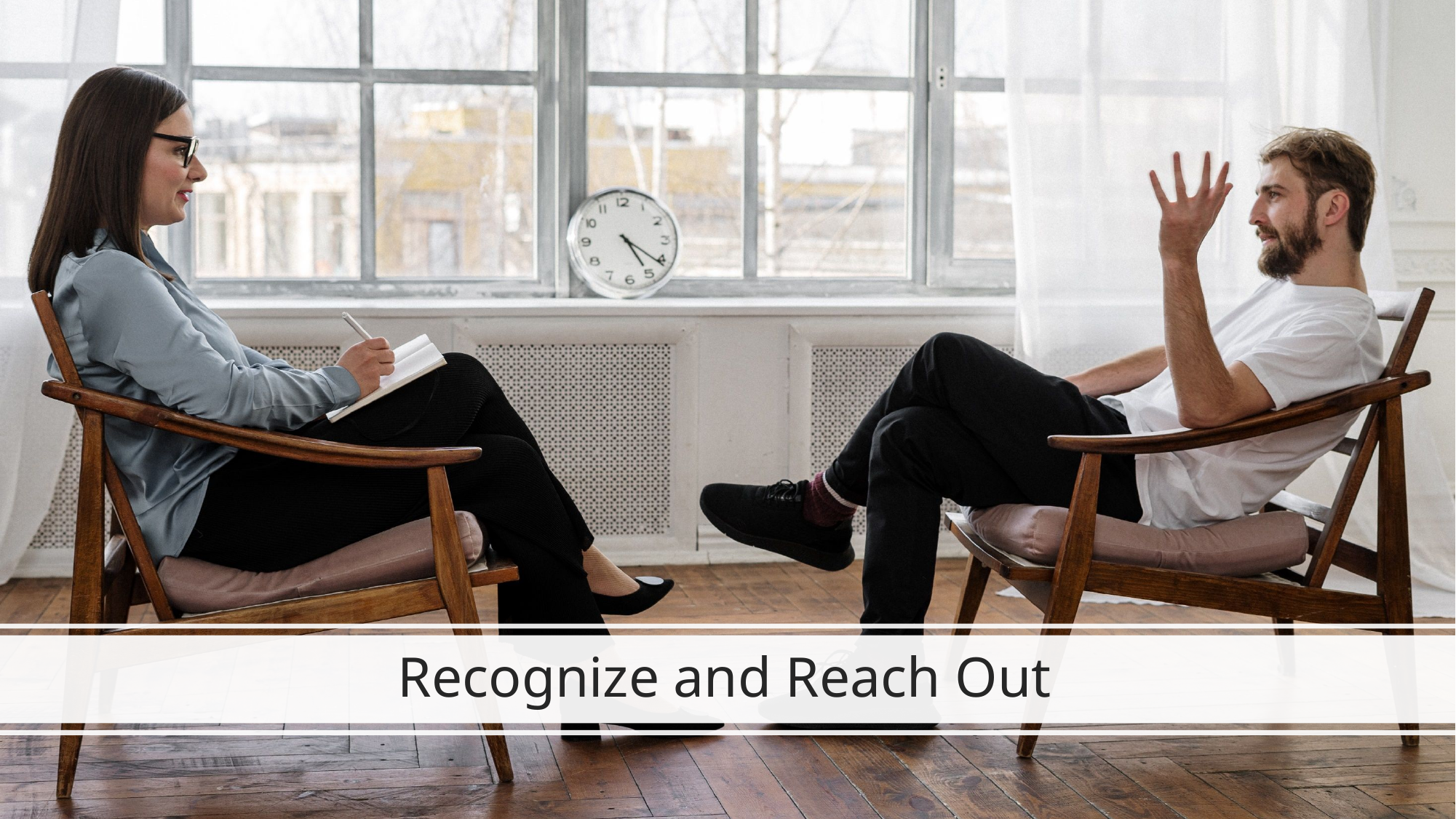

# Recognize and Reach Out

## Slide 36
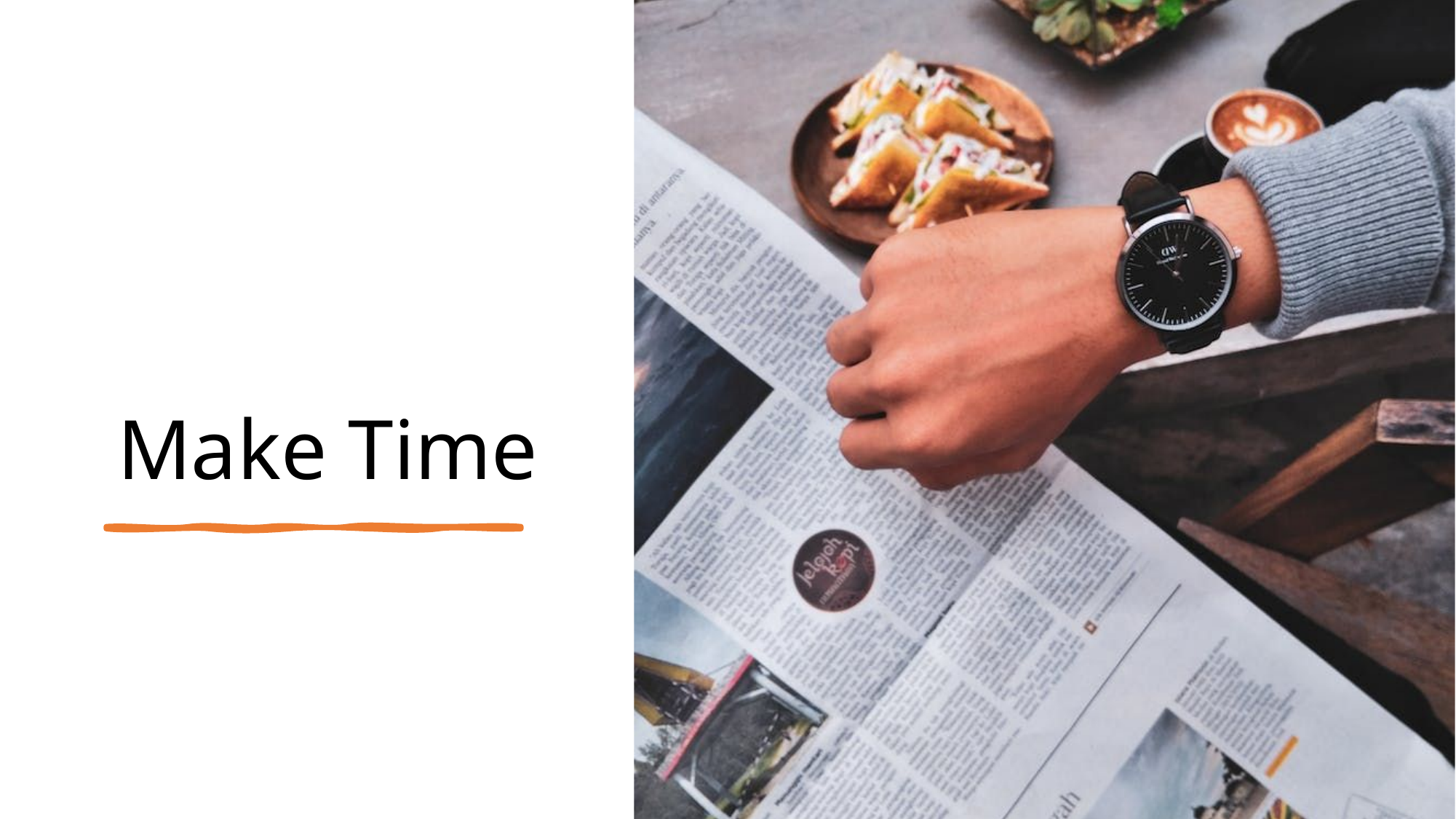

# Make Time

## Slide 37
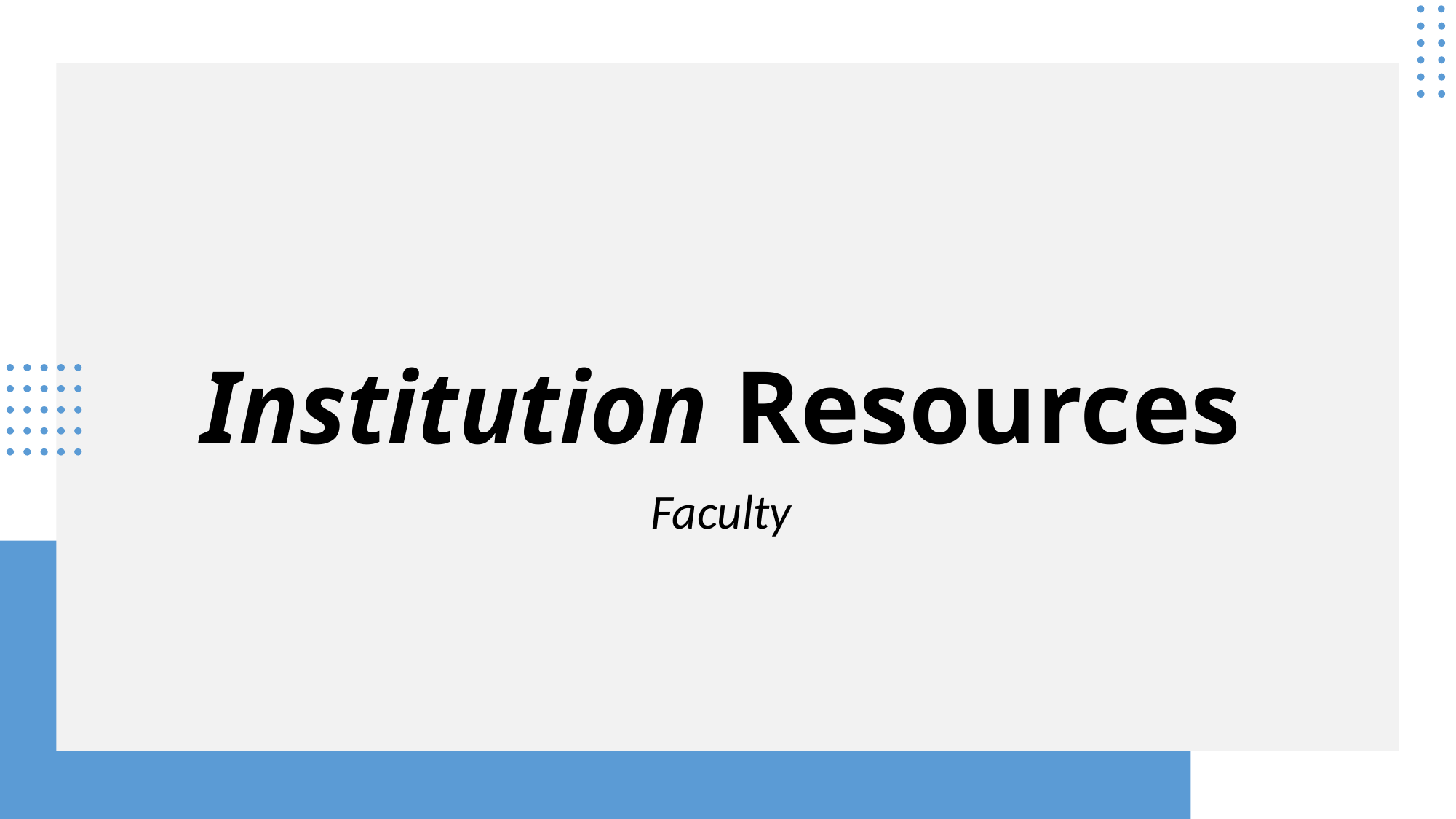

# Institution Resources
Faculty

## Slide 38
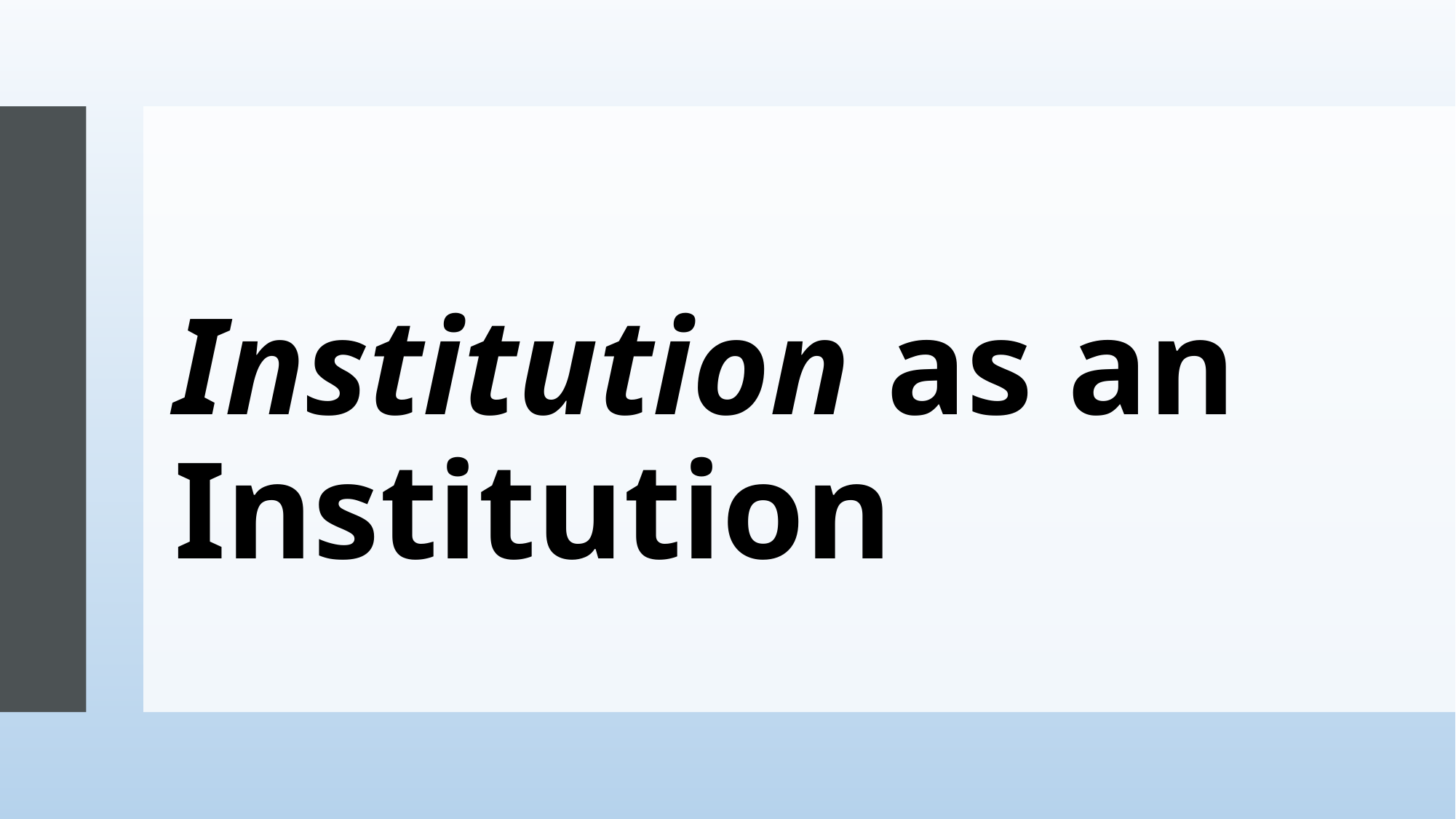

# Institution as an Institution

## Slide 39
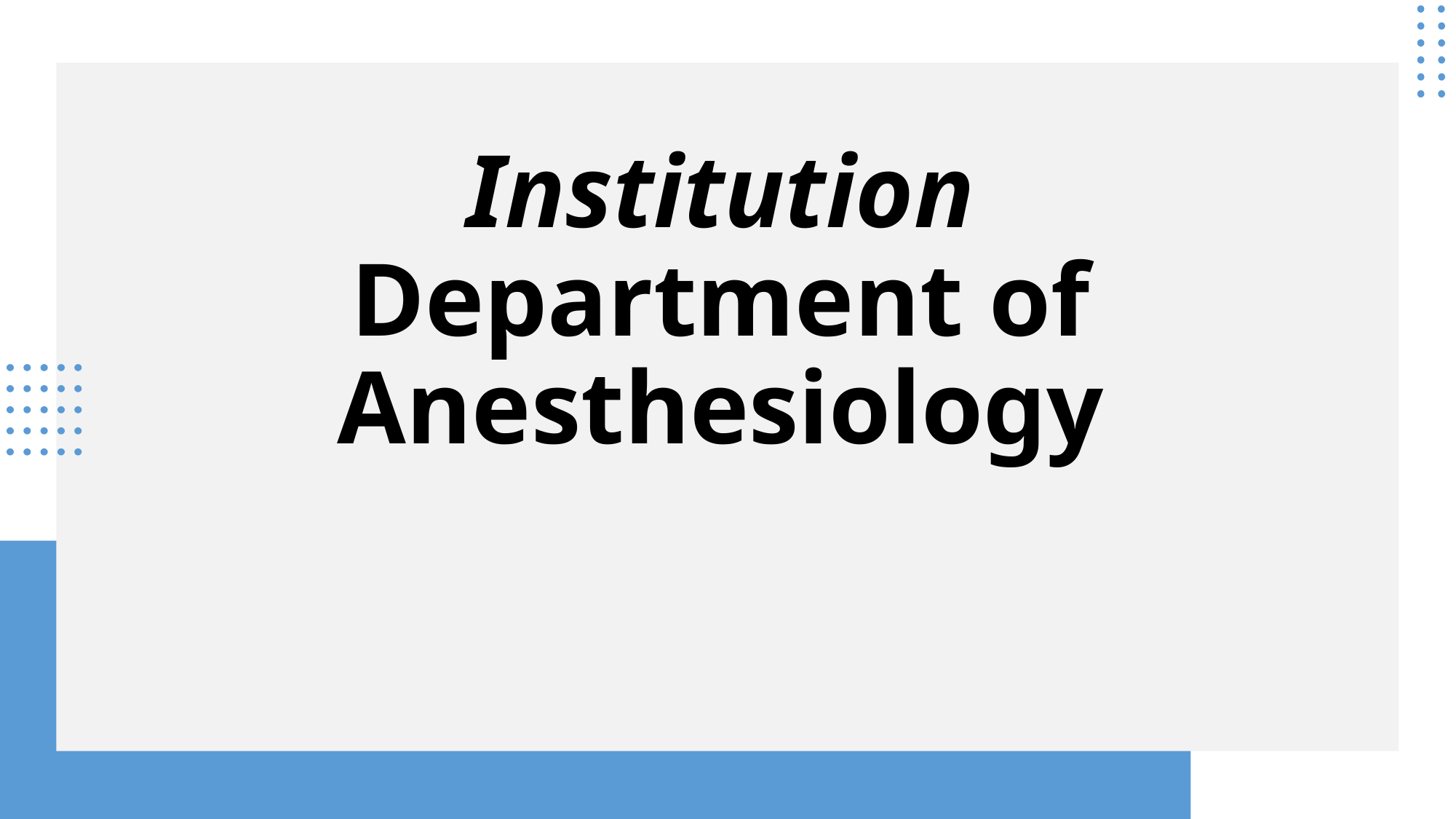

# Institution Department of Anesthesiology

## Slide 40
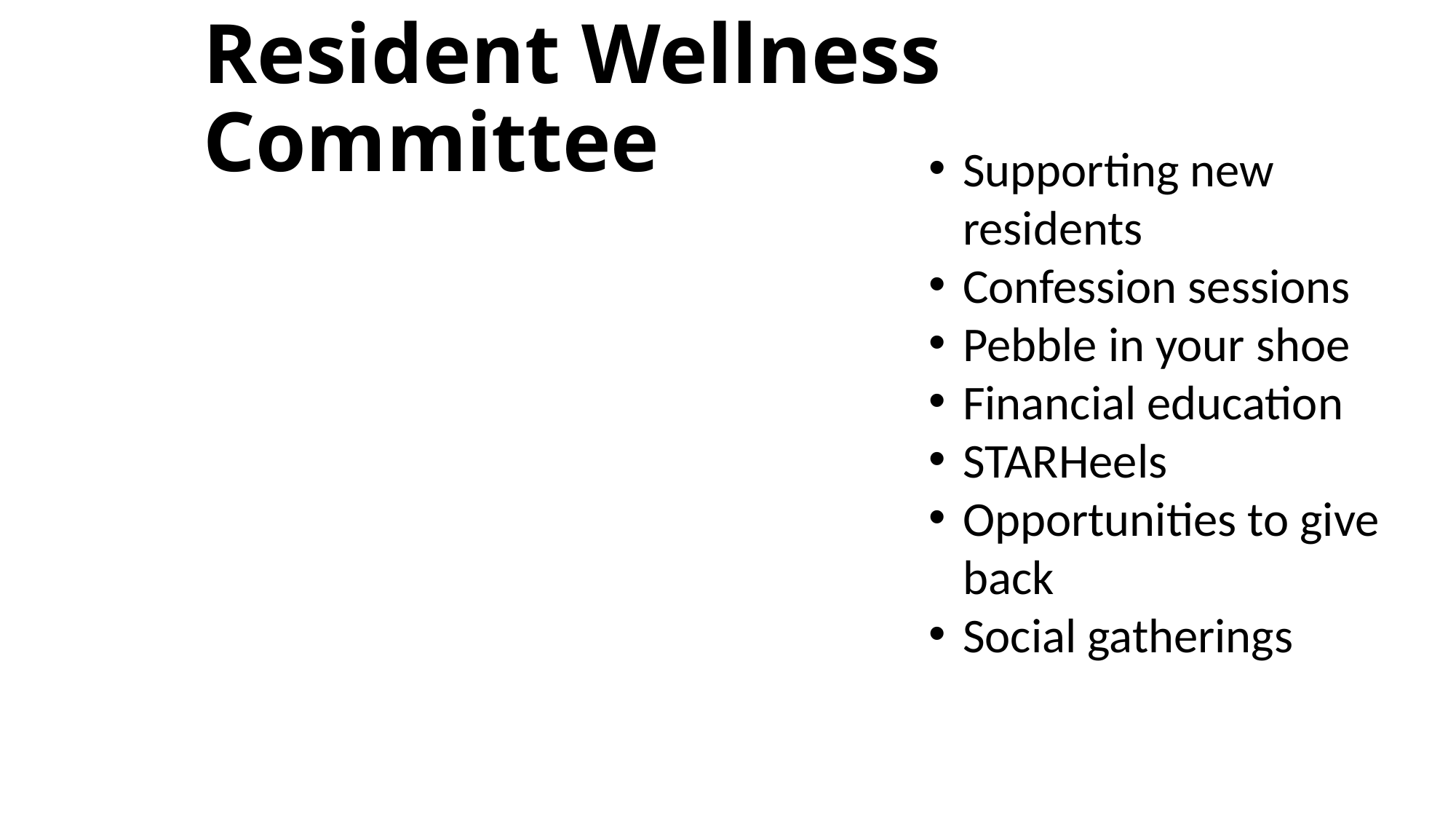

# Resident Wellness Committee
Supporting new residents
Confession sessions
Pebble in your shoe
Financial education
STARHeels
Opportunities to give back
Social gatherings

## Slide 41
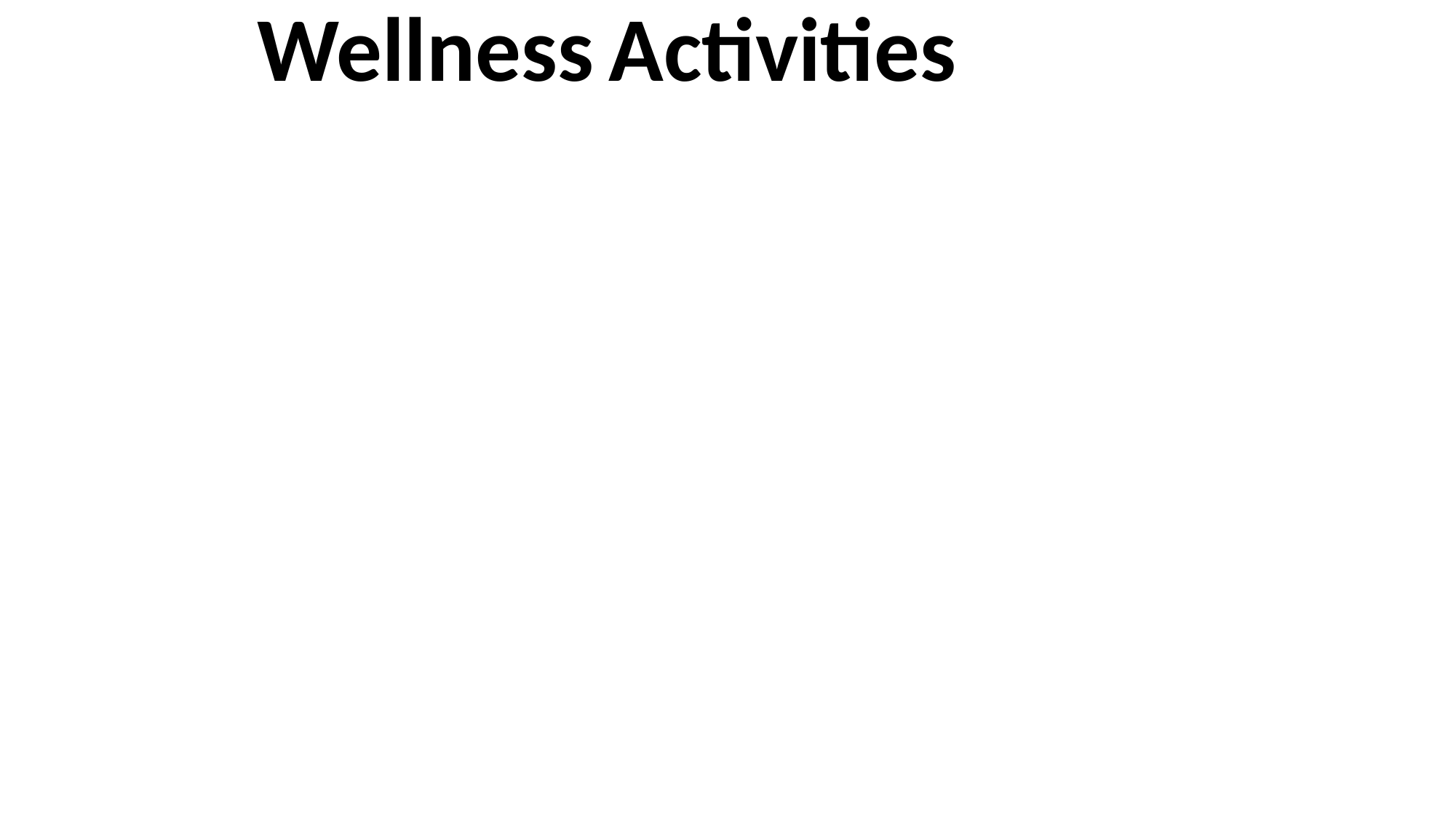

# Wellness Activities

## Slide 42
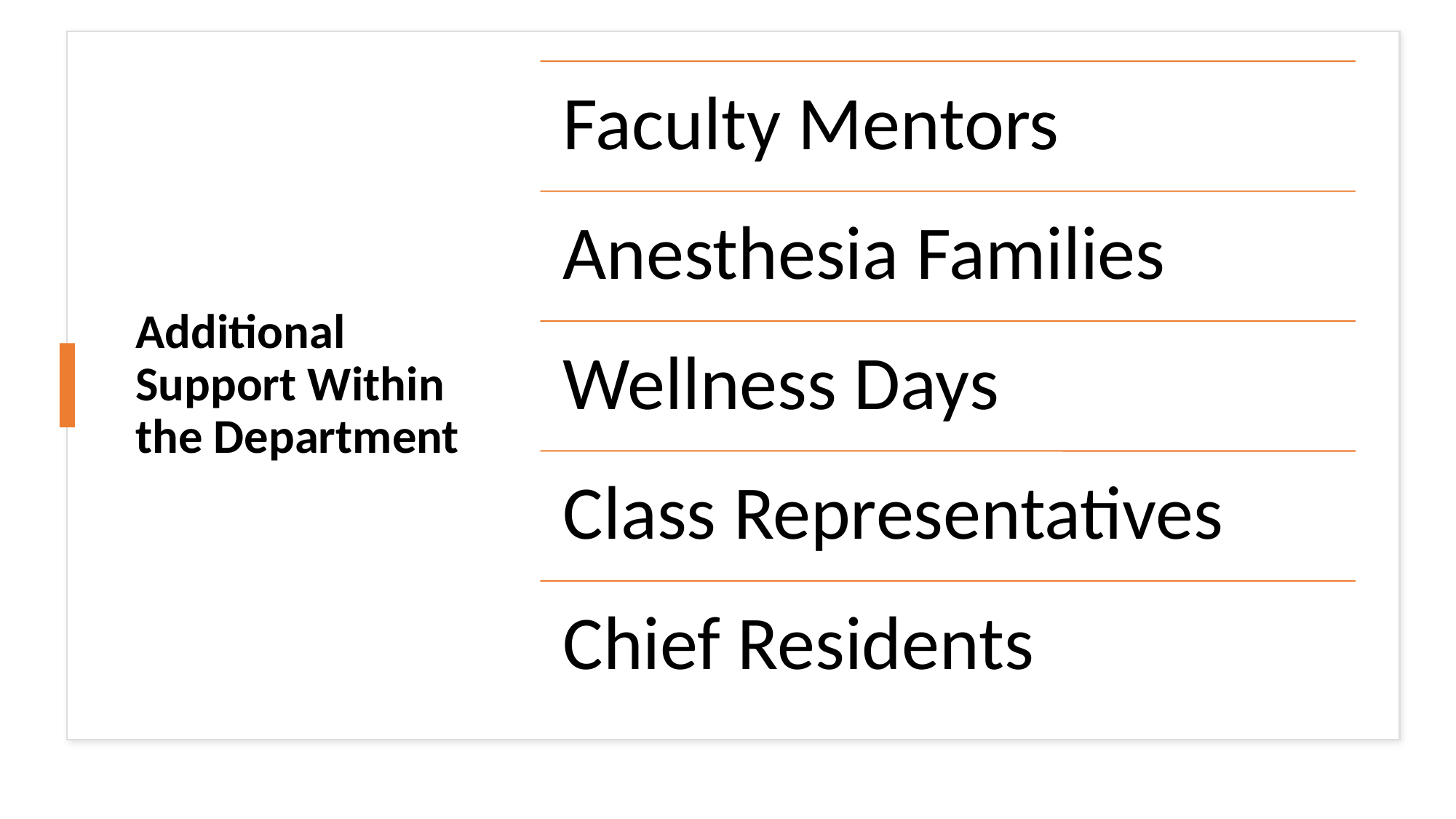

# Additional Support Within the Department

## Slide 43
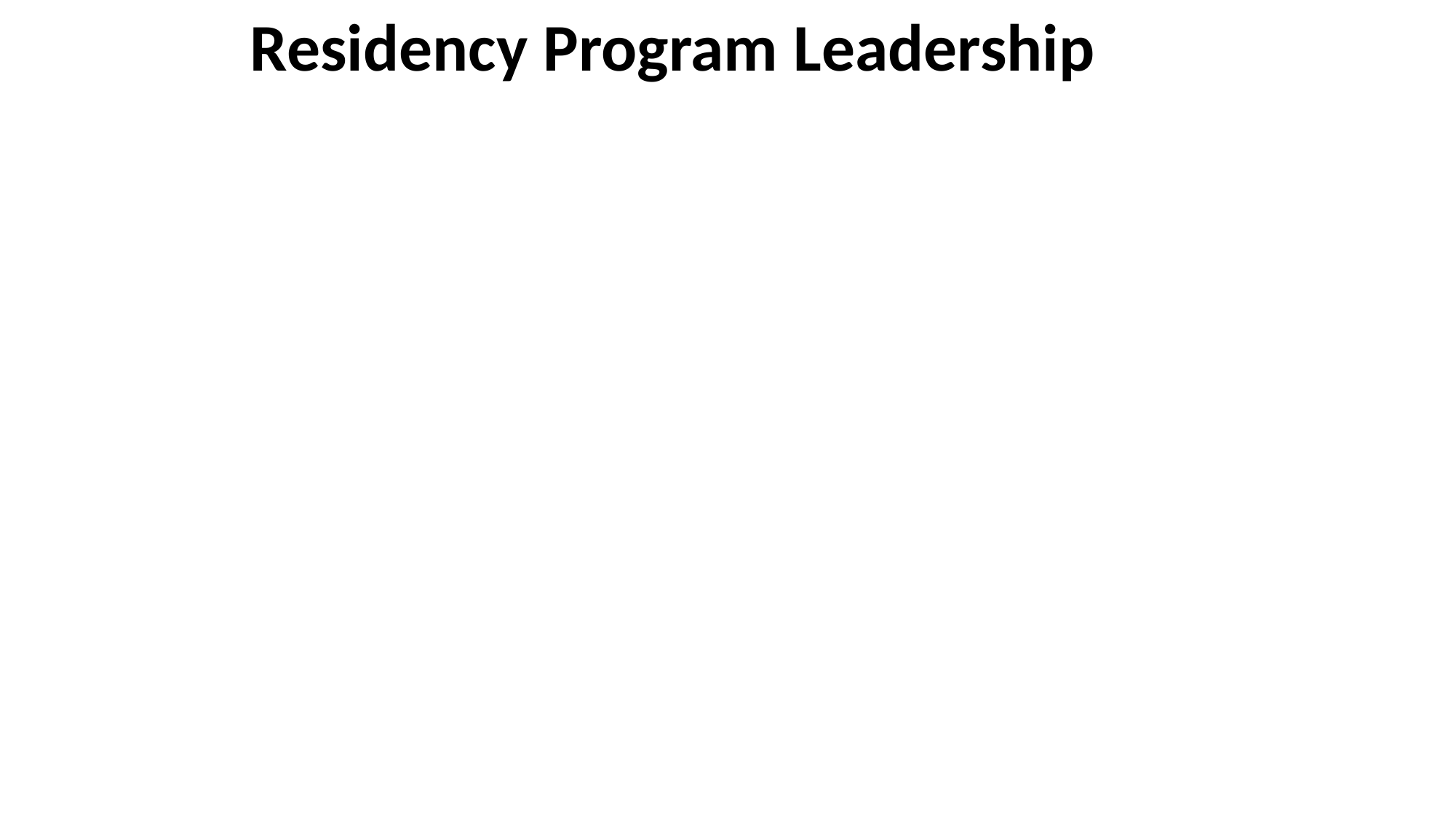

# Residency Program Leadership

## Slide 44
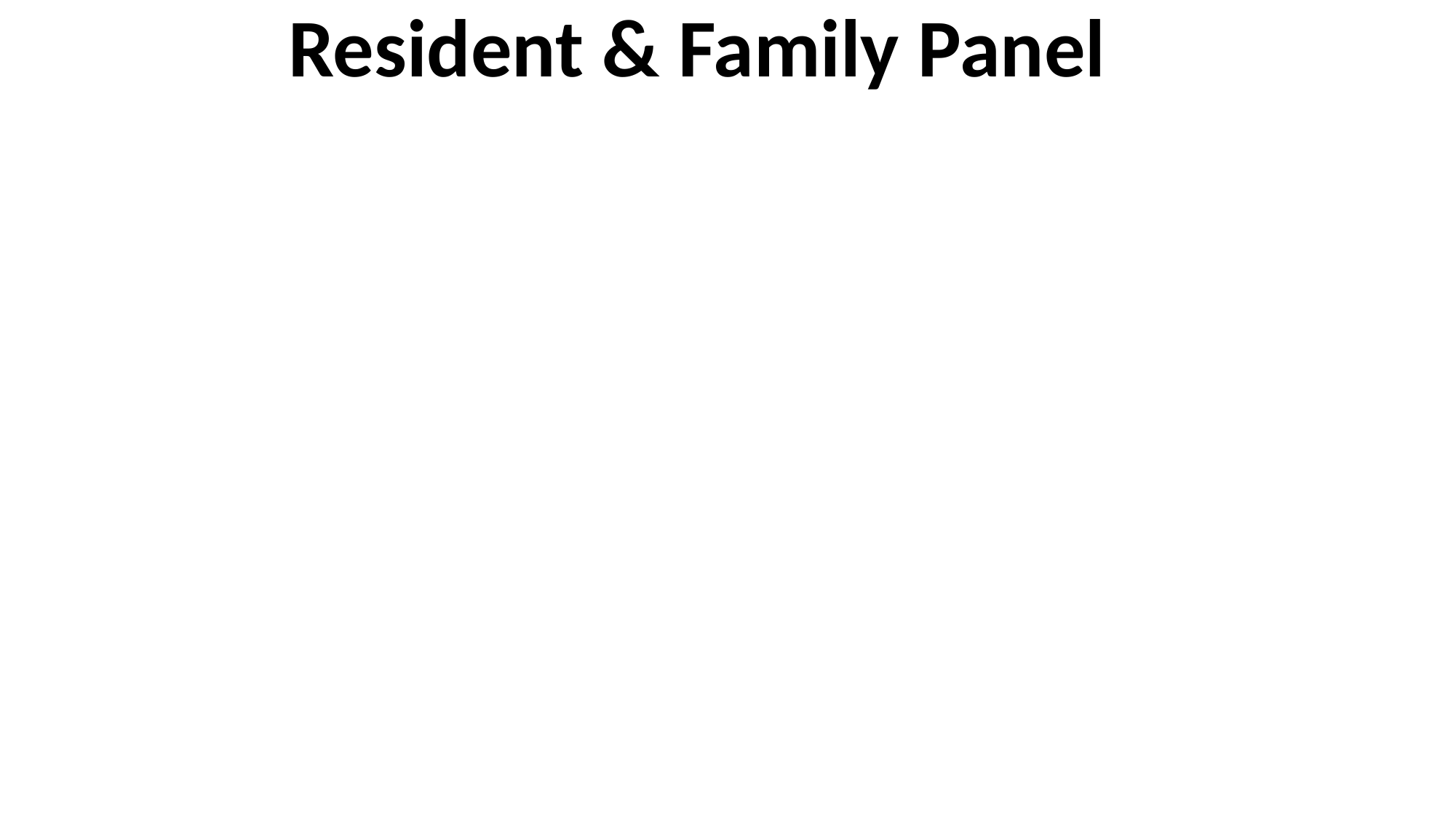

# Resident & Family Panel

## Slide 45
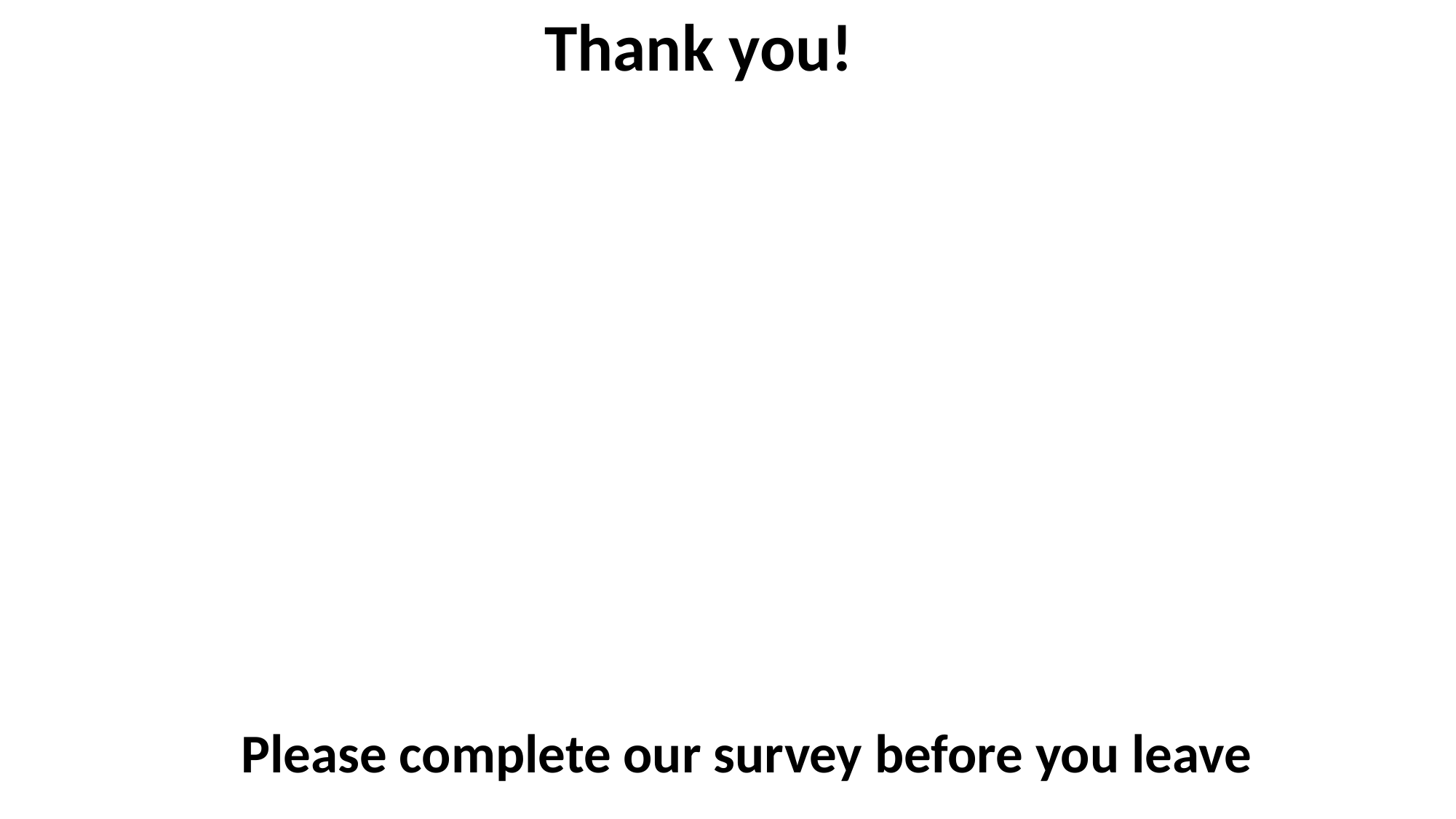

# Thank you!
Please complete our survey before you leave
