# Supplementary material for: Family Anesthesia Experience: Improving Social Support of Residents Through Education of Their Family and Friends
Source: MedEdPORTAL. 2023 Dec 15;19:11370. doi: 10.15766/mep_2374-8265.11370 (PMC10721742; doi:10.15766/mep_2374-8265.11370)
Supplement: Supplementary file 1 — Preevent FAX Checklist.docxSimulation Setup Instructions.docxSchedule of the Day.docxFAX Timeline.docxDay in the Life.mp4Family Day Simulation Scenario.docxHigh-Fidelity Scenario.mp4High-Fidelity Scenario Part 2.mp4Talking Points for Simulation.docxDidactics.pptxPanel Questions and Logistics.docxPostevent Survey.docx [file mep_2374-8265.11370-s001.zip › K. Panel Questions and Logistics.docx]

Resident Support Person Panel

This document provides a description of the resident/support person panel and questions that can be asked to the panelists.

The panel should consist of 3 senior residents and their support persons. Ideally, the residents will be a diverse group (gender, marital status, parental status, race/ethnicity, etc.), and the support persons will represent a diverse group (parents, spouse, children, etc.). A faculty member will moderate the panel.

The photos of people on the panel can be displayed on a TV for the CA-1 residents and their support persons to see. A faculty member will introduce the purpose of the panel and the panelists. This should take about 5 minutes.

1. Resident 1: Describe vacation allotment, how and when it is selected, flexibility allowed, etc.
2. Resident 1: Describe weekend work requirements.
3. Resident 2: Describe the standardized tests (boards and ITE) and the requirements for board certification
4. Resident 2: Describe work requirements outside of clinical duties (i.e. projects, studying, etc.)
5. Resident 3: Why do you have to call your attending every night—it’s so inconvenient!
6. Resident 3: Describe duty hour rules and ABA rules on number of days off allowed during clinical anesthesia
7. All Participants: What is the hardest part of residency for the resident? For the family?
8. All residents: How do you stay “balanced” during residency?
9. All Support Persons: What advice do you have for friends and family of residents?

Take time to incorporate questions from the audience.
