# Supplementary material for: Family Anesthesia Experience: Improving Social Support of Residents Through Education of Their Family and Friends
Source: MedEdPORTAL. 2023 Dec 15;19:11370. doi: 10.15766/mep_2374-8265.11370 (PMC10721742; doi:10.15766/mep_2374-8265.11370)
Supplement: Supplementary file 1 — Preevent FAX Checklist.docxSimulation Setup Instructions.docxSchedule of the Day.docxFAX Timeline.docxDay in the Life.mp4Family Day Simulation Scenario.docxHigh-Fidelity Scenario.mp4High-Fidelity Scenario Part 2.mp4Talking Points for Simulation.docxDidactics.pptxPanel Questions and Logistics.docxPostevent Survey.docx [file mep_2374-8265.11370-s001.zip › L. Postevent Survey.docx]

INSTRUCTIONS

This document provides a post Family Anesthesia Experience survey that can be distributed to CA-1 residents and their support persons.

*In order to optimize response rate, please consider administering this survey at the conclusion of the Family Anesthesia Experience event via paper and pencil format. You could also upload the questions to a preferred survey platform and administer the survey using QR codes (and personal phones) or tablets instead.*

*If you plan to use this information for research, check with your institutional IRB about their requirements. If you do not plan to use this information for research, please remove that statement from the opening directions of the survey.*

*Note: The authors adapted this survey from the survey used in Martinelli SM, Isaak RS, Chidgey BA, et al. Family Comes First: A Pilot Study of the Incorporation of Social Support Into Resident Well-being. J Educ Perioper Med. 2020;22(4):E652.*

**UNC Family Anesthesia Experience (FAX) 2022 Post Experience Survey**

We want to thank you for attending the Family Anesthesia Experience Day and hope you enjoyed it. Please complete this short survey to help us improve the program. All survey responses are anonymous and will be used for program improvement and research. We appreciate your participation!

1. I am a…

[] resident [] resident’s partner [] resident’s parent [] resident’s friend [] other________

1. Which gender do you identify with?

[] female [] male [] transgender woman [] transgender man [] non-binary/ third gender [] prefer to not say

[] prefer to self-describe __________________________________

1. Which race do you identify with?

[] Non-Hispanic White

[] Non-Hispanic Black

[] Hispanic

[] Asian

[] Native Hawaiian or Other Pacific Islander

[] American Indian or Alaskan Native

[] prefer not to say

[] prefer to self-describe ___________________________________________________

1. How old are you? ________
2. Please rate how strongly you agree or disagree with the following statements about the event.

I enjoyed participating in this event.

[] strongly agree [] agree [] disagree [] strongly disagree

I would recommend this event to other support persons of anesthesia residents.

[] strongly agree [] agree [] disagree [] strongly disagree

I believe that this event will improve communication and support between the resident and their support persons.

[] strongly agree [] agree [] disagree [] strongly disagree

1. How much did you learn from this event?

[] I did not learn anything [] a small amount [] a moderate amount [] a large amount

1. How far away does the resident live from the support person(s) who attended this event? Select all that apply.

[] we live together

[] we live within a 30-minute drive from each other

[] we live between a 30-minute to 2-hour drive from each other

[] we live between a 2 to 8-hour drive from each other

[] I live in the US, but we live beyond an 8-hour drive from each other

[] I live abroad

1. What did you like most about this event? Check all that apply.

[] day in the Life video

[] demonstration of airway management

[] demonstration of procedures (e.g., central line, neuraxial, and peripheral nerve blocks)

[] demonstration of intraoperative experience

[] meal/networking

[] lecture on wellness and burnout

[] lecture on substance use disorder

[] lecture on local wellness resources

[] resident and support person panel

[] being provided with contact information for the program leadership

[] Other_________________________________________________________________

1. What is the most helpful piece of information you gained from this event?
2. What can be improved (e.g., flow of the event, topics discussed, etc.)?
3. If you have other wellness promoting event/program ideas that would be beneficial to residents and their support persons, please list them below.
4. If you have any other comments about the program, please add them here.
