# Supplementary material for: Family Anesthesia Experience: Improving Social Support of Residents Through Education of Their Family and Friends
Source: MedEdPORTAL. 2023 Dec 15;19:11370. doi: 10.15766/mep_2374-8265.11370 (PMC10721742; doi:10.15766/mep_2374-8265.11370)
Supplement: Supplementary file 1 — Preevent FAX Checklist.docxSimulation Setup Instructions.docxSchedule of the Day.docxFAX Timeline.docxDay in the Life.mp4Family Day Simulation Scenario.docxHigh-Fidelity Scenario.mp4High-Fidelity Scenario Part 2.mp4Talking Points for Simulation.docxDidactics.pptxPanel Questions and Logistics.docxPostevent Survey.docx [file mep_2374-8265.11370-s001.zip › C. Schedule of the Day.docx]

This document can be added to the participants’ folders or emailed to the participants before the event. It provides them with a timeline of what to expect during the event.

Directions: Review this sample and modify contents accordingly to fit your program needs.

# CA- 1 Wellness Family Day

# Date of Event

# Location of Event

|  | **Introduction and Welcome Remarks:** Dr. *Faculty* *Time* |
| --- | --- |
|  | **Day in the Life Video:** moderated by Dr. *Senior Resident Time*  Simulation Experience *Time*  LUNCH: *Name of restaurant providing lunch Time*  Presentation: Burnout, Dr. *Faculty* Time  **Presentation**: Substance Use Disorder, Dr. *Faculty Time*  Presentation: *Institution* Anesthesia Wellness Resources, Dr. *Faculty Time*  Panel of residents/family members: Moderated by Dr. *Faculty Time*  Please complete the survey before leaving. |
|  |  |
