# Supplementary material for: Family Anesthesia Experience: Improving Social Support of Residents Through Education of Their Family and Friends
Source: MedEdPORTAL. 2023 Dec 15;19:11370. doi: 10.15766/mep_2374-8265.11370 (PMC10721742; doi:10.15766/mep_2374-8265.11370)
Supplement: Supplementary file 1 — Preevent FAX Checklist.docxSimulation Setup Instructions.docxSchedule of the Day.docxFAX Timeline.docxDay in the Life.mp4Family Day Simulation Scenario.docxHigh-Fidelity Scenario.mp4High-Fidelity Scenario Part 2.mp4Talking Points for Simulation.docxDidactics.pptxPanel Questions and Logistics.docxPostevent Survey.docx [file mep_2374-8265.11370-s001.zip › F. Family Day Simulation Scenario.docx]

**Family Anesthesia Experience Simulation Scenario – “Perioperative Care of the Surgical Patient”**

This document provides the script and personnel needs for the high fidelity simulation.

**4 Phases of Scenario**

*Preop* = Attending Anesthesiologist, Senior Resident, and standardized patient (SP) as patient ~ 3-4 minutes

*Intraop Induction* = Attending Anesthesiologist, Senior Resident, SP as surgeon, and SP as nurse ~ 3-4 minutes

*Intraop Emergency* = Attending Anesthesiologist, Senior Resident, SP as surgeon, and SP as nurse ~ 7-8 minutes

*Transfer of Care* = Senior Resident calling report with overhead vocal of ICU doctor receiving report ~ 3 minutes

**Logistics**

Room with chairs set up for CA-1 Residents and their support persons to view scenario.

Front left corner for Preop Interview.

General OR setup for other portions of case.

Special Items: code cart, TEE machine (if accessible), blood

1. *Preop Interview* –
   1. Discussion of patient’s past medical history
      1. Large abdominal tumor resection (inferior vena cava invasion)
      2. Severe reflux disease
      3. Coronary artery disease (myocardial infarction with stent 15 months ago).
      4. History of post operative nausea and vomiting
   2. Discussion/Consent of plan
      1. General endotracheal anesthesia, central line, arterial line, possible post-op mechanical ventilation
2. *Intraop Induction of Anesthesia –* BP 120/70, HR 70, Sa02 100%
   1. Rapid sequence induction
   2. Verbally state additional peripheral IV, arterial line, and central line were placed
3. *Intraop emergency* - BP 60/30, HR 110, Sa02 92%
   1. Hypotension
      1. Senior Resident informs the Surgeon.
      2. Senior Resident calls for help and administers vasopressor medications.
         1. Call for TEE
            1. Myocardial infarction

Heparin infusion started

Discussion about whether to abort case or continue

Send patient to ICU postop

1. *Transfer of Care*  - Senior Resident calls report to the ICU using phone

Preop Interview

**SP** – Standardized patient wearing patient gown lying on a stretcher with blanket covering the legs.

**AA** – Attending Anesthesiologist will conduct the preop interview with the patient. AA is wearing scrubs, hat, and mask.

**SR** – Senior Resident will assist with physical exam and preop interview. SR is wearing scrubs, hat, and mask.

1. **AA**: *Hi, Mr(s) Smith, I’m Dr. AA and I will be your anesthesiologist today. I will be taking care of you along with Dr. SR, who is our Anesthesia Resident.*
2. **SP**: *Oh, hi. I am quite nervous about the surgery and anesthesia today, so I am happy to see you both*.
3. **AA**: *Well, let me start out by reviewing some information about your medical history. Then I will answer some of your questions. Then we will talk about the plan for anesthesia and have some time for additional questions if you have any.*
4. **SP**: *Sounds good*.
5. **AA**: *When was the last time you had something to eat or drink?*
   1. **SP**: *I haven’t eaten anything today, and I had a cup of water about 2 hours ago*.
6. **AA**: *Are you allergic to any medications?*
   1. **SP**: *No.*
7. **AA**: *Have you taken any medications today*?
   1. **SP**: *I took my heart medications, metoprolol and lisinopril, and my heartburn medication, Prilosec. I was told I should hold my Plavix this past week, but I have continued my daily baby aspirin*.
8. **AA**: *I see you had a heart a heart attack last year and had a stent placed at that time. How are you doing now? Are you able to walk up 1 to 2 flights of stairs without getting short of breath or chest pain*?
   1. **SP**: *Sometimes. I do have bad joint pains, so walking can be difficult for me. Also, my belly has been hurting from the cancer. But I saw my cardiologist a few weeks ago, and he said he thinks my heart should be fine for the surgery.*
9. **AA**: *I see that you have heartburn. How bad are your symptoms*?
   1. **SP**: *Well, over the past year or so I feel a horrible burning sensation in my throat anytime I try and lay down. In fact, I must sleep in a recliner or else the pain keeps me awake.*
10. **AA**: *I also see you have a history of nausea and vomiting after surgery*.
    1. **SP**: *Yes, I had really bad nausea after my appendix was taken out a few years ago. That was one of my main questions for you actually.*
11. **AA**: *Yes, nausea can sometimes be an issue for patients, but we will talk about a plan to try to prevent the nausea for you in just a moment.*
12. **AA**: *Any other problems with anesthesia?*
    1. **SP***: No, not that I know of.*
13. **SR**: *If it’s ok with you, I would like to take a listen to your heart and lungs.* [SR will do this over 20 seconds).
14. **SR**: *Can you please open your mouth for me? Do you have any issues opening your mouth? Any dentures or loose teeth?*
    1. **SP**: *I don’t have any dentures or loose teeth. I have no issues opening my mouth.*
    2. SR does an airway exam.
15. **AA**: *Ok, well before I describe the anesthesia plan, do you have any other questions?*
    1. **SP**: *No*.
16. **AA**: *Ok, for this surgery we will plan to do general anesthetic* Continue with an explanation of induction, placement of arterial line, central line, plan for extubation, pain control with oral medications and IV medications*.* Do a brief overview of the risks and benefits of general anesthesia (focus on sore throat, damage to mouth, post operative nausea and vomiting and ways to minimize it; blood transfusion, myocardial infarction, stroke).
17. **AA**: *I know that is a lot of information; do you have any other questions for us?*
    1. **SP** – *No, I don’t think so. I understand the plan, and I am ready for surgery.*
18. **SR** – *Great. I’m going to give you a little bit of medication in your IV that will help you relax while we head back to the operating room.*

***Scene change*** Turn off lights for a moment to transition to the OR. Overhead announcement of the patient has been brought to the operating room, positioned on the OR bed, and is ready to go to sleep.

SP 1= Surgeon will turn on the monitor and get ready for induction (i.e., cycle blood pressure cuff, turn on volume of pulse oximeter).

SP 2= Nurse will pull back curtains.

Transition to Operating Room

SP 1 –Surgeon – wearing a surgical gown, hat, mask, gloves.

SP 2 – Nurse – wearing scrubs, hat, mask.

AA - Anesthesiologist standing next to patient’s left arm in scrubs, hat, and mask.

SR – Senior Resident standing at the head of the bed holding an oxygen mask over simulator’s nose and mouth and is wearing scrubs, hat, and mask.

Simulator – The monitors are on the simulator. The monitor is running.

1. **Nurse** – *Ok, is everyone ready for a pre-induction verification on the patient*?
2. **Surgeon, AA, SR** – Each will say *Yes*.
3. **Nurse** – *Great. My name is X. I am the circulator nurse for this surgery*.
4. **Surgeon** *– I am Dr. X, and I will be the surgeon performing the surgery.*
5. **AA** – *I am Dr. AA, and I will be the attending anesthesiologist today.*
6. **SR** – *I am Dr. SR, and I will be the anesthesiology resident today.*
7. **Nurse** – *Our patient is Lee Smith, MRN 123456789, date of birth 1/1/1970, and we are here for the surgical removal of a renal cell carcinoma mass from the abdomen. The patient has no allergies. The plan will be to give 2 grams of Ancef as antibiotics. Are there any other comments?*
8. **Surgeon** – *Yes. This surgery will take about 8 hours. I suspect that the mass has invaded into the IVC and may lead to large amounts of blood loss or major swings in blood pressure.*
9. **AA** –*Thank you for letting us know this information. We will be placing an arterial line and central line after Lee goes off to sleep.*
10. **SR** *– I have 4 units of packed red blood cells and 4 units of FFP in the room just in case we need to give blood products*.
11. **AA** – *We are going to perform a rapid sequence induction and intubation due to Lee having significant GERD symptoms.*
12. **Nurse** – *Great. Then I think we are all on the same page and can get started.*
13. **AA** – *Dr. SR, are you ready for induction of anesthesia*?
14. **SR** – *Yes. I am ready*.
15. **AA** – *Nurse, can you please hold cricoid pressure as Lee falls off to sleep*?
16. **Nurse** – *Sure, no problem*.
17. **AA** – *I am giving 100 mcg of fentanyl, 100 mg of lidocaine, 100 mg of propofol, and 100 mg of succinylcholine now rapidly through the IV.* (Simulator will go apneic, eyes closed, BP decreased to 90/50, HR to 90, Sa02 100.)
18. **SR** – Performs direct laryngoscopy and intubates the patient.
19. **AA** – Looking at the surgeon says – *We will now place an arterial line for blood pressure monitoring* (and activate it on the monitor) *and place a central line so we can give blood products, IV fluids, and blood pressure medications if needed*.
20. **AA** – Looking at the surgeon says – *Ok, the patient is ready for you to start operating now.*
21. **Surgeon** and **Nurse** will then drape the patient.
22. **Surgeon** – *Have the antibiotics been given*?
23. **SR** – *Yes, 2 grams of Ancef have been given*.
24. **Surgeon** –*Great. I am going to make the incision now*.
25. **AA** –Speaking to the SR –*I am going to step out now to see my other patient. Please call me if you need anything. Otherwise, I will be back shortly*.

Transition to Intraop Emergency

Lights turn out briefly. Overhead announcement states, “The surgery has been going well for the first hour. The surgeon is approaching the mass right now”.

No changes in the SP roles. AA will be out of the scene to start while the surgeon is operating.

1. **SR** – Speaking to the surgeon *– Is everything ok with the surgery?*
2. **Surgeon** –While still looking down at the surgical field *– Well, this mass is really hard to get to, and there does seem to be some bleeding*.
3. **SR** –I *thought so. The patient’s blood pressure has fallen significantly over the past minute along with the heart rate going up. I think we may need to transfuse some blood to help support the blood pressure. I am also going to call my attending for some additional help.*
4. **SR** –Pick up the phone and say, *Hey Dr. AA, can you come back to OR 1? The patient is getting unstable from blood loss and I could use some additional help.* Wait about 2 seconds and state, *Great, see you in a moment*. Over the next 10 seconds, SR picks up the first bag of blood and hangs it on the IV pole.
5. **AA** –*Hey Dr. SR, it does seem like the patient is getting unstable. I’m glad you hung a unit of blood. Let’s give a second unit when this one has finished.*
6. As AA is hanging the second unit of blood, SR looks on the monitor and sees that the BP and ETCO2 are unreadable.
7. **SR** – Says out loud, *the blood pressure is unreadable*. And while feeling for a carotid pulse says: *and I don’t feel a pulse. We need to start CPR*. Monitor will read V-fib arrest.
8. **Surgeon** – *I will start chest compressions*. Starts CPR.
9. **AA** – Speaking to Nurse: *Please get the code cart.* Speaking to SR*: Dr. SR, please give 1 mg of epinephrine.*
10. **Nurse** –Returns with the code cart and says: *I will place the pads on the chest*.
11. **AA** –As soon as the pads are on the chest, say to Nurse: *Please prepare the defibrillator to shock the patient at 100 joules.*
12. **Nurse** – While operating the defibrillator says: *Charging*. When the defibrillator is ready says: *Everybody clear. Delivering shock now*. Nurse delivers the shock.
13. **AA** – Directs the surgeon by saying: *Please continue chest compressions until the next pulse check*. Waits 15 seconds and says: *Ok, let’s stop chest compressions for a pulse check.*
14. **SR** –Feels for a carotid pulse and says*: I feel a pulse*. Monitor will display sinus tachycardia with HR 135, BP 170/90, SaO2 97% ETC02 33.
15. **AA** –Speaking to the surgeon says: *I am concerned about the patient, and I am not sure if proceeding is the best thing. Are you at a point where you could stop the surgery if needed?*
16. **Surgeon** –*Yes. I could stop now, and it would be fine to come back when the patient is more stable.*
17. **AA** – *Ok, before proceeding any further, I would like to take a closer look at the patient’s heart with a transesophageal echo.* Nurse to pull out TEE simulator with hypokinesis showing on the monitor. This step can be skipped if TEE simulator is not available.
18. **AA** – *It looks like the patient’s heart is doing really poorly right now. I think we need to abort the case, transfer to the ICU, and have cardiology evaluate the patient.*
19. **Surgeon** –*That is ok with me. I will place a large dressing over the wound, and we can go to the ICU with that in place*.
20. **AA** – *Dr. SR, will you please call report to the ICU, and I will continue monitoring the patien*t.

Calling Report to the ICU

This scene will mostly be the SR calling report to the ICU over the phone with an ICU doctor on the overhead speaker. (The ICU doctor is played by Simulation Technician).

1. **SE** – *Hey Dr. ICU, this is Dr. SR, one of the anesthesia residents. I am calling from OR 1 about a patient who is in the operating room now and needs to come to the ICU*.

- **S**ituation – *She is a 60-year-old female here for a resection of a large abdominal tumor that invades into the IVC*.
- **B**ackground – *She has a history of coronary artery disease and had a stent placed 15 months ago. She also has significant reflux disease. She underwent a general anesthetic with a rapid sequence induction and was an easy intubation. An arterial line and central line were placed after induction. After about an hour of operating, the patient became very unstable, requiring 2 units of blood, and ultimately underwent cardiac arrest. The patient received one round of chest compressions and 1 mg of epinephrine before regaining a pulse. A TEE was done and showed global hypokinesis. We have aborted the case and are coming to the ICU*.
- **A**ssessment – *The patient is currently intubated and on an epinephrine infusion. Her mean arterial pressures are in the 60s. An ABG has been sent but the results have not come back yet*.
- **R**ecommendations – *We will be transporting the patient to you in just a few minutes, intubated and sedated. She has 2 more units of blood available in the blood bank. Cardiology has been consulted and they plan to see the patient immediately upon arrival to the ICU. Please have an EKG machine ready upon arrival for the cardiology team. The patient’s husband is available by a phone number listed in her chart. Do you have any questions for me at this point*? Wait a few moments. *OK great. We will see you shortly*.

END OF SCENARIO
