# Supplementary material for: Family Anesthesia Experience: Improving Social Support of Residents Through Education of Their Family and Friends
Source: MedEdPORTAL. 2023 Dec 15;19:11370. doi: 10.15766/mep_2374-8265.11370 (PMC10721742; doi:10.15766/mep_2374-8265.11370)
Supplement: Supplementary file 1 — Preevent FAX Checklist.docxSimulation Setup Instructions.docxSchedule of the Day.docxFAX Timeline.docxDay in the Life.mp4Family Day Simulation Scenario.docxHigh-Fidelity Scenario.mp4High-Fidelity Scenario Part 2.mp4Talking Points for Simulation.docxDidactics.pptxPanel Questions and Logistics.docxPostevent Survey.docx [file mep_2374-8265.11370-s001.zip › A. Preevent FAX Checklist.docx]

**Pre-event FAX Checklist**

Reservations

- Send out a “Save the Date” 4 months prior to the event
- Solicit RSVPs from residents and their support persons. Invitations can include a schedule of the day. Residents can have as many support persons as your program allows. *Optional* send calendar invites upon confirmation of attendance.

Event Staffing (10 or more)

*Below are suggestions. Please altar according to your event needs. Additional help can also be utilized!*

- 3-4 faculty members
- 3 upper-level residents with their support persons
- 2-3 standardized actors
- 1 simulation technician
- 1 person to help with set-up and materials

**Description of roles**

- Faculty members will welcome guests, introduce the event, and deliver the didactics on burnout, substance use disorder and wellness, and moderate resident/support person panel. They can assist with simulation stations as needed.
- An upper-level resident is used to narrate the “Day in the Life” video
- 5-6 people (faculty, upper-level resident, 2-3 standardized patients, simulation technicians) are needed for the high-fidelity scenario. If you are showing this scenario in a simulated operating room rather than playing the video from Appendices G & H, you will need one or two additional standardized patients to play the role of circulating nurse and surgeon. Since there is a transition from the preop area to the operating room, the standardized patient playing the role of the patient can switch to the role of the nurse or surgeon if needed.
- 3 faculty or upper-level residents are needed to manage the 3 task trainers (central line, peripheral nerve block, neuraxial).
- 3 residents (CA-2s and CA-3s) and their support persons for the panel discussion. Ideally, the residents’ support persons should be present, and the panel should consist of different types of support persons (i.e., significant other, parents, children).
- 1 person is needed to manage all the materials (check-in, surveys, forms, guest packets, food set up, photos, room set up, keeping time etc.)

Event Content

- Modify didactics PowerPoint for your program
  - Slide 1: change to fit your program
  - Slide 3: change to your department demographics
  - Slide 7: use your “Day in the Life” video
  - Slide 8: change to your presenter's name
  - Slide 12: change to your presenter's name
  - Slide 20: change to your presenter’s name
  - Slides 37-45: update to represent your institution/department resources
  - Slide 46 and 48: change to your institution's faculty
  - Slide 47: replace with photos of your panel
- Share the didactic PowerPoint (Appendix J) with presenters so they can review the material before the event. Share talking points that match the simulations (Appendix I) with faculty and residents who will be assisting with the simulation portion of the event.
- Create “Day in the Life” video (can use UNC’s [Appendix E] if unable to create your own video).
- Once you have the RSVP list, create a new list that divides the participants into 3 groups, keeping residents with their support persons. This list will be used during the simulation part of the event.
