# Supplementary material for: Family Anesthesia Experience: Improving Social Support of Residents Through Education of Their Family and Friends
Source: MedEdPORTAL. 2023 Dec 15;19:11370. doi: 10.15766/mep_2374-8265.11370 (PMC10721742; doi:10.15766/mep_2374-8265.11370)
Supplement: Supplementary file 1 — Preevent FAX Checklist.docxSimulation Setup Instructions.docxSchedule of the Day.docxFAX Timeline.docxDay in the Life.mp4Family Day Simulation Scenario.docxHigh-Fidelity Scenario.mp4High-Fidelity Scenario Part 2.mp4Talking Points for Simulation.docxDidactics.pptxPanel Questions and Logistics.docxPostevent Survey.docx [file mep_2374-8265.11370-s001.zip › B. Simulation Setup Instructions.docx]

Simulation Set Up

This document provides details on how to set up the hands-on stations for the Family Anesthesia Experience.

**Station 1: High Fidelity Scenario**:

The scenario starts with a preoperative assessment of the patient by the resident. The scene changes to the operating room and demonstrates induction of anesthesia, an intraoperative code with resolution, and ends with a phone hand off to the ICU.

There are two ways you can run this scenario.

1. An attending, resident and standardized patient performs the pre-operative interview live and then present the High-Fidelity Scenario video (Appendices G and H) on a TV.
2. Run the whole scenario live from pre-operation to post-operation, using additional standardized patients to play the roles of circulating nurse and surgeon.


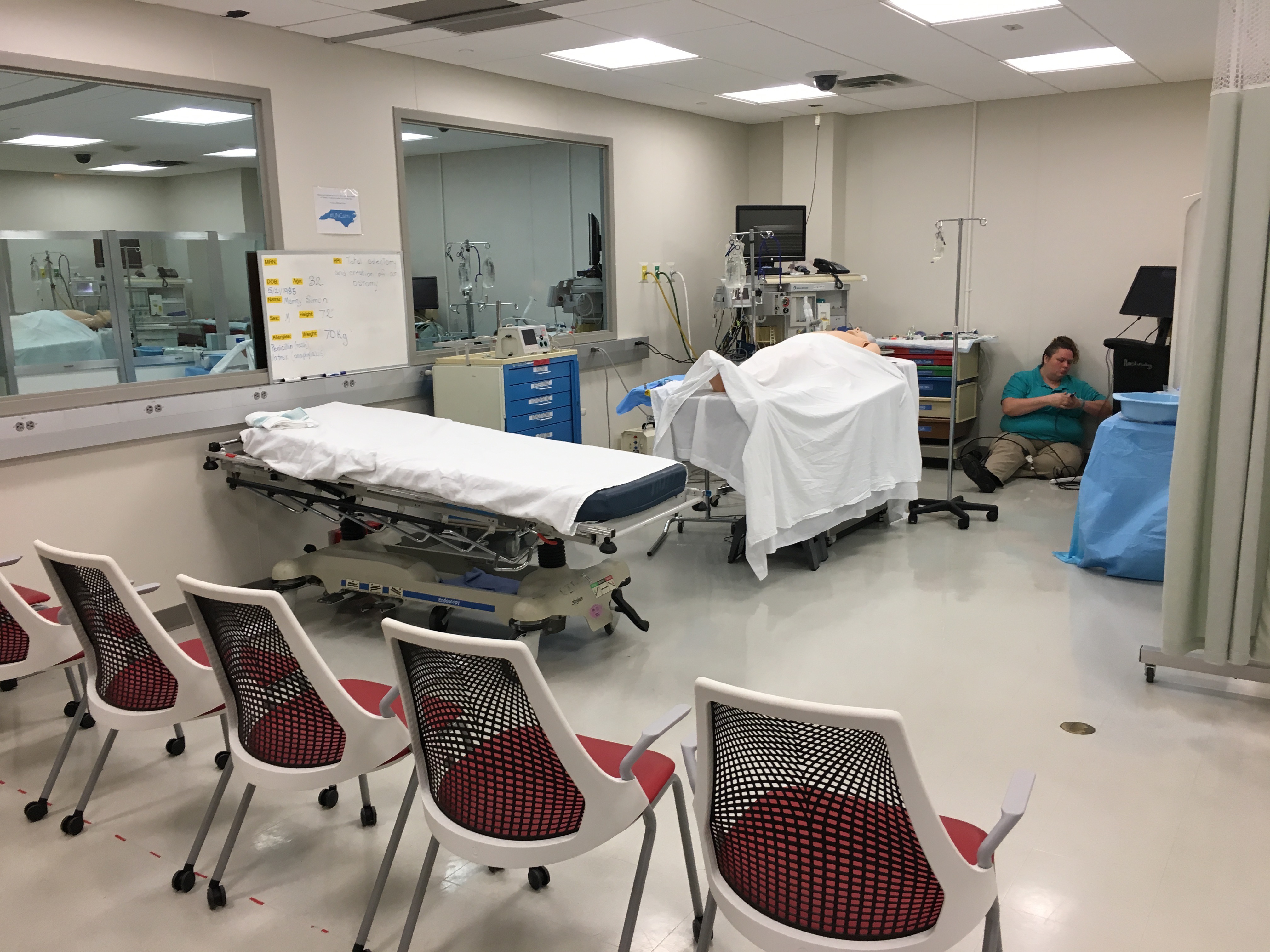


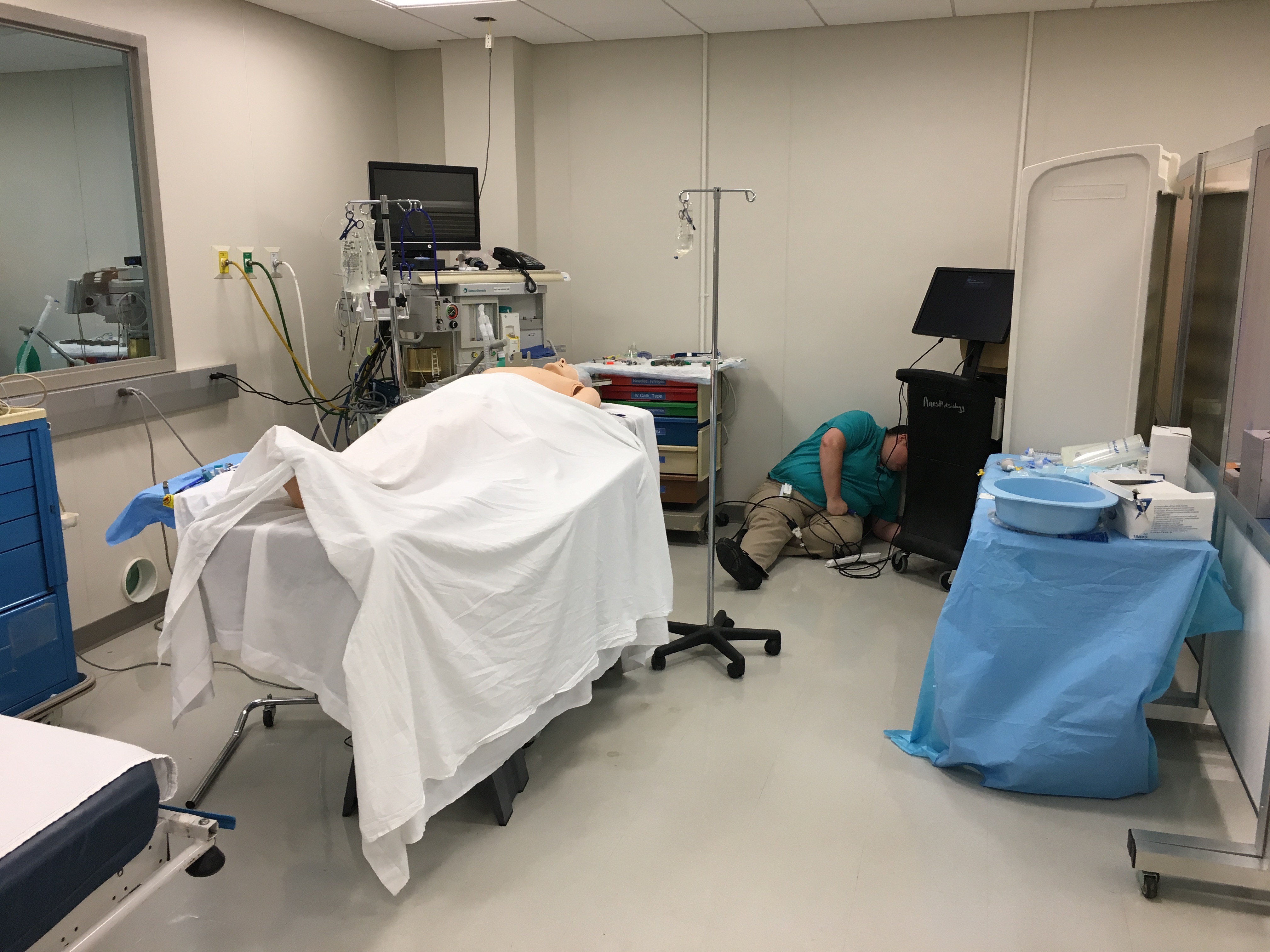


- Set up enough chairs for observers
- Stretcher with pillow, blanket, patient gown, and standardized patient who will be used for the preoperative interview.

**Version 1**

One standardized patient will play the role of a patient.

Following the preoperative assessment, the video of the high-fidelity scenario will be played.

**Version 2**

Notice the curtain on the right-hand side of the picture. This curtain is pulled closed during the preoperative interview, so viewers are not distracted by the operating room simulation setup.

Operating room simulation setup – OR bed, High fidelity mannequin (e.g. Laerdal SimMan3G) with anesthesia machine, non-intubated patient (will be intubated during the scenario), syringes, monitor (vital signs changes on the script).

The monitor on the cart in the back right is the HeartWorks TEE simulator. This is used to display wall motion abnormalities after the intraoperative code. This could be replaced with a video clip of TEE pathology playing on a screen if needed or left out completely.

Two standardized patients will play the roles of circulating nurse and surgeon. One of these could have also played the role of patient in the preop component.

An upper-level resident should play the role of the in-room anesthesia provider. An attending anesthesiologist should play the role of the attending anesthesiologist.

**Both Versions**

Once the scenario ends (usually runs approximately 15 minutes), the attending and senior resident will lead a debriefing with a Q & A session with the CA-1 residents and their support persons. See Appendix I for talking points and questions.

**Station 2: Airway:**

CA-1 residents do most of the teaching in the airway stations. A faculty member is present to help as needed. See Appendix I for talking points.

This group will be split into 3 smaller groups. Each small group should spend 10 minutes at each of the 3 airway stations. Someone should keep track of time and rotate the groups.

**Sub-Stations**:

1. *Bag mask ventilation, oral airways, nasal trumpets*

Items needed: intubating mannequins, self-inflating resuscitator bags, masks, nasal trumpets, oral airways


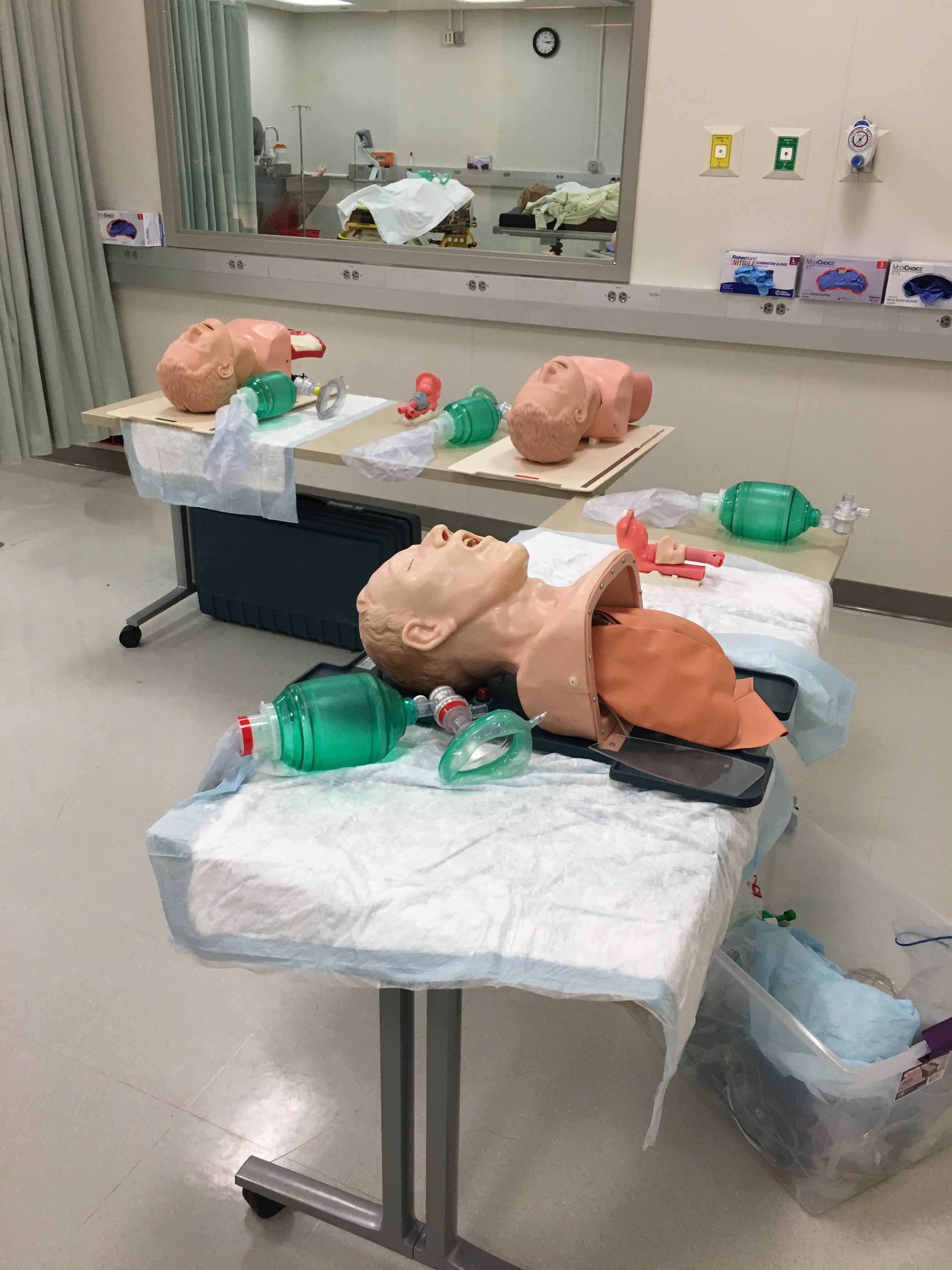


1. *Direct laryngoscopy/LMA*

Items needed – intubating mannequins, self-inflating resuscitator bags, laryngoscopes, ETT, stylets, LMAs


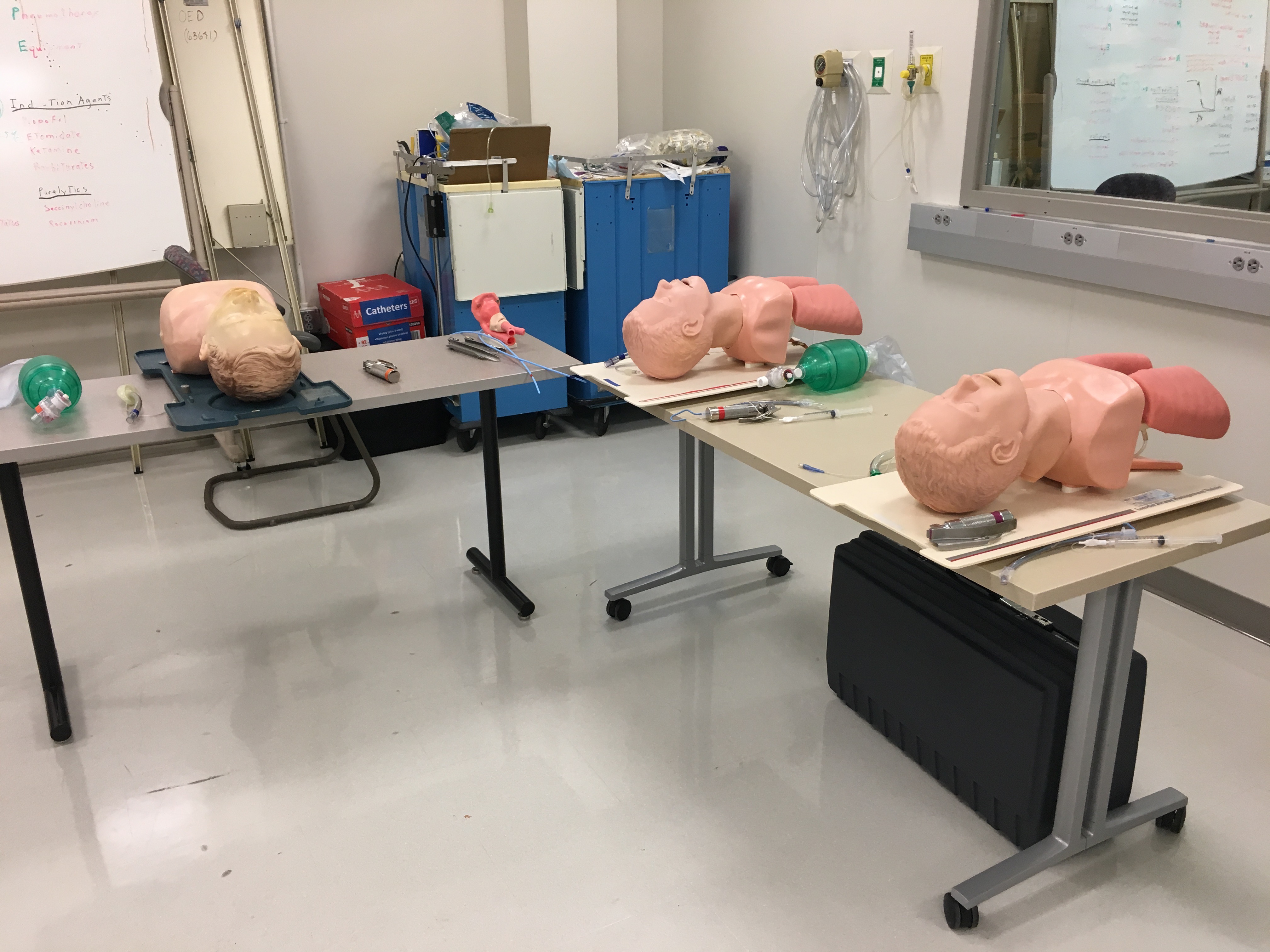


1. *Advanced airway devices* – video laryngoscopes/glidescope, fiberoptic (fiberoptic simulator shown below).

Items needed - intubating mannequin, self-inflating resuscitator bag, advanced airway equipment available at your institution, c-collar, ETT


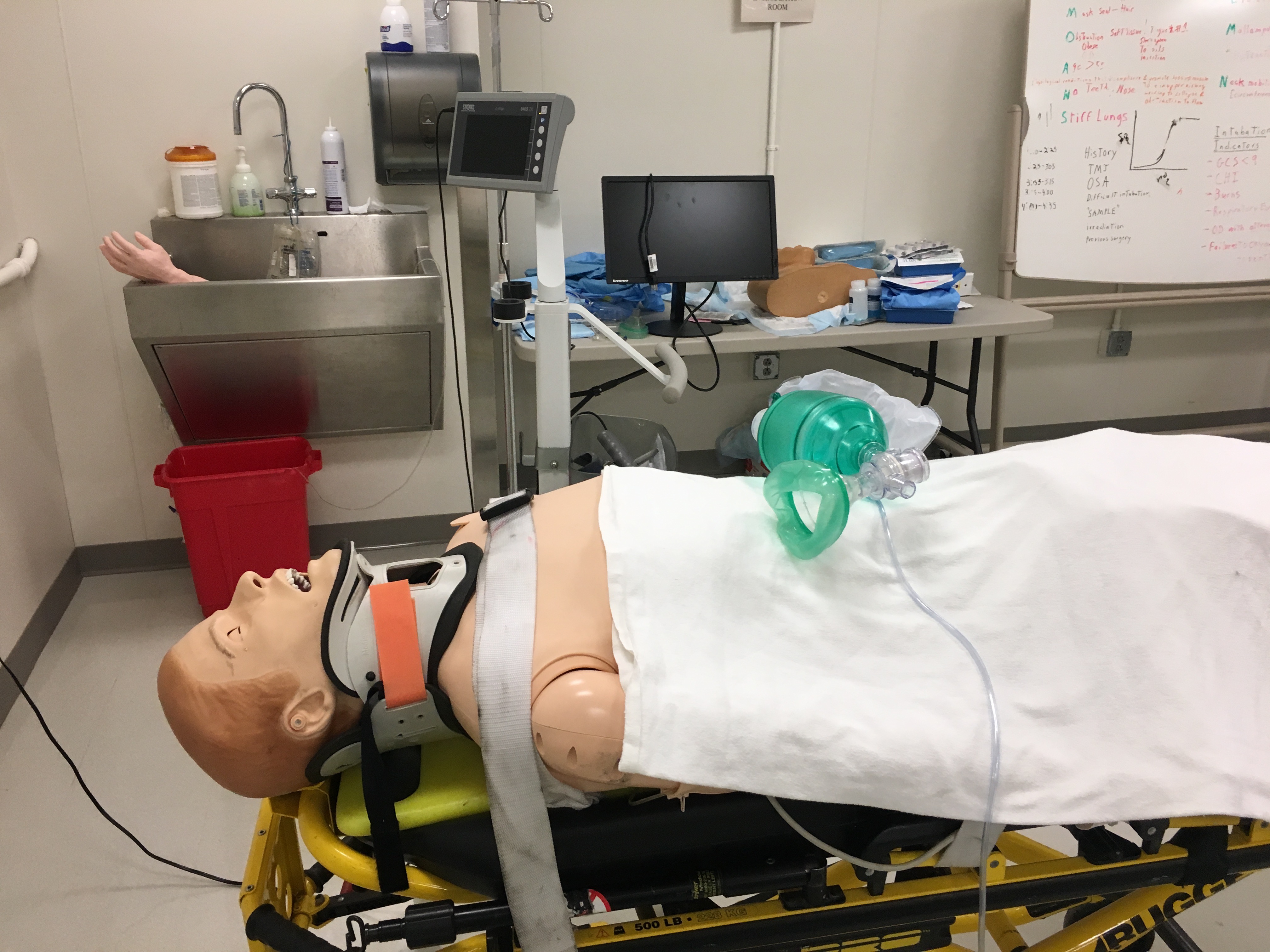


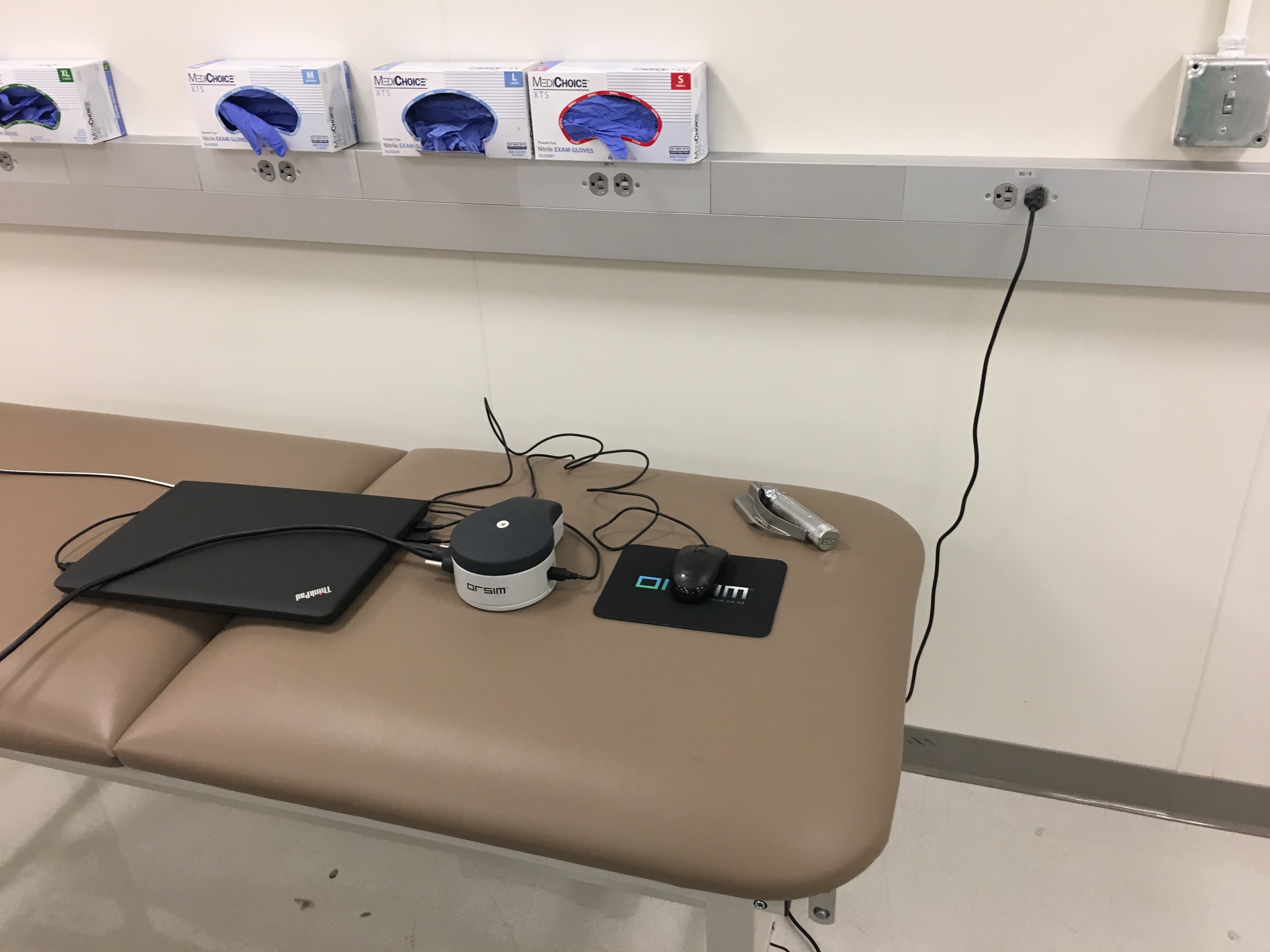


**Station 3: Task trainers:**

Senior residents and faculty do the teaching at each of these stations since the CA-1 residents don’t have enough knowledge in these areas yet. See Appendix I for talking points.

The group is split into 3 smaller groups. Each small group spends 10 minutes at each of the 3 stations. Someone should keep track of time and rotate the groups.

1. *Central line* – Ultrasound guided Right Internal Jugular

Items needed – ultrasound, ultrasound gel, towels, central line kit, task trainer, fluids for task trainer


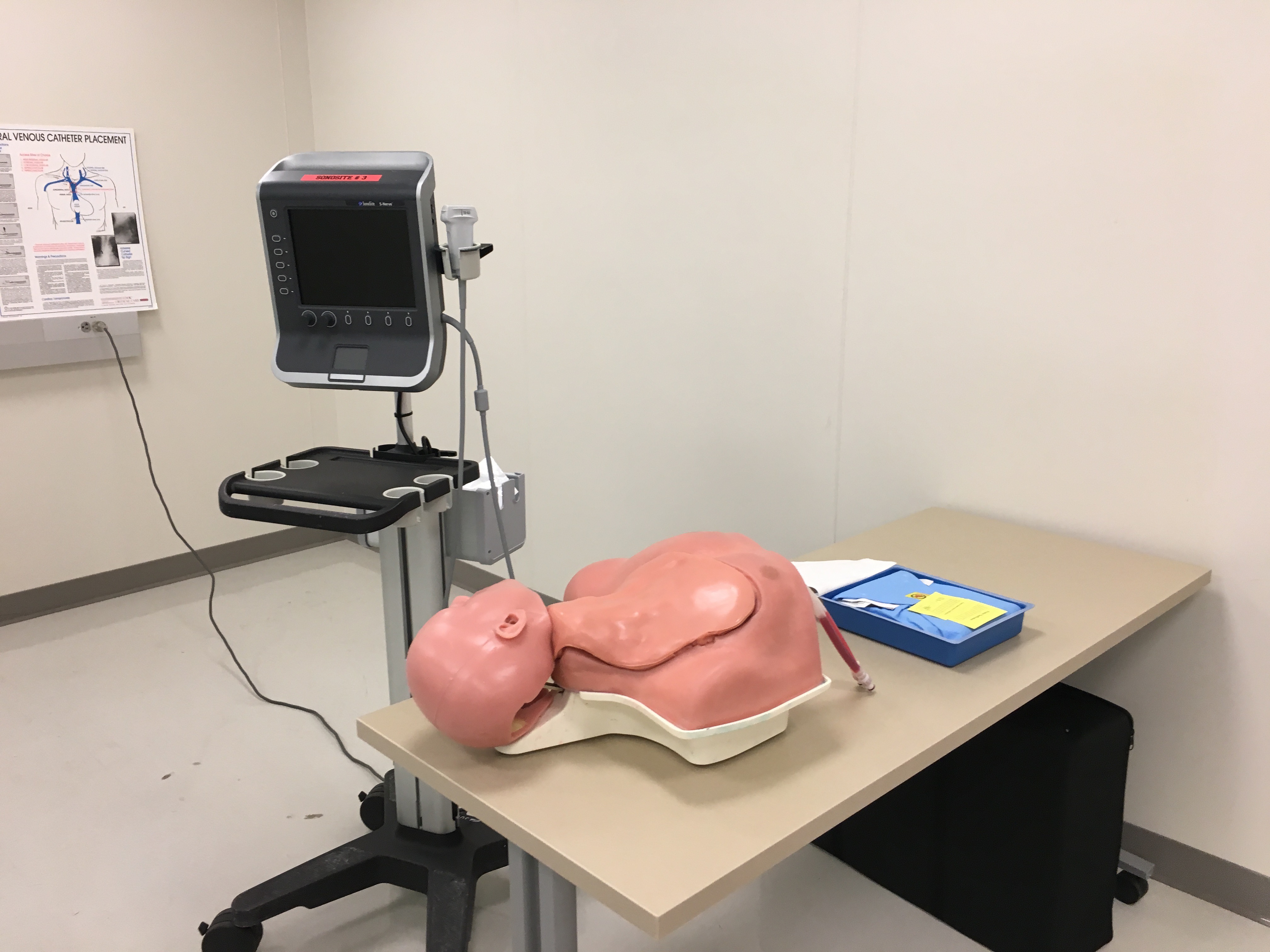


1. *Peripheral nerve blocks* – Right interscalene and supraclavicular nerve blocks

Items needed – ultrasound, ultrasound gel, towels, nerve block kit, task trainer, fluids for task trainer


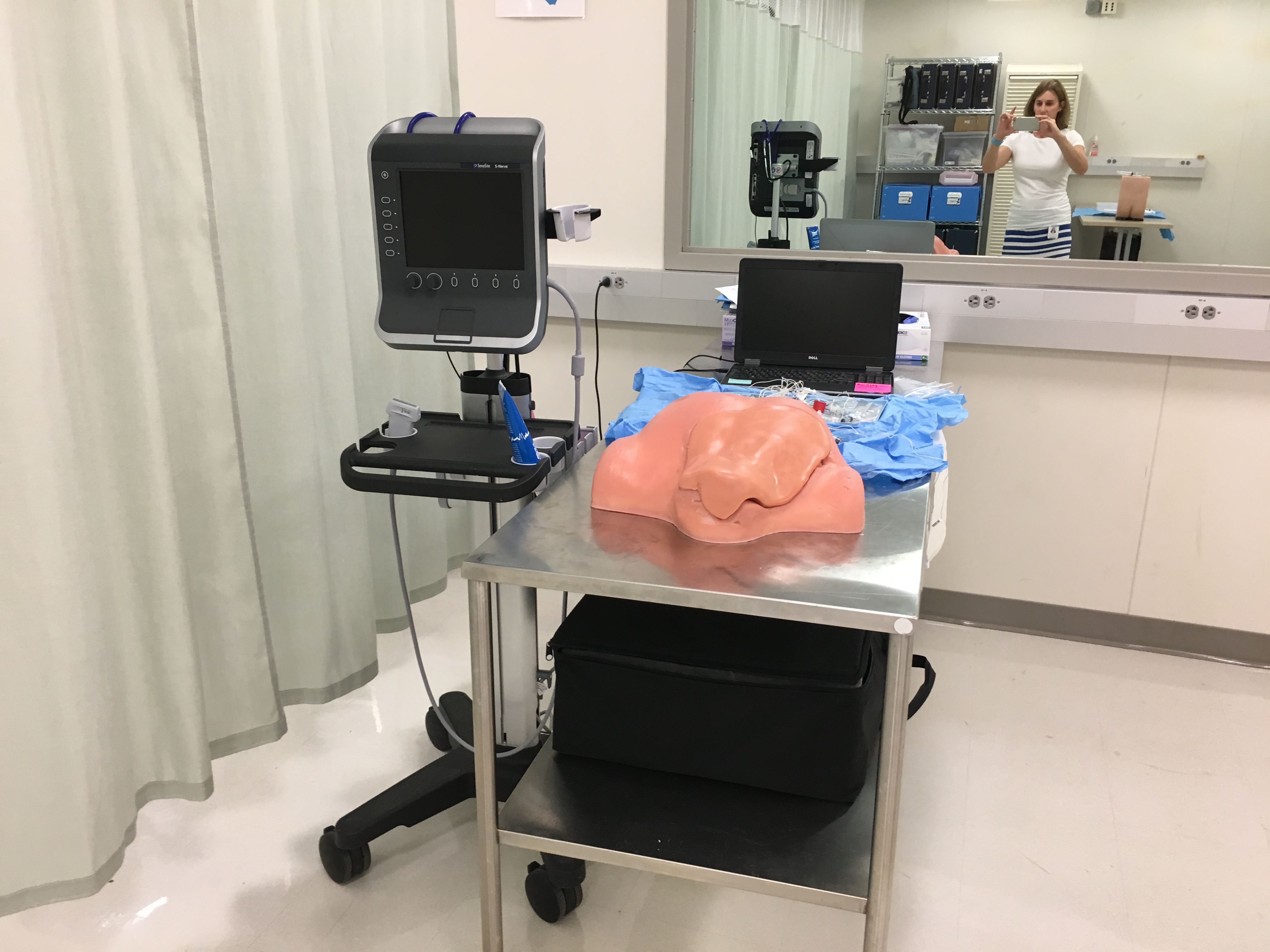


1. *Neuraxial blocks* – epidural, spinal, and combined spinal epidural

Items needed – epidural task trainer, neuraxial block kits


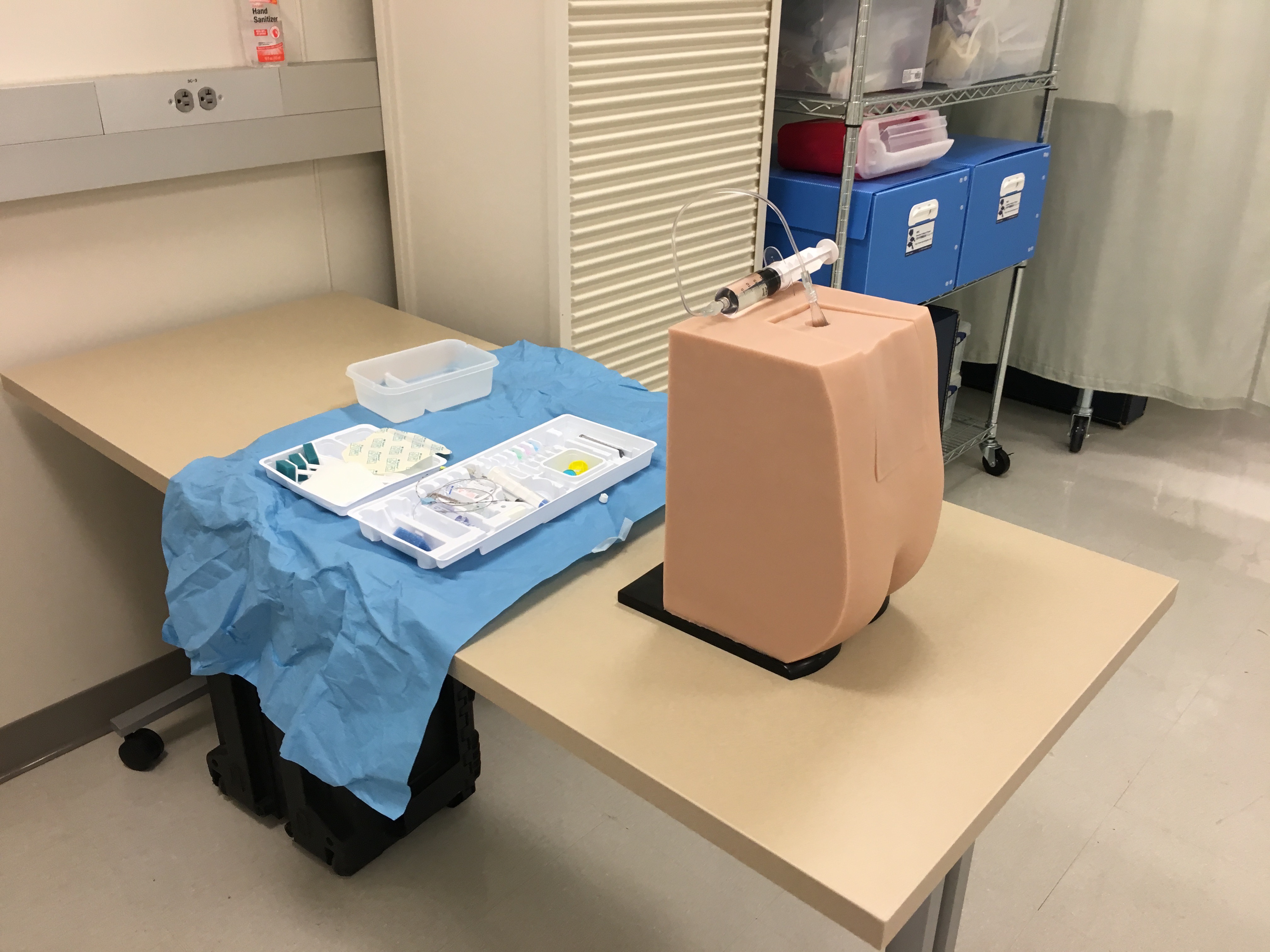


All photographs are author owned.
