# Supplementary material for: Family Anesthesia Experience: Improving Social Support of Residents Through Education of Their Family and Friends
Source: MedEdPORTAL. 2023 Dec 15;19:11370. doi: 10.15766/mep_2374-8265.11370 (PMC10721742; doi:10.15766/mep_2374-8265.11370)
Supplement: Supplementary file 1 — Preevent FAX Checklist.docxSimulation Setup Instructions.docxSchedule of the Day.docxFAX Timeline.docxDay in the Life.mp4Family Day Simulation Scenario.docxHigh-Fidelity Scenario.mp4High-Fidelity Scenario Part 2.mp4Talking Points for Simulation.docxDidactics.pptxPanel Questions and Logistics.docxPostevent Survey.docx [file mep_2374-8265.11370-s001.zip › D. FAX Timeline.docx]

**Family Anesthesia Experience Timeline**

This document provides a timeline for the different components of the Family Anesthesia Experience.

9:45-10:00 CA-1 Residents and their Support Persons arrive

10:00-10:03 Introduction (by a faculty member)

10:04-10:20 Day in the Life Video (moderated by senior resident)

10:21-12:00 Simulation (introduction given by faculty member)

12:01-12:45 Lunch (boxed lunches provided by a local restaurant)

12:46-1:10 Didactics (by faculty members)

1:11-1:50 Panel of Senior Residents and their Support Persons (moderated by faculty member)

1:51-2:00 Wrap Up (by faculty member) and survey completion
